# Tridentate 3-Substituted Naphthoquinone Ruthenium Arene Complexes: Synthesis, Characterization, Aqueous Behavior, and Theoretical and Biological Studies

**DOI:** 10.1021/acs.inorgchem.1c01083

**Published:** 2021-06-11

**Authors:** Heiko Geisler, Julia Westermayr, Klaudia Cseh, Dominik Wenisch, Valentin Fuchs, Sophia Harringer, Sarah Plutzar, Natalie Gajic, Michaela Hejl, Michael A. Jakupec, Philipp Marquetand, Wolfgang Kandioller

**Affiliations:** † Faculty of Chemistry, Institute of Inorganic Chemistry, University of Vienna, Waehringer Str. 42, 1090 Vienna, Austria; ‡ Department of Chemistry, University of Warwick, Gibbet Hill, Coventry, CV47AL, United Kingdom; § Research Cluster “Translational Cancer Therapy Research”, University of Vienna, Waehringer Str. 42, A-1090 Vienna, Austria; ∥ Faculty of Chemistry, Institute of Theoretical Chemistry, University of Vienna, Waehringer Str. 17, A-1090 Vienna, Austria; ⊥ Vienna Research Platform on Accelerating Photoreaction Discovery, University of Vienna, Währinger Str. 17, 1090 Wien, Austria

## Abstract

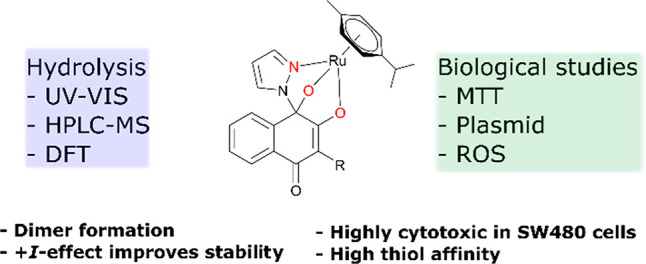

A series of nine Ru^II^ arene complexes bearing tridentate naphthoquinone-based *N*,*O*,*O*-ligands was synthesized and characterized. Aqueous stability and their hydrolysis mechanism were investigated via UV/vis photometry, HPLC-MS, and density functional theory calculations. Substituents with a positive inductive effect improved their stability at physiological pH (7.4) intensely, whereas substituents such as halogens accelerated hydrolysis and formation of dimeric pyrazolate and hydroxido bridged dimers. The observed cytotoxic profile is unusual, as complexes exhibited much higher cytotoxicity in SW480 colon cancer cells than in the broadly chemo- (incl. platinum-) sensitive CH1/PA-1 teratocarcinoma cells. This activity pattern as well as reduced or slightly enhanced ROS generation and the lack of DNA interactions indicate a mode of action different from established or previously investigated classes of metallodrugs.

## Abstract 2

Tridentate 3-substituted naphthoquinone organometallics show strong cytotoxicity in chemo-resistant colon cancer cells. The aquation pathway, the behavior in aqueous solutions, and the preliminary mode of action studies are presented.

## Introduction

**Figure 1:**
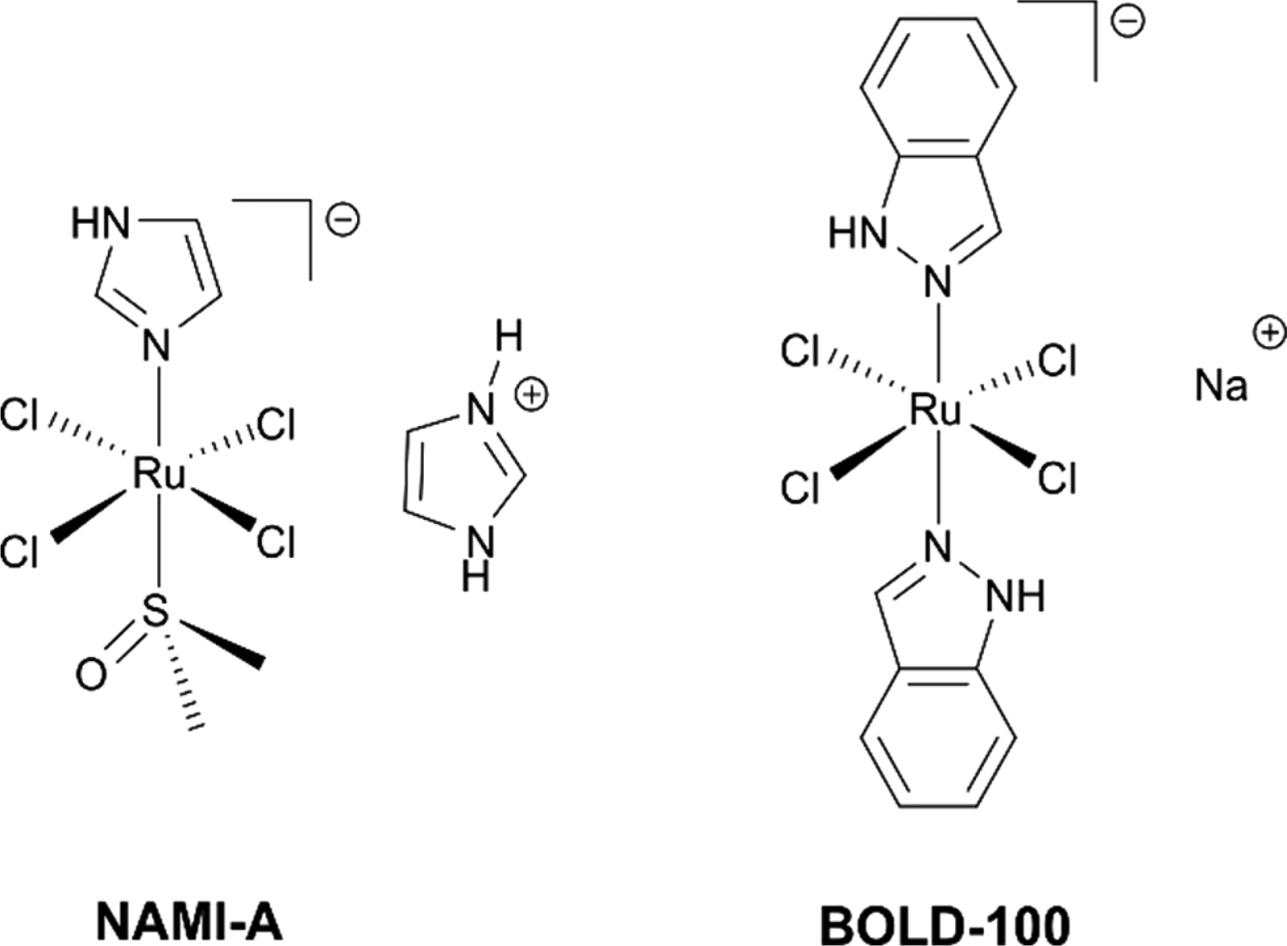
Structural formulas of clinically studied Ru^III^ compounds.

Metallodrugs have played an important role in medicine for centuries and are essential for several therapeutic and diagnostic applications.^1^ The discovery of salvarsan by Paul Ehrlich and his description of the medical effect of arsenic compounds against syphilis in 1912 could be designated as the advent of modern chemotherapy.^2^ Due to their various pharmaceutical properties (e.g., antimicrobial, antiparasitic, antarthritic, antidiabetic, antiviral, and anticancer), metal-based compounds are a standard in modern medicine.^3^ The anticancer activity of cisplatin was incidentally discovered by Barnett Rosenberg in 1969 and provided the groundwork for metal-based anticancer agents.^3^ Currently, three Pt^II^ compounds (cisplatin, carboplatin, and oxaliplatin) are approved for clinical treatment worldwide. Despite the discovery that these drugs cause severe side effects (such as nephrotoxicity, myelosuppression, and neurotoxicity), they are still used as first-line agents in various cancer treatment regimens.^4^ Nonetheless, researchers are focused on identifying novel metallodrugs to overcome these drawbacks. Due to a wide range of stable oxidation states under biologically relevant conditions, acceptable ligand exchange rates, and a rich coordination chemistry, other metals of the platinum group were identified as possible alternatives.^5,6^ The most auspicious representatives are ruthenium coordination compounds, which have shown promising results in (pre)clinical trials.^7^ In this context, BOLD-100 (formerly, KP1339/NKP1339/IT-139) and NAMI-A are well studied representatives of octahedral Ru^III^ complexes (Figure 1). NAMI-A showed activity against metastases in preclinical settings, where it reduced the growth and formation of lung metastases in malignant tumors.^8,9^

**Figure 2:**
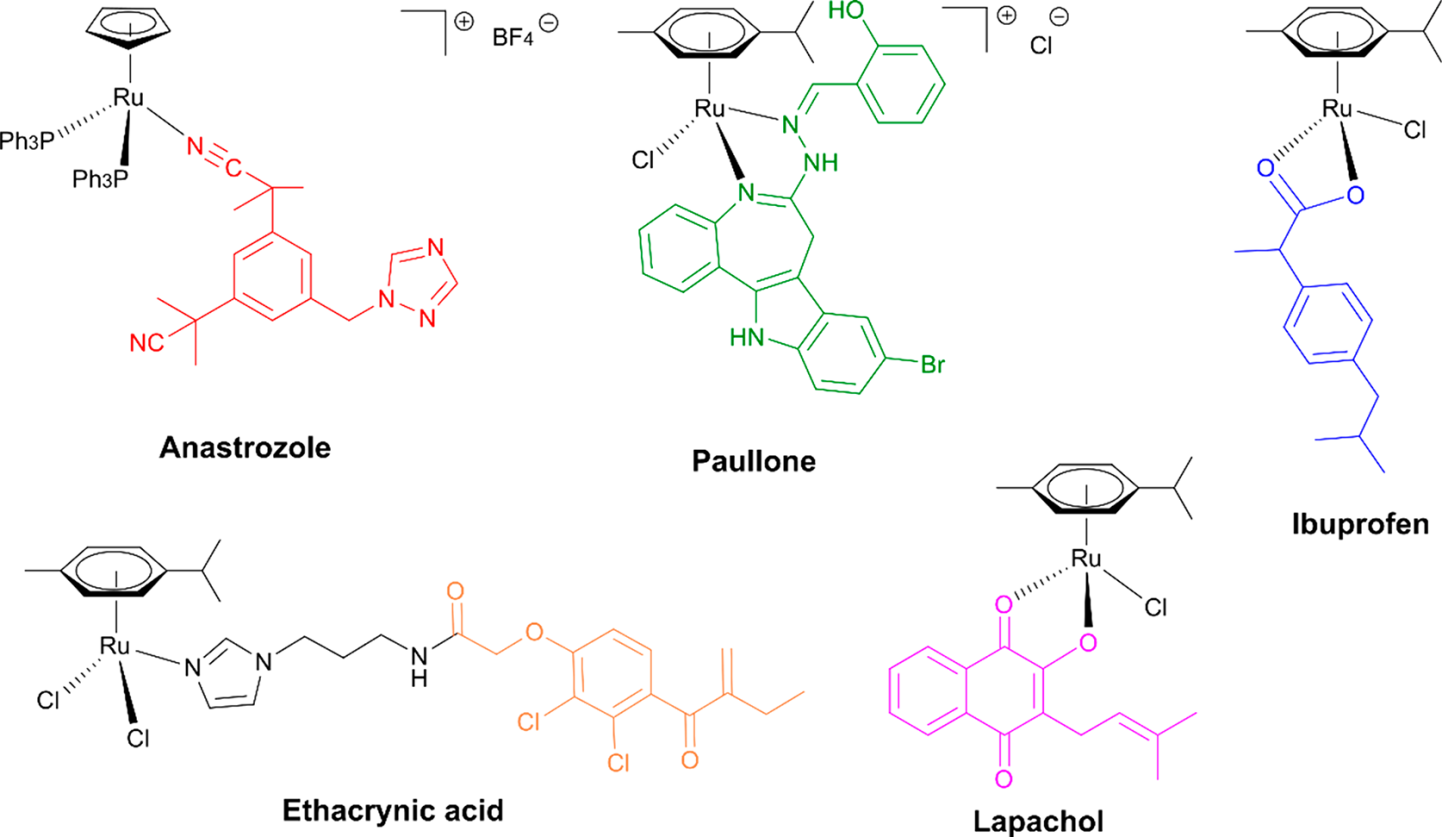
Organometallics with bioactive ligands: aromatase inhibitor (red), CDK inhibitor (green), COX inhibitor (blue), GST inhibitor (orange), naphthoquinone (pink).

This compound successfully passed phase I clinical trials; however, due to limited efficacy in a phase II trial, further clinical investigations were discontinued.^7^ BOLD-100’s mode of action is still not fully elucidated, but studies have shown that this compound inhibits GRP78 and interferes with endoplasmic reticulum homeostasis and ribosomal proteins, resulting in cell death.^10−12^ Besides Ru^III^ coordination compounds, the potential of Ru^II^ (organometallic) complexes is currently under investigation. TLD1443 is a promising Ru^II^ coordination compound in clinical trials and is investigated for application in photodynamic therapy (PDT) treatment of non-muscle invasive bladder tumors.^13,14^ Another intensively studied compound class are pseudo-octahedral Ru^II^ organometallics. The geometry of organoruthenium piano-stool complexes allows easy modification of the ligand sphere and thus fine-tuning of the compounds’ pharmacokinetic and pharmacodynamic properties. In the past, promising results have been obtained for organoruthenium compounds, such as RAPTA (RAPTA = [Ru^II^(arene)(pta)Cl_2_]) or RAED complexes (RAED = [Ru^II^(arene)(ethylenediamine)Cl]^+^), which are at an advanced preclinical stage.^15−18^ Apart from these examples, several organometallics with promising *in vivo* activities were reported in the literature.^19−21^ Furthermore, the development of metal complexes containing bioactive ligands gains more attention, as these substances may act as multitargeted drugs. They could provide selectivity through specific interactions with enzymes, proteins, or other biomolecules and might lead to enhanced drug potency and circumvention of drug resistances, as well as side effects.^18,22^ Hence, many research groups used the multitargeted approach and developed organometallics bearing aromatase,^23^ CDK (cyclin-dependent kinase),^24^ COX (cyclooxygenase),^25^ or GST (glutathione-S-transferase) inhibitors (Figure 2).^26^

Naphthoquinones exhibit a broad range of biological activities, such as antibacterial, antifungal, antiparasitic, antiviral, and antitumoral.^27^ The pharmacological profile of this compound class arises from different biological effects, as they can generate ROS (reactive oxygen species), interact with NQO1 (NAD(P)H dehydrogenase), regulate expression of p53 and tumor-associated inflammation, inhibit topoisomerases and MALT1 (mucosa-associated lymphoid tissue lymphoma translocation protein 1), induce apoptosis, or suppress telomerase activity.^28^ As a consequence, organometallics bearing 2-hydroxy-1,4-naphthoquinones were investigated *in vitro* and *in vivo* (Figure 2).^29−33^ Furthermore, Ru^II^ coordination compounds bearing lapachol and lawsone as bidentate ligands have been reported as highly cytotoxic in cisplatin-resistant cell lines.^34^ In another work by Biersack and co-workers, plumbagin was linked via a hydrazide group to pyridine and monodentately coordinated to organometallic scaffolds, yielding compounds with pronounced cytotoxicity and Pgp inhibition capability.^35^

In previous studies, we reported on organoruthenium and -osmium complexes bearing an *in situ* generated tridentate naphthoquinone scaffold, which were characterized by enhanced stability in aqueous media compared to their bidentate naphthoquinone analogues featuring a halido leaving group.^29^ Additionally, they revealed a striking cytotoxic profile, as they were highly cytotoxic in more chemo-resistant cancer cells (SW480, A549), while their activity was markedly reduced in broadly chemo-sensitive cells (CH1/PA-1).

Within this work, a series of tridentate metal complexes with different hydroxy-1,4-naphthoquinones was synthesized (**1a**–**9a**, Scheme 2) to investigate the influence of the performed modifications on stability and anticancer properties. Furthermore, theoretical studies were conducted to confirm the observed behavior in aqueous solution and the postulated mode of aquation. In order to elucidate their biological behavior, MTT assays in 2D cell cultures were carried out and ROS formation as well as DNA and amino acid interactions were studied via the DCFH-DA assay, electrophoretic plasmid assay, and HPLC-MS measurements, respectively.

## Experimental Section

### Syntheses and Characterization

Solvents were purchased from commercial suppliers and dried before use if needed. Microwave reactions were performed with a Biotage Initiator+ system. Purification via flash column chromatography was conducted with a Biotage Isolera system and silica gel (VWR, mesh 40–63 μm). The dimeric metal precursor [Ru(*p*-cymene)Cl_2_]_2_ was synthesized according to the literature.^36^ The general numbering of carbons and hydrogens and the corresponding NMR spectra of ligands **2**–**9** and complexes **1a**–**9a** were described in the Supporting Information (pp 3–16). ^1^H, ^13^C, and 2D NMR spectra were recorded at 298 K on a Bruker AV III 600 or AV NEO 500 spectrometer at 600.25/500.10 MHz (^1^H) and 150.95 MHz (^13^C). Elemental analyses were conducted by the microanalytical laboratory of the faculty of chemistry of the University of Vienna with a Eurovector EA 3000 (2009) equipped with a high temperature pyrolysis furnace (HT, Hekatech, Germany, 2009).

### Hydroxy-1,4-naphthoquinone Syntheses (**2**–**9**)

#### 2-Hydroxy-3-methylnaphtalene-1,4-dione (**2**)^37,38^

2-Methyl-1,4-naphthoquinone (1.54 g, 8.93 mmol) was suspended in 100 mL of methanol and cooled to 0 °C. Sodium carbonate (0.28 g, 2.68 mmol) and hydrogen peroxide solution (30%, 1.72 mL, 517 mg, 15.19 mmol) were dissolved in 10 mL of water and added slowly to the suspension. The mixture was stirred for 0.5 h at 0 °C and for another 1 h at room temperature. Methanol was removed (20–30 mL) by reduced pressure, and subsequently, water was added for precipitation. The white precipitate was separated, washed with water, and dried *in vacuo*. The epoxide was suspended in THF (120 mL), and ca. 4 g of silica gel and conc. H_2_SO_4_ (1.76 mL, 3.24 g, 33.05 mmol) were added. The mixture was evaporated at 500 mbar and 70 °C until dryness. The formed yellow solid was dissolved in dichloromethane, filtrated, and washed with saturated sodium bicarbonate solution. The dark red aqueous layers were combined and acidified with concentrated HCl. The yellow suspension was extracted with dichloromethane, dried over anhydrous Na_2_SO_4_, and evaporated and dried *in vacuo*. Yield: 1.33 g of yellow powder (7.07 mmol, 79%). ^1^H-NMR (500.10 MHz, CDCl_3_) δ 8.13 (dd, *J* = 7.8, 1.1 Hz, 1H), 8.08 (dd, *J* = 7.7, 1.7 Hz, 1H), 7.75 (ddd, *J* = 7.6, 7.5, 1.4 Hz, 1H), 7.68 (ddd, *J* = 7.6, 7.5, 1.3 Hz, 1H), 7.29 (s, 1H), 2.11 (s, 3H). Elemental analysis found: C, 69.82; H, 4.23; O, 25.49. Calcd for C_11_H_8_O_3_: C, 70.21; H, 4.29; O, 25.51%.

#### 2-Ethyl-3-hydroxynapthalene-1,4-dione (**3**).^39^

**1** (200 mg, 1.15 mmol), Hantzsch ester (322 mg, 1.27 mmol), and acetaldehyde were suspended in 12 mL of dry dichloromethane. After the addition of l-proline (26 mg, 0.23 mmol), the solution was stirred for 24 h at room temperature. The mixture was purified by flash column chromatography (SiO_2_) with DCM/*n*-hexane (40–100% DCM), and the yellow crystalline product was dried *in vacuo*. Yield: 185 mg of yellow crystals (0.91 mmol, 79%). ^1^H-NMR (600.25 MHz, CDCl_3_) δ 8.13 (dd, *J* = 7.6, 1.3 Hz, 1H), 8.08 (dd, *J* = 7.6, 1.3 Hz, 1H), 7.75 (ddd, *J* = 7.6, 7.6, 1.3 Hz, 1H), 7.68 (ddd, *J* = 7.5, 7.5, 1.3 Hz, 1H), 7.28 (s, 1H), 2.63 (q, *J* = 7.5 Hz, 2H), 1.15 (t, *J* = 7.5 Hz, 3H). Elemental analysis found: C, 70.86; H, 4.95; O, 23.86. Calcd for C_12_H_10_O_3_: C, 71.28; H, 4.98; O, 23.73%.

#### 2-Cyclohexyl-2-hydroxynaphtalene-1,4-dione (**4**).^40^

**1** (312 mg, 1.79 mmol), Fe(NO_3_)_3_·9H_2_O (3615 mg, 8.95 mmol), and cyclohexene (725 μL, 588 mg, 7.16 mmol) were dissolved in 20 mL of acetonitrile/ethanol mixture (1:1). The brown mixture was stirred at room temperature for 5 min, and subsequently, sodium borohydride (271 mg, 7.16 mmol) was added in portions carefully. After 2 h, the yellow mixture was quenched with 5% HCl (30 mL) and extracted with dichloromethane (90 mL). The organic layer was purified by flash column chromatography (SiO_2_) with EtOAc/*n*-hexane (0–20% EtOAc). The fractions were combined, evaporated, and dried *in vacuo*. Yield: 149 mg of yellow powder (0.58 mmol, 32%). ^1^H-NMR (500.10 MHz, CDCl_3_) δ 8.11 (dd, *J* = 7.7, 1.3 Hz, 1H), 8.06 (dd, *J* = 7.6, 1.3 Hz, 1H), 7.75 (ddd, *J* = 7.6, 7.6, 1.4 Hz, 1H), 7.67 (ddd, *J* = 7.5, 7.5, 1.3 Hz, 1H) 7.42 (s, 1H), 3.12–3.03 (m, 1H), 2.04–1.91 (m, 2H), 1.85–1.77 (m, 2H), 1.76–1.69 (m, 1H), 1.61–1.58 (m, 2H), 1.46–1.25 (m, 3H). Anal. Calcd for C_16_H_16_O_3_: C, 74.98%; H, 6.29%; O, 18.73%. Found: C, 74.64%; H, 6.29%; O, 18.45%.

#### 2-Hydroxy-3-(3-methylbut-2-en-1-yl)naphthalene-1,4-dione (**5**).^41^

**1** (390 mg, 2.24 mmol) and 3,3-dimethylallyl bromide (1314 μL, 1669 mg, 11.20 mmol) were dissolved in 60 mL of 1,4-dioxane. Pd(Ph_3_)_4_ (254 mg, 0.22 mmol) and triethylamine (937 μL, 680 mg, 6.72 mmol) were added. The mixture was stirred at room temperature for 4 h. Afterward, the mixture was poured into aqueous HCl (3 M) and extracted with CHCl_3_. The organic layer was washed with brine, dried over sodium sulfate, and purified by flash column chromatography (SiO_2_) with CHCl_3_/*n*-hexane (40–50% CHCl_3_). The fractions were combined, evaporated, and dried *in vacuo*. Yield: 125 mg of yellow powder (0.52 mmol, 23%). ^1^H-NMR (500.10 MHz, CDCl_3_) δ 8.12 (dd, *J* = 7.8, 1.3 Hz, 1H), 8.07 (dd, *J* = 7.6, 1.3 Hz, 1H), 7.75 (ddd, *J* = 7.6, 7.6, 1.4 Hz, 1H), 7.67 (ddd, *J* = 7.6, 7.5, 1.3 Hz, 1H), 7.31 (s, 1H), 5.23–5.18 (m, 1H), 3.31 (d, *J* = 7.3 Hz, 2H), 1.74 (s, 3H), 1.68 (s, 3H). Elemental analysis found: C, 74.08; H, 5.76; O, 20.01. Calcd for C_15_H_14_O_3_: C, 74.36; H, 5.82; O, 19.81%.

#### 2-Chloro-3-hydroxynaphthalene-1,4-dione (**6**).^42^

Sodium hydroxide (440 mg, 11.00 mmol) was dissolved in 22 mL of water, and 2,3-dichloro-1,4-naphthoquinone (1000 mg, 4.40 mmol) was added slowly. The mixture was stirred for 2 h at 45 °C, and after getting to room temperature, the mixture was acidified with conc. HCl. The yellow precipitate was separated and recrystallized from ethanol (25 mL). The crystalline solid was separated and dried *in vacuo*. Yield: 548 mg of yellow crystals (2.63 mmol, 60%). ^1^H-NMR (500.10 MHz, CDCl_3_) δ 8.21 (dd, *J* = 7.6, 1.3 Hz, 1H), 8.14 (dd, *J* = 7.6, 1.4 Hz, 1H), 7.82 (ddd, *J* = 7.6, 7.6, 1.4 Hz, 1H), 7.76 (ddd, *J* = 7.6, 7.6, 1.4 Hz, 1H), 7.67 (s, 1H). Elemental analysis found: C, 57.93; H, 2.42; O, 23.01. Calcd for C_10_H_5_ClO_3_: C, 57.59; H, 2.42; O, 23.00%.

#### 2-Bromo-3-hydroxynaphthalene-1,4-dione (**7**).^43^

2,3-Dibromo-1,4-naphthoquinone (500 mg, 1.58 mmol) was suspended in 10 mL of H_2_O. Potassium hydroxide (247 mg, 4.40 mmol) was dissolved in 10 mL of H_2_O and added dropwise to the yellow suspension. The dark red mixture was stirred for 3 h at 70 °C. Afterward, the aqueous solution was washed three times with dichloromethane and acidified with HCl (37%). The yellow solid was separated and dried *in vacuo* at 70 °C. Yield: 270 mg of yellow solid (1.07 mmol, 68%). ^1^H-NMR (500.10 MHz, CDCl_3_) δ 8.22 (dd, *J* = 7.7, 1.3 Hz, 1H), 8.15 (dd, *J* = 7.7, 1.2 Hz, 1H), 7.81 (ddd, *J* = 7.6, 7.6, 1.4 Hz, 1H), 7.78 (s, 1H), 7.76 (ddd, *J* = 7.5, 7.5, 1.4 Hz, 1H). Elemental analysis found: C, 47.23; H, 1.94; O, 19.10. Calcd for C_10_H_5_BrO_3_: C, 47.46; H, 1.99; O, 18.97%.

#### 2-Hydroxy-3-(morpholinomethyl)naphthalene-1,4-dione (**8**).^44^

**1** (388 mg, 2.23 mmol) was suspended in 30 mL of ethanol. Morpholine (203.8 μL, 204 mg, 2.34 mmol) and formaldehyde solution (37–41%) (232.8 μL, 70 mg, 2.34 mmol) were added to the suspension and stirred for 4 h at room temperature. The dark red mixture was stored in the fridge overnight for complete precipitation. Afterward, the solid was separated, washed once with ice cold water, and dried *in vacuo*. Yield: 530 mg of red powder (1.94 mmol, 87%). ^1^H-NMR (500.10 MHz, DMSO-*d*_6_) δ 7.95 (dd, *J* = 7.7, 1.2 Hz, 1H), 7.83 (dd, *J* = 7.6, 1.3 Hz, 1H), 7.71 (ddd, *J* = 7.5, 7.5, 1.4 Hz, 1H), 7.58 (ddd, *J* = 7.5, 7.5, 1.3 Hz, 1H), 4.08 (s, 2H), 3.78 (s, 4H), 3.16 (s, 4H). Elemental analysis found: C, 65.63; H, 6.29; N, 5.17; O, 23.49. Calcd for C_15_H_15_NO_4_: C, 65.92; H, 5.53; N, 5.13; O, 23.42%.

#### 2-Hydroxy-3-(thiomorpholinomethyl)naphthalene-1,4-dione (**9**).^44^

**1** (500 mg, 2.87 mmol) was suspended in 30 mL of ethanol. Thiomorpholine (303 μL, 311 mg, 3.02 mmol) and formaldehyde solution (37–41%) (227.0 μL, 91 mg, 3.02 mmol) were added to the suspension and stirred for 4 h at room temperature. The dark red mixture was stored in the fridge overnight for complete precipitation. Afterward, the solid was separated, washed once with ice cold water, and dried *in vacuo*. Yield: 803 mg of red powder (2.78 mmol, 97%). ^1^H-NMR (500.10 MHz, DMSO-*d*_6_) δ 7.95 (dd, *J* = 7.6, 1.3 Hz, 1H), 7.84 (d, *J* = 7.6 Hz, 1H), 7.71 (ddd, *J* = 7.5, 7.5, 1.4 Hz, 1H), 7.59 (ddd, *J* = 7.5, 7.5, 1.3 Hz, 1H), 4.07 (s, 2H), 2.89 (t, *J* = 5.2 Hz, 4H), 2.51 (signal under solvent peak, 4H). Elemental analysis found: C, 61.59; H, 5.24; N, 4.84; S, 10.76; O, 17.39. Calcd for C_15_H_15_O_3_S(H_2_O)_0.2_: C, 61.50; H, 5.30; N, 4.78; S, 10.94; O, 17.48%.

### General Procedure for Complex Synthesis (**1a**–**9a**)

[RuCl_2_(*p*-cymene)]_2_ (0.10 mmol), 1*H*-pyrazole (0.19–0.21 mmol), NEt_3_ (0.6 mmol), and the desired naphthoquinones (**1**–**9**) (0.19–0.21 mmol) were dissolved in 8–12 mL of methanol and stirred at 50–60 °C for 12–15 min under microwave irradiation. The solvent was removed, and the residue was purified by flash chromatography (SiO_2_) with a ternary eluent system (EtOAc/*n*-hexane/NEt_3_ or EtOAc/MeOH/NH_4_OH). The fractions were combined, evaporated, and dried *in vacuo*. Oily residues were dissolved in dichloromethane, precipitated with *n*-hexane, separately washed with *n*-hexane, and dried *in vacuo*.^29^

#### [4-Oxo-1-(1*H*-κ*N*^2^-pyrazol-1-yl)-1,4-dihydronaphtalene-1,2-bis(olato)-κ*O*^1^-κ*O*^2^)(η^6^-*p*-cymene)ruthenium(II)] (**1a**)

The reaction was performed according to the general procedure using [RuCl_2_(*p*-cymene)]_2_ (150 mg, 0.25 mmol), 1*H*-pyrazole (35 mg, 0.52 mmol), **1** (132 mg, 0.76 mmol), and NEt_3_ (342 μL, 248 mg, 2.45 mmol). The mixture was stirred at 50 °C for 15 min under microwave irradiation. Flash chromatography with EtOAc/MeOH/NH_4_OH (88/10/2). Yield: 186 mg yellow/orange powder (0.39 mmol, 78%). ^1^H-NMR (600.25 MHz, MeOD-*d*_4_) δ 8.36 (d, *J* = 2.2 Hz, 1H, H3′), 8.11–8.07 (m, 1H, Harom.), 7.66–7.60 (m, 3H, Harom.), 6.72 (d, *J* = 2.6 Hz, 1H, H5′), 6.37 (dd, *J* = 2.4, 2.4 Hz, 1H, H5′), 5.97 (d, *J* = 5.9 Hz, 1H, Hc), 5.90 (d, *J* = 5.9 Hz, 1H, Hc), 5.64 (d, *J* = 5.9 Hz, 1H, Hb), 5.59 (d, *J* = 5.9 Hz, 1H, Hb), 5.30 (s, 1H, H4), 2.86 (hept, *J* = 6.8 Hz, 1H, He), 2.32 (s, 3H, Hg), 1.32 (d, *J* = 6.9 Hz, 6H, Hf). ^13^C-NMR (150.95 MHz, MeOD-*d*_4_) δ 187.5 (C2), 185.5 (C4), 141.8 (C3′), 138.2 (Carom.), 133.9 (Carom.), 132.9 (CHarom.), 131.3 (CHarom.), 128.2 (C5′), 128.1 (CHarom.), 127.0 (CHarom.), 109.0 (C4′), 101.0 (Ca), 99.0 (Cd), 98.1 (C3), 95.3 (C1), 83.5 (Cc), 83.1 (Cc), 80.1 (Cb), 80.0 (Cb), 32.6 (Ce), 23.0 (Cf), 22.9 (Cf), 18.4 (Cg). Elemental analysis found: C, 57.08; H, 4.57; N, 5.88; O, 10.78. Calcd for C_23_H_22_N_2_O_3_Ru(H_2_O)_0.4_: C, 57.23; H, 4.76; N, 5.80; O, 11.27%.

#### [3-Methyl-4-oxo-(1*H*-κ*N*^2^-pyrazol-1-yl)-1,4-dihydronaphtalene-1,2-bis(olato)-κ*O*^1^-κ*O*^2^)(η^6^-*p*-cymene)ruthenium(II)] (**2a**)

The reaction was performed according to the general procedure using [RuCl_2_(*p*-cymene)]_2_ (150 mg, 0.24 mmol), 1*H*-pyrazole (34 mg, 0.50 mmol), **2** (94 mg, 0.50 mmol), and NEt_3_ (201 μL, 146 mg, 1.44 mmol). The mixture was stirred at 60 °C for 15 min under microwave irradiation. Flash chromatography with EtOAc/*n*-hexane/NEt_3_ (85/10/5). Yield: 180 mg of yellow solid (0.37 mmol, 77%). ^1^H-NMR (500.10 MHz, DMSO-*d*_6_) δ 8.41 (d, *J* = 2.1 Hz, 1H, H3′), 7.96–7.90 (m, 1H, Harom.), 7.55–7.49 (m, 2H, Harom.), 7.49–7.43 (m, 1H, Harom.), 6.67 (d, *J* = 2.5 Hz, 1H, H5′), 6.31 (dd, *J* = 2.3, 2.3 Hz, 1H, H4′), 6.05 (d, *J* = 5.8 Hz, 1H, Hc), 5.98 (d, *J* = 5.8 Hz, 1H, Hc), 5.67 (d, *J* = 5.9 Hz, 1H, Hb), 5.59 (d, *J* = 5.8 Hz, 1H, Hb), 2.77 (hept, *J* = 6.9 Hz, 1H, He), 2.25 (s, 3H, Hg), 1.59 (s, 3H, H9), 1.24 (dd, *J* = 7.0, 1.6 Hz, 6H, Hf). ^13^C-NMR (150.95 MHz, MeOD-*d*_4_) δ 183.9 (C2), 183.6 (C4), 141.5 (C3‘), 137.8 (Carom.), 134.1 (Carom.), 132.3 (Carom.), 131.2 (Carom.), 127.9 (C5′), 127.7 (Carom.), 127.1 (Carom.), 108.7 (C4′), 105.6 (C3), 101.0 (Cd), 98.7 (Ca), 94.8 (C1), 83.3 (Cc), 83.1 (Cc), 80.2 (Cb), 80.1 (Cb), 32.7 (Ce), 23.0 (Cf), 22.8 (Cf), 18.3 (Cg), 8.1 (C9). Elemental analysis found: C, 58.19; H, 4.95; N, 5.84; O, 10.05. Calcd for C_24_H_24_N_2_O_3_Ru(H_2_O)_0.2_: C, 58.45; H, 4.99; N, 5.68; O, 10.38%.

#### [3-Ethyl-4-oxo-(1*H*-κ*N*^2^-pyrazol-1-yl)-1,4-dihydronaphtalene-1,2-bis(olato)-κ*O*^1^-κ*O*^2^)(η^6^-*p*-cymene)ruthenium(II)] (**3a**)

The reaction was performed according to the general procedure using [RuCl_2_(*p*-cymene)]_2_ (120 mg, 0.20 mmol), 1*H*-pyrazole (25 mg, 0.37 mmol), **3** (75 mg, 0.37 mmol), and NEt_3_ (164 μL, 119 mg, 1.18 mmol). The mixture was stirred at 50 °C for 15 min under microwave irradiation. Flash chromatography with EtOAc/*n*-hexane/NEt_3_ (85/10/5). Yield: 106 mg of yellow solid (0.21 mmol, 57%). ^1^H-NMR (600.25 MHz, MeOD-*d*_4_) δ 8.33 (d, *J* = 2.8 Hz, 1H, H3′), 8.11–8.07 (m, 1H, Harom.), 7.62–7.57 (m, 3H, Harom.), 6.68 (d, *J* = 2.4 Hz, 1H, H5′), 6.34 (dd, *J* = 2.4, 2.4 Hz, 1H, H4′), 5.97 (d, *J* = 6.5 Hz, 1H, Hc), 5.87 (d, *J* = 6.3 Hz, 1H, Hc), 5.62–5.59 (m, 2H, Hb), 2.87 (hept, *J* = 6.9 Hz, 1H, He), 2.41–2.34 (m, 1H, H9), 2.33 (s, 3H, Hg), 2.32–2.25 (m, 1H, H9), 1.33 (d, *J* = 6.9 Hz, 6H, Hf), 0.88 (t, *J* = 7.4 Hz, 3H, H10). ^13^C-NMR (150.95 MHz, MeOD-*d*_4_) δ 183.5 (C2), 183.1 (C4), 141.4 (C3′), 137.7 (Carom.), 134.4 (Carom.), 132.2 (Carom.), 131.2 (Carom.), 127.9 (Carom.), 127.7 (Carom.), 127.1 (C5′), 112.3 (C3), 108.7 (C4′), 101.3 (Cd), 98.6 (Ca), 94.9 (C1), 83.3 (Cc), 82.9 (Cc), 80.2 (Cb), 80.1 (Cb), 32.7 (Ce), 23.1 (Cf), 22.8 (Cf), 18.3 (Cg), 16.9 (C9), 13.7 (C10). Elemental analysis found: C, 58.83; H, 5.17; N, 5.47; O, 10.20. Calcd for C_25_H_26_N_2_O_3_Ru(H_2_O)_0.2_: C, 59.21; H, 5.25; N, 5.52; O, 10.10%.

#### [3-Cyclohexyl-4-oxo-(1*H*-κ*N*^2^-pyrazol-1-yl)-1,4-dihydronaphtalene-1,2-bis(olato)-κ*O*^1^-κ*O*^2^)(η^6^-*p*-cymene)ruthenium(II)] (**4a**)

The reaction was performed according to the general procedure using [RuCl_2_(*p*-cymene)]_2_ (55 mg, 0.09 mmol), 1*H*-pyrazole (13 mg, 0.19 mmol), **4** (49 mg, 0.19 mmol), and NEt_3_ (75 μL, 55 mg, 0.54 mmol). The mixture was stirred at 60 °C for 15 min under microwave irradiation. Flash chromatography with EtOAc/*n*-hexane/NEt_3_ (70/25/5). Yield: 57 mg of yellow/greenish solid (0.10 mmol, 56%). ^1^H-NMR (600.10 MHz, DMSO-*d*_6_) δ 8.40 (dd, *J* = 2.1, 0.7 Hz, 1H, H3′), 7.95–7.89 (m, 1H, Harom.), 7.53–7.47 (m, 2H, Harom.), 7.46–7.42 (m, 1H, Harom.), 6.66 (dd, *J* = 2.5, 0.7 Hz, 1H, H5′), 6.29 (dd, *J* = 2.3, 2.3 Hz, 1H, H4′), 6.03 (d, *J* = 5.9 Hz, 1H, Hc), 5.96 (d, *J* = 5.9 Hz, 1H, Hc), 5.67 (d, *J* = 5.9 Hz, 1H, Hb), 5.59 (d, *J* = 5.8 Hz, 1H, Hb), 2.81–2.73 (m, 2H, He and H9), 2.25 (s, 3H, Hg), 2.07–1.96 (m, 1H, Hhexyl), 1.85–1.75 (m, 1H, Hhexyl), 1.69–1.57 (m, 3H, Hhexyl), 1.27 (d, *J* = 2.4 Hz, 3H, Hf), 1.25 (d, *J* = 2.4 Hz, 3H, Hf), 1.19–1.09 (m, 3H, Hhexyl), 1.05–0.98 (m, 1H, Hhexyl). ^13^C-NMR (150.95 MHz, DMSO-*d*_6_) δ 181.0 (C2), 179.4 (C4), 139.6 (C3′), 136.7 (Carom.), 132.9 (Carom.), 130.2 (CHarom.), 129.2 (CHarom.), 126.1 (CHarom.), 125.9 (C5′), 125.5 (CHarom.), 111.8 (C3), 107.2 (C4′), 97.9 (Cd), 96.5 (Ca), 95.4 (C1), 82.1 (Cc), 81.8 (Cc), 78.3 (Cb), 77.6 (Cb), 33.6 (Ce), 30.8 (C9), 30.0 (Chexyl), 29.4 (Chexyl), 27.1 (Chexyl), 27.0 (Chexyl), 26.1 (Chexyl), 22.8 (Cf), 22.2 (Cf), 17.8 (Cg). Elemental analysis found: C, 61.59; H, 5,77; N, 5.01; O, 9.09. Calcd for C_29_H_32_N_2_O_3_Ru(H_2_O)_0.25_: C, 61.85; H, 5.82; N, 4.98; O, 9.24%.

#### [3-(3-methylbut-2-en-1-yl)-4-oxo-(1*H*-κ*N*^2^-pyrazol-1-yl)-1,4-dihydronaphtalene-1,2-bis(olato)-κ*O*^1^-κ*O*^2^)(η^6^-*p*-cymene)ruthenium(II)] (**5a**)

The reaction was performed according to the general procedure using [RuCl_2_(*p*-cymene)]_2_ (150 mg, 0.24 mmol), 1*H*-pyrazole (31 mg, 0.46 mmol), **5** (111 mg, 0.46 mmol), and NEt_3_ (201 μL, 146 mg, 1.44 mmol). The mixture was stirred at 50 °C for 15 min under microwave irradiation. Flash chromatography with EtOAc/*n*-hexane/NEt_3_ (70/28/2). Yield: 142 mg of yellow solid (0.26 mmol, 57%). ^1^H-NMR (600.25 MHz, DMSO-*d*_6_) δ 8.41 (d, *J* = 1.5 Hz, 1H, H3′), 7.96–7.90 (m, 1H, Harom.), 7.55–7.49 (m, 2H, Harom.), 7.48–7.43 (m, 1H, Harom.), 6.66 (d, *J* = 2.4 Hz, 1H, H5′), 6.31 (dd, *J* = 2.3, 2.3 Hz, 1H, H4′), 6.03 (d, *J* = 5.8 Hz, 1H, Hc), 5.96 (d, *J* = 5.9 Hz, 1H, Hc), 5.65 (d, *J* = 5.9 Hz, 1H, Hb), 5.58 (d, *J* = 5.8 Hz, 1H, Hb), 5.07–5.02 (m, 1H, H10), 2.91–2.86 (m, 1H, H9), 2.82–2.72 (m, 2H, He and H9), 2.24 (s, 3H, Hg), 1.58 (s, 6H, H12), 1.54 (s, 3H, H12) 1.24 (d, *J* = 1.4 Hz, 3H, Hf), 1.23 (d, *J* = 1.5 Hz, 3H, Hf). ^13^C-NMR (150.95 MHz, DMSO-*d*_6_) δ 180.5 (C2), 179.6 (C4), 139.7 (C3′), 136.8 (Carom.), 132.7 (Carom.), 130.4 (CHarom.), 129.3 (CHarom.), 128.3 (C11), 126.3 (CHarom.), 126.1 (C5′), 125.3 (CHarom.), 124.4 (C10), 107.2 (C4′), 106.6 (C3), 97.8 (Cd), 96.9 (Ca), 95.3 (C1), 82.2 (Cc), 81.8 (Cc), 78.1 (Cb), 77.6 (Cb), 30.8 (Ce), 25.5 (C12), 22.7 (Cf), 22.2 (Cf), 21.8 (C9), 17.7 (Cg), 17.6 (C12). Elemental analysis found: C, 61.11; H, 5.48; N, 5.24; O, 9.03. Calcd for C_28_H_30_N_2_O_3_Ru(H_2_O)_0.2_: C, 61.46; H, 5.60; N, 5.11; O, 9.36%.

#### [3-Chloro-4-oxo-(1*H*-κ*N*^2^-pyrazol-1-yl)-1,4-dihydronaphtalene-1,2-bis(olato)-κ*O*^1^-κ*O*^2^)(η^6^-*p*-cymene)ruthenium(II)] (**6a**)

The reaction was performed according to the general procedure using [RuCl_2_(*p*-cymene)]_2_ (77 mg, 0.13 mmol), 1*H*-pyrazole (16 mg, 0.24 mmol), **6** (50 mg, 0.24 mmol), and NEt_3_ (106 μL, 77 mg, 0.76 mmol). The mixture was stirred at 60 °C for 15 min under microwave irradiation. Flash chromatography with EtOAc/*n*-hexane/NEt_3_ (90/5/5). Yield: 77 mg of yellow/orange powder (0.15 mmol, 63%). ^1^H-NMR (600.25 MHz, MeOD-*d*_4_) δ 8.40 (d, *J* = 2.1 Hz, 1H, H3′), 8.16–8.12 (m, 1H, Harom.), 7.68–7.60 (m, 3H, Harom.), 6.74 (d, *J* = 2.6 Hz, 1H, H5′), 6.38 (dd, *J* = 2.4, 2.4 Hz, 1H, H4′), 6.03 (d, *J* = 5.9 Hz, 1H, Hc), 5.93 (d, *J* = 5.9 Hz, 1H, Hc), 5.68 (d, *J* = 6.0 Hz, 1H, Hb), 5.66 (d, *J* = 6.0 Hz, 1H, Hb), 2.89 (hept, *J* = 6.9 Hz, 1H, He), 2.35 (s, 3H, Hg), 1.34 (d, *J* = 1.7 Hz, 3H, Hf), 1.33 (d, *J* = 1.7 Hz, 3H, Hf). ^13^C-NMR (150.95 MHz, MeOD-*d*_4_) δ 180.8 (C2), 178.6 (C4), 142.0 (C3′), 137.0 (Carom.), 133.3 (Carom.), 133.1 (Carom.), 131.7 (Carom.), 128.4 (C5′), 128.1 (Carom.), 127.6 (Carom.), 109.2 (C4′), 104.1 (C3), 101.3 (Ca), 99.0 (Cd), 96.7 (C1), 83.4 (Cc), 83.1 (Cc), 80.4 (Cb), 80.3 (Cb), 32.7 (Ce), 23.0 (Cf), 22.8 (Cf), 18.4 (Cg). Elemental analysis found: C, 53.87; H, 4.28; N, 5.49; O, 9.65. Calcd for C_23_H_21_ClN_2_O_3_Ru: C, 54.17; H, 4.15; N, 5.49; O, 9.41%.

#### [3-Bromo-4-oxo-(1*H*-κ*N*^2^-pyrazol-1-yl)-1,4-dihydronaphtalene-1,2-bis(olato)-κ*O*^1^-κ*O*^2^)(η^6^-*p*-cymene)ruthenium(II)] (**7a**)

The reaction was performed according to the general procedure using [RuCl_2_(*p*-cymene)]_2_ (150 mg, 0.24 mmol), 1*H*-pyrazole (32 mg, 0.47 mmol), **7** (118 mg, 0.47 mmol), and NEt_3_ (205 μL, 149 mg, 1.47 mmol). The mixture was stirred at 50 °C for 15 min under microwave irradiation. Flash chromatography with EtOAc/*n*-hexane/NEt_3_ (90/5/5). Yield: 126 mg of yellow solid (0.23 mmol, 49%). ^1^H-NMR (600.25 MHz, DMSO-*d*_6_) δ 8.51 (d, *J* = 1.5 Hz, 1H, H3′), 8.00–7.96 (m, 1H, Harom.), 7.64–7.56 (m, 2H, Harom.), 7.51–7.48 (m, 1H, Harom.), 6.80 (d, *J* = 2.2 Hz, 1H, H5′), 6.37 (dd, *J* = 2.3, 2.3 Hz, 1H, H4′), 6.12 (d, *J* = 5.9 Hz, 1H, Hc), 6.06 (d, *J* = 5.9 Hz, 1H, Hc), 5.76 (d, *J* = 5.9 Hz, 1H, Hb), 5.69 (d, *J* = 5.9 Hz, 1H, Hb), 2.79 (hept, *J* = 6.9 Hz, 1H, He), 2.26 (s, 3H, Hg), 1.26 (d, *J* = 2.5 Hz, 3H, Hf), 1.25 (d, *J* = 2.5 Hz, 3H, Hf). ^13^C-NMR (150.95 MHz, DMSO-*d*_6_) δ 180.1 (C2), 175.2 (C4), 140.4 (C3′), 136.0 (Carom.), 131.4 (Carom.), 131.4 (CHarom.), 129.9 (CHarom.), 126.8 (C5′), 126.7 (CHarom.), 125.9 (CHarom.), 107.8 (C4′), 98.2 (Ca), 97.2 (Cd), 97.0 (C3), 92.4 (C1), 82.1 (Cc), 81.9 (Cc), 78.5 (Cb), 77.9 (Cb), 30.8 (Ce), 22.6 (Cf), 22.2 (Cf), 17.6 (Cg). Elemental analysis found: C, 49.44; H, 3.94; N, 4.82; O, 9.66. Calcd for C_23_H_21_N_2_BrO_3_Ru(H_2_O)_0.3_: C, 49.35; H, 3.89; N, 5.00; O, 9.43%.

#### [3-(Morpholinomethyl)-4-oxo-(1*H*-κ*N*^2^-pyrazol-1-yl)-1,4-dihydronaphtalene-1,2-bis(olato)-κ*O*^1^-κ*O*^2^)(η^6^-*p*-cymene)ruthenium(II)] (**8a**)

The reaction was performed according to the general procedure using [RuCl_2_(*p*-cymene)]_2_ (150 mg, 0.24 mmol), 1*H*-pyrazole (35 mg, 0.51 mmol), **8** (150 mg, 0.51 mmol), and NEt_3_ (201 μL, 146 mg, 1.44 mmol). The mixture was stirred at 50 °C for 15 min under microwave irradiation. Flash chromatography with EtOAc/MeOH/NH_4_OH (88/10/2). Yield: 152 mg of yellow solid (0.26 mmol, 54%). ^1^H-NMR (600.25 MHz, MeOD-*d*_4_) δ 8.35 (d, *J* = 1.5 Hz, 1H, H3′), 8.14–8.10 (m, 1H, Harom.), 7.67–7.60 (m, 3H, Harom.), 6.72 (dd, *J* = 2.6, 0.6 Hz, 1H, H5′), 6.35 (dd, *J* = 2.4, 2.4 Hz, 1H, H4′), 5.99 (d, *J* = 5.8 Hz, 1H, Hc), 5.92 (d, *J* = 6.0 Hz, 1H, Hc), 5.64 (d, *J* = 5.9 Hz, 1H, Hb), 5.58 (d, *J* = 5.9 Hz, 1H, Hb), 3.55 (t, *J* = 4.8 Hz, 4H, H11), 3.50 (d, *J* = 12.4 Hz, 1H, H9), 3.41 (d, *J* = 12.4 Hz, 1H, H9), 2.86 (hept, *J* = 6.9 Hz, 1H, He), 2.42–2.35 (m, 2H, H10), 2.33 (s, 3H, Hg), 2.27–2.19 (m, 2H, H10), 1.34 (d, *J* = 2.1 Hz, 3H, Hf), 1.33 (d, *J* = 2.2 Hz, 3H, Hf). ^13^C-NMR (150.95 MHz, MeOD-*d*_4_) δ 186.3 (C2), 183.7 (C4), 141.6 (C3′), 137.6 (Carom.), 134.0 (Carom.), 132.6 (CHarom.), 131.3 (CHarom.), 128.1 (C5′), 127.8 (CHarom.), 127.4 (CHarom.), 109.0 (C4′), 104.3 (C3), 100.8 (Cd), 98.8 (Ca), 95.0 (C1), 83.8 (Cc), 83.1 (Cc), 80.1 (Cb), 78.0 (Cb), 67.4 (C11), 54.0 (C10), 50.6 (C9), 32.6 (Cg), 23.2 (Cf), 22.8 (Cf), 18.5 (Cg). Elemental analysis found: C, 58.35; H, 5.48; N, 7.32; O, 11.22. Calcd for C_28_H_31_N_3_O_4_Ru: C, 58.52; H, 5.43; N, 7.31; O, 11.14%.

#### [4-Oxo-1-(1*H*-κ*N*^2^-pyrazol-1-yl)-3-(thiomorpholinomethyl)-1,4-dihydronaphtalene-1,2-bis(olato)-κ*O*^1^-κ*O*^2^)(η^6^-*p*-cymene)ruthenium(II)] (**9a**)

The reaction was performed according to the general procedure using [RuCl_2_(*p*-cymene)]_2_ (100 mg, 0.16 mmol), 1*H*-pyrazole (23 mg, 0.34 mmol), **9** (99 mg, 0.34 mmol), and NEt_3_ (134 μL, 97 mg, 0.96 mmol). The mixture was stirred at 50 °C for 15 min under microwave irradiation. Flash chromatography with EtOAc/MeOH/NH_4_OH (88/10/2). Yield: 62 mg of yellow solid (0.11 mmol, 34%). ^1^H-NMR (600.25 MHz, MeOD-*d*_4_) δ 8.35 (d, *J* = 1.6 Hz, 1H, H3′), 8.13–8.10 (m, 1H, Harom.), 7.66–7.60 (m, 3H, Harom.), 6.72 (d, *J* = 2.6 Hz, 1H, H5′), 6.35 (dd, *J* = 2.4, 2.4 Hz, 1H, H4′), 5.99 (d, *J* = 5.8 Hz, 1H, Hc), 5.92 (d, *J* = 6.0 Hz, 1H, Hc), 5.64 (d, *J* = 5.9 Hz, 1H, Hb), 5.58 (d, *J* = 5.9 Hz, 1H, Hb), 3.55 (t, *J* = 4.8 Hz, 4H, H10), 3.50 (d, *J* = 12.4 Hz, 1H, H9), 3.41 (d, *J* = 12.4 Hz, 1H, H9), 2.86 (hept, *J* = 6.9 Hz, 1H, He), 2.41–2.35 (m, 2H, H11), 2.33 (s, 3H, Hg), 2.26–2.20 (m, 2H, H11), 1.34 (d, *J* = 2.1 Hz, 3H, Hf), 1.33 (d, *J* = 2.2 Hz, 3H, Hf). ^13^C-NMR (150.95 MHz, MeOD-*d*_4_) δ 186.3 (C2), 183.7 (C4), 141.6 (C3′), 137.6 (Carom.), 134.0 (Carom.), 132.6 (CHarom.), 131.3 (CHarom.), 128.1 (C5′), 127.8 (CHarom.), 127.4 (CHarom.), 109.0 (C4′), 104.3 (C3), 100.8 (Ca), 98.8 (Cd), 95.0 (C1), 83.8 (Cc), 83.1 (Cc), 80.1 (Cb), 80.0 (Cb), 67.4 (C10), 54.0 (C11), 50.6 (C9), 32.6 (Ce), 23.2 (Cf), 22.8 (Cf), 18.5 (Cg). Elemental analysis found: C, 55.60; H, 5.29; N, 6.94; S, 5.14; O, 9.37. Calcd for C_28_H_31_N_3_O_3_RuS(H_2_O)_0.5_: C, 55.98; H, 5.54; N, 6.99; S, 5.34; O, 9.32%.

### Theoretical Simulations

Optimizations of energetic minima and maxima (transition states) were carried out with the ORCA program suite^45^ and the PBEh-3c method.^46^ The PBEh-3c method is a new method based on density functional theory (DFT) that uses the PBE0 functional, reparametrized with 42% Hartree–Fock exchange, and the def2-mSVP double-ζ basis set. It further accounts for dispersion correction and the basis set superposition error and includes the ZORA effective core potential for the Ru atom. This method has been shown to be more reliable than most frequently applied DFT protocols, such as B3LYP/6-31G*, and turned out to be most suitable for the purpose of this study.^45,46^ A tight convergence was set for the self-consistent field, and the DFT grid was set to 4. The conductor-like polarizable continuum model^47^ was used to model the solvent with a dielectric constant of 80.4 and a refractive index of 1.33. Due to numerical instabilities and to obtain smoother potentials, a Gaussian smearing^48^ was applied to the point charge. After every converged optimization, a frequency calculation was carried out to confirm a minimum or transition state on the potential energy landscape. For every geometry except for complex **8a**, we used X-ray structures as initial guesses for geometry optimizations. The structure of complex **8a** was estimated from structure **9a** after substituting the sulfur atom with an oxygen atom.

The binding energy (BE) of each complex was computed according to ref (51), where the BE is estimated as the difference of the sum of the thermal energies of the isolated complex (RuX) and the isolated water (H_2_O) in the solvent and the aqua complex (RuXH_2_O) with a water molecule coordinated to the metal center:
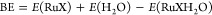
1

The structure of the aqua complex was estimated by elongation of the bond of Ru–*O*2 and by placing a water molecule close to the Ru atom. The initial structures were preoptimized at the semiempirical HF-3c level of theory. The relaxed structure was used as an input for a subsequent optimization with PBEh-3c.

In order to estimate the energy barrier for the aquation process, we carried out a transition state search. The initial guess for the transition state structure was obtained from a nudged elastic band^49,50^ calculation using Turbomole.^51^ This method estimates the minimum energy path between the complex and a free water molecule far away from the Ru atom and the aqua complex. The same level of theory was applied as for geometry optimizations, and default parameters for the nudged elastic band method were selected. The guess for the transition state structure was taken from the nudged elastic band simulation and corresponded to the energetically most unfavored structure of the obtained path. This structure was preoptimized with the transition-state search of Gaussian^52^ and the PBE1PBE DFT functional with D3 dispersion correction, the 6-31G basis set, and the LANDL2DZ effective core potential for the Ru atom. The final optimization was carried out in ORCA with the settings described above and a frequency calculation every fifth optimization step to allow for more reliable convergence toward the transition state. A frequency calculation with a single imaginary frequency confirmed the found transition state.

### UV–vis Measurements

Stock solutions were prepared by dissolving compounds **1a**–**9a** in DMSO (10 mM). 1980 μL of PBS buffer (pH 7.4), 12 μL of DMSO, and 8 μL of stock solution were mixed to obtain a final concentration of 40 μM. Immediately, UV–vis spectra were recorded hourly over 48 h with a PerkinElmer lambda 35 photometer with PTP (Peltier Temperature Programmer) and Julabo AWC 100 recirculating cooler.

### HPLC-MS Stability and Amino Acid Incubation Studies

Complex (**1a**–**9a**) stock solutions (10 mM) and samples were prepared as mentioned before. After injection, the chromatogram at 225 nm and the corresponding mass spectra (positive mode) were recorded hourly for 4 h and after 24 h at 20 °C. Peak areas were determined by integration of complex signals of the respective chromatogram. The amino acid incubation studies were performed with PBS buffer solution and additionally contained *N*-Ac-Met-OMe, *N*-Ac-His-OMe, and *N*-Ac-Cys-OMe (400 μM each) at 37 °C. The measurements were conducted with a HPLC system (Agilent Technologies, 126 Infinity) equipped with a C18 column (Waters, Atlantis T3 3 μM, 1.0 × 150 mm^2^) coupled to a MS (Bruker, amaZon SL, ESI, positive mode) with a flow rate of 0.2 mL/min at 20 °C and a gradient with Milli-Q water/ACN.

### MTT Assay

The cytotoxicity of the compounds was determined by using the colorimetric MTT assay (MTT = 3-(4,5-dimethyl-2-thiazolyl)-2,5-diphenyl-2*H*-tetrazolium bromide). 1 × 10^3^ CH1/PA-1, 2 × 10^3^ SW480, and 3 × 10^3^ A549 cells were seeded in 100 μL/well into 96-well microculture plates. After 24 h, test compounds were dissolved in DMSO (Fisher Scientific), serially diluted in complete MEM (to a final DMSO content not exceeding 0.5% v/v), and added in 100 μL/well. After 96 h, the drug-containing medium was replaced with 100 μL of RPMI 1640/MTT mixture [6 parts of RPMI 1640 medium (supplemented with 10% heat-inactivated fetal bovine serum and 4 mM l-glutamine), 1 part of MTT solution in phosphate-buffered saline (5 mg/mL)]. After incubation for 4 h, the MTT-containing medium was replaced with 150 μL of DMSO/well to dissolve the formazan product formed by viable cells. Optical densities at 550 nm (and at a reference wavelength of 690 nm) were measured with a microplate reader (ELx808, Bio-Tek). The 50% inhibitory concentrations (IC_50_) relative to untreated controls were interpolated from concentration–effect curves. At least three independent experiments were performed, each with triplicates per concentration level.

### ROS Assay (DCFH-DA Assay)

Subconfluent cell lines CH1/PA-1 (ovarian teratocarcinoma) and SW480 (colon carcinoma) were trypsinized for 2–5 min in a humidified incubator at 37 °C and under a 5% CO_2_ atmosphere. After addition of supplemented MEM (Sigma-Aldrich; supplements: 10% heat-inactivated FCS (fetal calf serum; BioWest), 1 mM sodium pyruvate, 4 mM l-glutamine, and 1% v/v non-essential amino acid solution), trypsination was stopped and cells were centrifuged for 3 min at 1200 rpm (Thermo Scientific, Megafuge 1.0R). The supernatant was aspirated, and the cell pellet was resuspended in supplemented MEM. Then, both cell lines were seeded in 100 μL aliquots in densities of 2.5 × 10^4^ cells/well in 96-well clear flat-bottom microplates. After 24 h of incubation, cells were washed with 200 μL Hanks’ balanced salt solution (HBSS; supplemented with 1% FCS; Sigma-Aldrich), incubated for 45 min with 100 μL/well of 25 μM 2′,7′-dichlorofluorescin diacetate (DCFH-DA) in HBSS (supplemented with 1% FCS), and washed once more with 200 μL of HBSS (+1% FCS). Afterward, a serial dilution (in phenol-red-free Opti-MEM (Gibco, supplemented with 1% FCS)) of test compound was added in 200 μL triplicates. TBHP (*tert*-butylhydroperoxide) was used as a positive control. Immediately after addition of a compound’s dilution series, fluorescence (ex/em = 480/516 nm) was measured every 10 min for a total period of 2 h with a microplate reader (BioTek, Synergy HT). Obtained values (blank-corrected) were represented in relation to negative controls (incubated with drug-free Opti-MEM) from two independent experiments.

### Plasmid Assay

Stock solutions of the test compounds were prepared in DMSO (Fisher Scientific) and diluted in Milli-Q water. A 400 ng portion of pUC19 dsDNA (2686 bp) plasmid (New England BioLabs) was incubated with 50 μM of the test compounds or cisplatin for different time intervals (15 min to 6 h) at 37 °C under continuous shaking. In addition to the untreated control, a linear pUC19L vector (ThermoFisher Scientific) was used. A 20 μL portion of the samples was added to 4 μL of 6× DNA loading dye (ThermoFisher Scientific) and loaded into the pockets of 1% agarose gel in 1× TBE buffer. Electrophoresis was carried out at 60 V for 5 min, followed by 120 V for 90 min. Ethidium bromide (SERVA) staining was performed in 1× TBE (0.75 μg/mL) for 20 min. Images were taken by the GelDoc-It Imaging System Fusion Fx7 (Vilber Lourmat, Germany). For quantification of the spots, ImageJ/Fiji1.46 was used.

## Results and Discussion

**Scheme 1:**
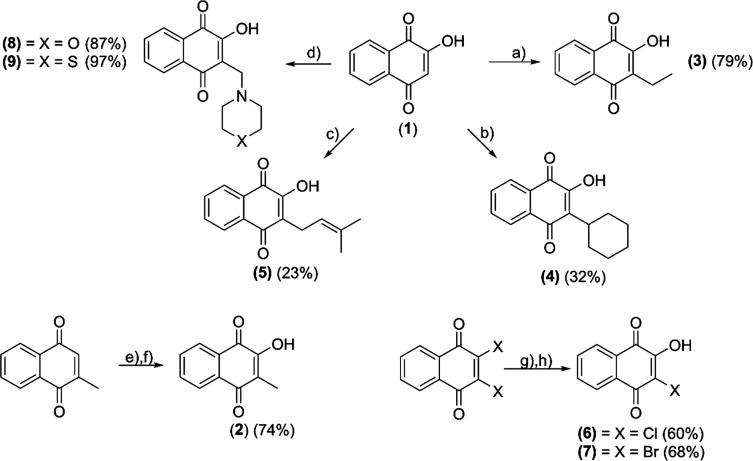
Synthetic Pathway of Hydroxy-1,4-naphthoquinones (**2**–**9**) (a) = acetaldehyde, l-proline, Hantzsch ester, DCM, room temperature (rt), 23 h; (b) = cyclohexene, NaBH_4_, Fe(NO_3_)_3_·9H_2_O; ACN/EtOH, rt, 2 h; (c) = 1-bromo-3-methylbute-2-ene, Pd(Ph_3_)_4_, NEt_3_, 1,4-dioxane, rt, 4 h; (d) = formaldehyde, morpholine/thiomorpholine, EtOH, rt, 4 h; (e) = H_2_O_2_, Na_2_CO_3_, MeOH; 0 °C to rt, 1.5 h; (f) SiO_2_, H_2_SO_4_, THF, 70 °C; (g) = H_2_O, NaOH, rt, 1 h; (h) = H_2_O, KOH, 70 °C, 3 h.

Modifications at position 3 of the naphthoquinone backbone have shown a tremendous impact on the biological properties and were therefore the starting point for this work.^33^ Starting from lawsone (**1**), the desired hydroxy-1,4-naphthoquinones (**2**–**9**) were synthesized according to literature procedures (Scheme 1). Phthiocol (**2**) was synthesized via epoxidation of menadione and subsequent acidic SiO_2_-mediated ring opening (74%).^37,38^ Treatment of lawsone (**1**) with acetaldehyde, l-proline, and diethyl 1,4-dihydro-2,6-dimethyl-3,5-pyridinedicarboxylate (Hantzsch ester) provided compound **3** in good yield (79%).^39^ The Fe^III^-mediated radical alkylation of **1** and NaBH_4_ provided parvaquone (**4**) in moderate yield (32%).^40^ Lapachol (**5**) was synthesized via a Heck reaction using **1**, 3,3-dimethylallyl bromide, NEt_3_, and Pd(Ph_3_)_4_ (23%).^41^ The halogenated derivatives **6** and **7** were obtained by treatment of 2,3-dichloro- or 2,3-dibromo-1,4-naphthoquinone with sodium/potassium hydroxide in yields between 55 and 68%.^42,43^ More water-soluble naphthoquinones were synthesized via a Mannich reaction, where morpholine (**8**) and thiomorpholine (**9**) were employed (87–97%).^44^

**Scheme 2:**
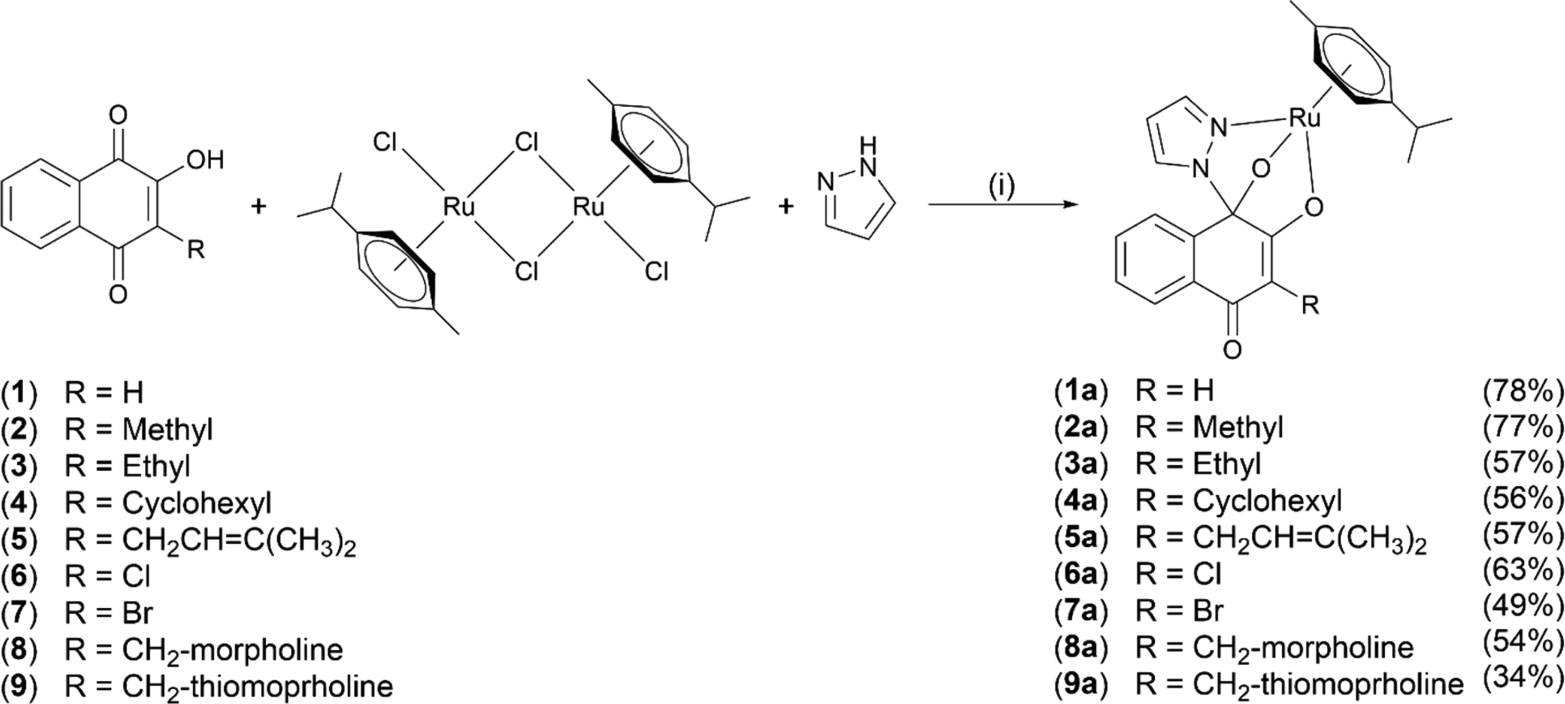
Synthetic Pathway for Complex Syntheses (i) MeOH, microwave, 50–60 °C, 10–15 min.

Complexes **1a**–**9a** were synthesized according to the literature procedure in a one-pot reaction using microwave irradiation (50–60 °C), and subsequent purification via flash column chromatography with a ternary mobile phase (EtOAc/*n*-hexane/NEt_3_ or EtOAc/MeOH/NH_4_OH) (Scheme 2) provided the pure products with yields in the range of 34–78%.^29^

All complexes were characterized by ^1^H and ^13^C NMR spectroscopy and 2D experiments (for spectra, see Figures S1–S26). The purity of the synthesized ligands (**1**–**9**) and complexes (**1a**–**9a**) was determined by elemental analysis.

The formation of the complexes was unambiguously confirmed by the detection of the quaternary carbon C1 around 90–100 ppm in ^13^C-NMR spectra (Figures S13–S26), due to the formation of a hemiaminal functionality. As mentioned in previous studies, these complexes exhibit two stereogenic centers, the metal center and the quaternary carbon at the hemiaminal bond. Therefore, four diastereomers (*R*_C1_, *S*_Ru_; *R*_C1_, *R*_Ru_; *S*_C1_, *R*_Ru_; *S*_C1_, *S*_Ru_) could be generated theoretically.^29^ However, due to sterical demands, only one pair of enantiomers (*R*_C1_, *R*_Ru_ and *S*_C1_, *S*_Ru_) can be formed.

### X-ray Crystallographic Studies

**Figure 3:**
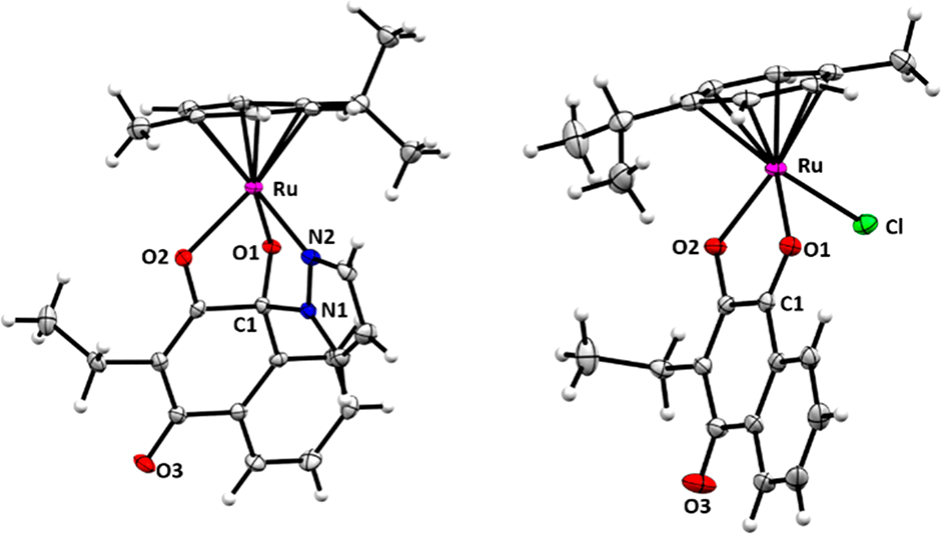
Molecular structures of **3a** and parental complex **III**(33) at 50% probability level. Solvent molecules were omitted for clarity.

Single crystals of eight complexes (**1a**–**7a**, **9a**) were obtained by vapor diffusion or liquid–liquid diffusion from dichloromethane/diethyl ether or dichloromethane/*n*-hexane (Figures S27–S33 and Tables S1–S15). All structures with the exception of **1a** crystallized in monoclinic space groups *C*2/*c* and *P*2_1_/*n*. The crystal structures confirmed the adaption of the characteristic piano-stool geometry, where *p*-cymene represents the seat and the azole coupled naphthoquinone the three legs (Figure 3). Coordinative bond lengths between ruthenium and the ligand’s *O*1 and *N*2 are in the same range (Table 1).

**Table 1:** Selected Bond Lengths (in Å) for Complexes **1a**–**7a**, **9a**, and Their Parental Chlorido Complexes **II**, **III**, and **V** as Obtained from X-ray Crystallography

	**Ru**–***O*****1**	**Ru**–***O*****2**	**Ru**–***N*****2**	**C1**–***N*****1**	**Ru–Cl**
**1a**	2.059(2)	2.121(2)	2.096(3)	1.528(4)	
**2a**(29)	2.061(3)	2.111(3)	2.099(4)	1.517(5)	
**3a**	2.049(2)	2.116(2)	2.097(2)	1.515(3)	
**4a**	2.053(2)	2.114(2)	2.087(2)	1.518(4)	
**5a**	2.047(1)	2.109(2)	2.097(2)	1.501(3)	
**6a**	2.051(2)	2.120(2)	2.097(3)	1.496(4)	
**7a**	2.053(2)	2.119(2)	2.096(3)	1.497(4)	
**9a**	2.047(2)	2.123(2)	2.080(2)	1.516(3)	
**II**(30)	2.119(2)	2.092(2)			2.403(1)
**III**(33)	2.1169(13)	2.0904(13)			2.4022(4)
**V**(32)	2.076(1)	2.107(1)			2.4066(4)

**Figure 4:**
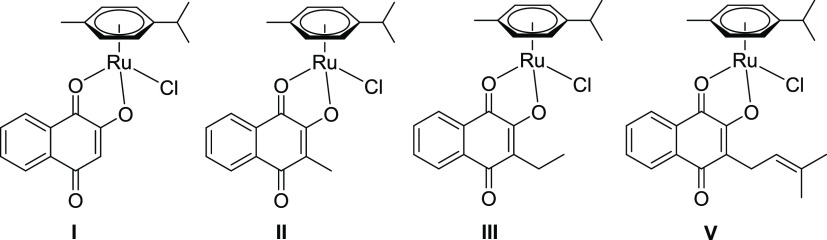
Parental naphthoquinone complexes with chloride as a leaving group (**I**–**III**, **V**).^30−33^

Thus, the Ru–*O*2 distances are the longest in all reported structures (approximately 212 pm), implying that cleavage and replacement by an auxiliary water or biomolecule might be feasible at this site (see discussion below). This may seem counterintuitive, as hemiaminal bonds are reportedly prone to easy dissociation in purely organic compounds.^53^ However, the organometallic’s newly formed five-membered ring (Ru-*N*1-*N*2-C1-*O*1) may stabilize this sensitive group. Furthermore, the C1–*N*1 distances are shorter, leading to increased stability. Comparison of the chlorido analogues of **2a** (**II**, R = methyl), **3a** (**III**, R = ethyl), and **5a** (**V**, R = 2-methyl-2-butene) revealed a shortened Ru–*O*1 bond and an elongated Ru–*O*2 bond upon coordination to a tridentate chelator. These findings oppose previous findings of organometallic complexes bearing *O*,*O*-chelates (Figure 4).^30,32,33^ Based on these observations, an activation via hydrolysis of the elongated Ru–*O*2 bond might be feasible, yielding an aqua complex, which has been reported for ruthenium arene complexes with halido leaving groups.^54−56^

### Aqueous Stability

#### UV–vis Photometry

**Figure 5:**
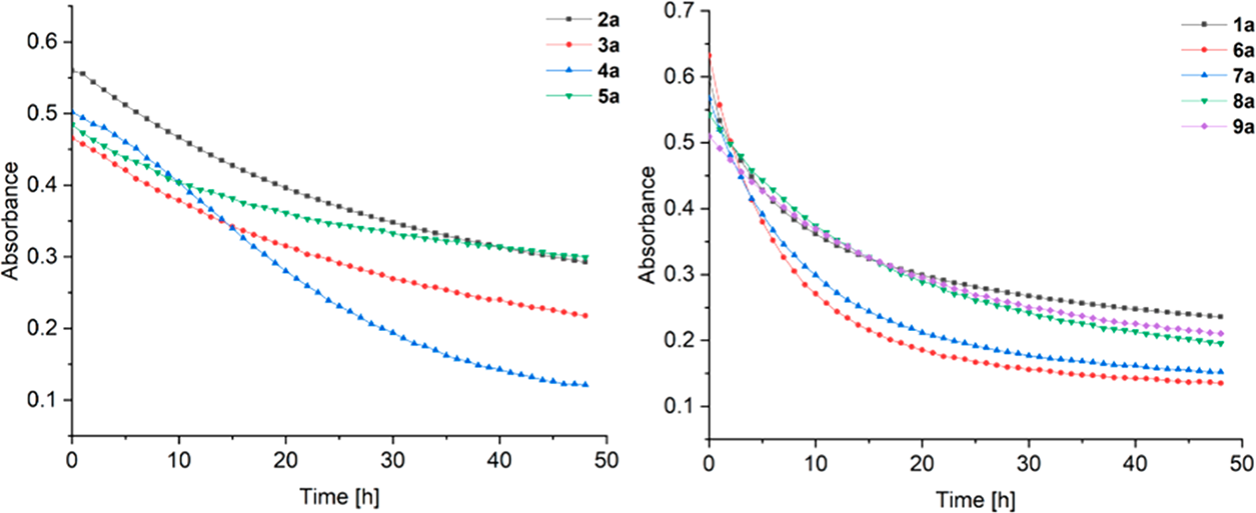
Absorption vs time of compound maxima (343–365 nm).

The aqueous stability of all complexes (**1a**–**9a**) was determined by UV–vis in phosphate buffered saline (PBS) solution at pH 7.4 at 20 °C over 48 h. Except for compound **4a** (only two maxima at 231 and 363 nm, respectively), all compounds (**1a**–**3a** and **5a**–**9a**) exhibited four maxima at around 231, 269, 356, and 465 nm, where the absorbance de- or increased over time (Figures S34–S42). The changes in absorbance could be best monitored at around 355 nm and indicated that the stability of the complex highly depends on the substituent at position 3 of the naphthoquinone moiety (Figure 5). Compounds **3a** and **5a** exhibit a nearly linear change in absorption, while complexes **1a** and **6a**–**9a** reacted faster in the first hours and afterward the line passes asymptotically against a certain absorbance. Based on the recorded data, substituents with a positive inductive effect like alkyl groups (**2a**–**5a**) stabilize the complex and lead to slower reactions in aqueous solution (Figure 5, left). In contrast, groups with a low or a negative inductive effect (**1a**, **6a**–**9a**) reduced the stability under physiological conditions (Figure 5, right). However, UV–vis measurements only give qualitative evidence of the occurrence of reactions but not which part of the complex is cleaved off or the formation of adducts. Therefore, HPLC-MS measurements of all complexes were performed to gather detailed information about the chemical behavior and reactivity in aqueous solution.

#### HPLC-MS

HPLC-MS experiments were performed with compounds **1a**–**4a** and **6a**–**9a** at physiological pH (7.4) at 20 °C with an eluent system of H_2_O/ACN. Unfortunately, poor water solubility prevented investigation of compound **5a**. UV–vis peaks and their corresponding mass ([M + H]^+^) for the neutral species can be observed at retention times between 14 and 20 min (Figures S43–S58).

**Figure 6:**
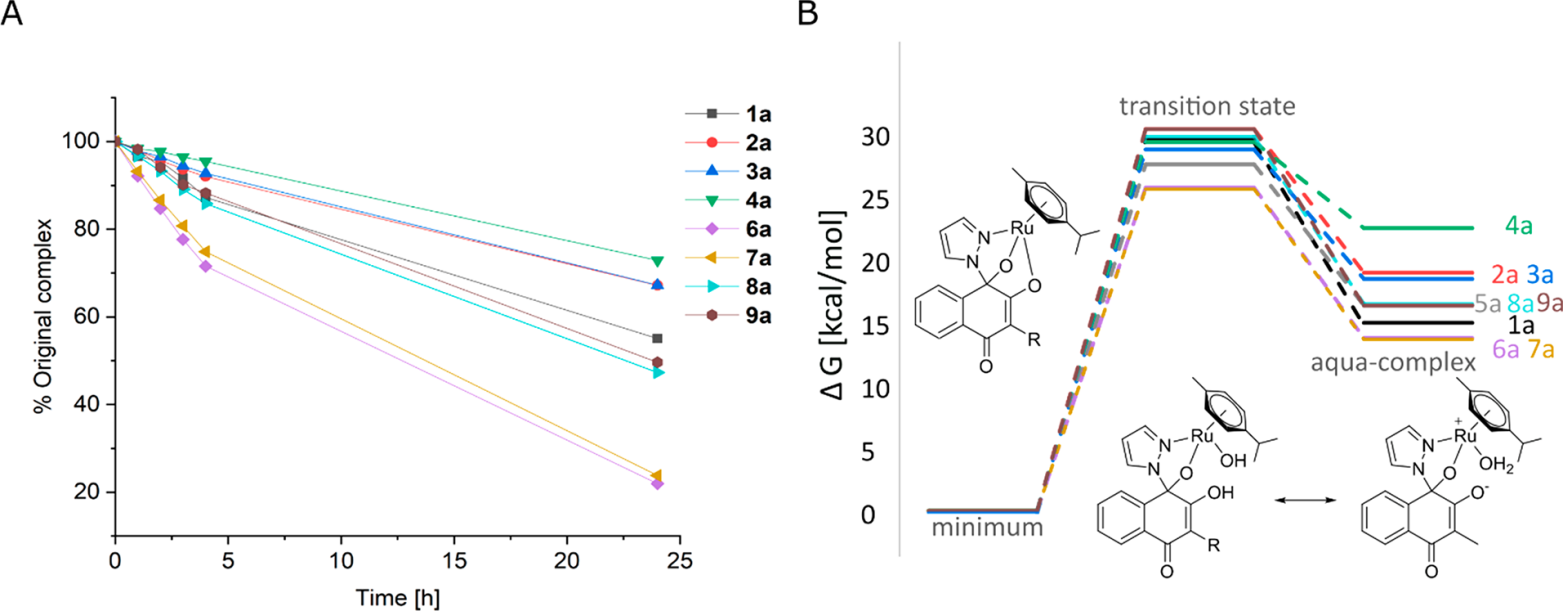
(A) Peak area of the original complex (obtained via HPLC measurements at physiological pH 7.4 at 20 °C with an eluent system of H_2_O/ACN) vs time of complexes **1a**–**4a** and **6a**–**9a**. (B) Gibbs free energy of each minimum energy structure, the aqua complex, and the corresponding transition state.

The experiments confirmed that compounds with a positive inductive effect (**2a**–**4a**) reacted relatively slow. On the other hand, complexes with halogens (**6a**, **7a**) and protonable groups (**8a**, **9a**) reacted rapidly and less than 50% of the original complex remained after 24 h (Figure 6). The highest stability was observed for **2a**, **3a**, and **4a**, where around 70% of the neutral complex was intact after 24 h.

**Scheme 3:**
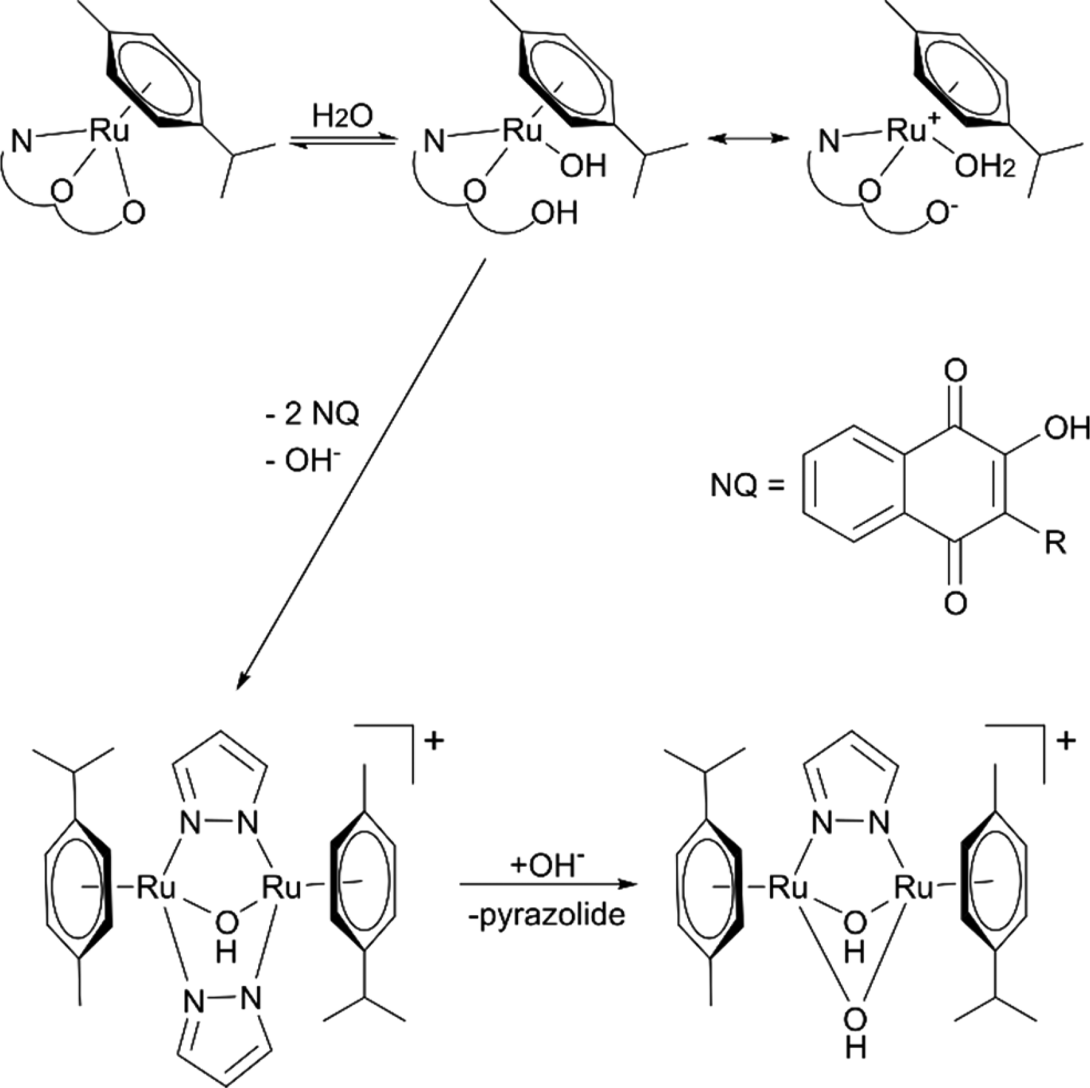
Postulated Hydrolysis Mechanism of Tridentate Naphthoquinone Complexes (**1a**–**9a**)

An interesting observation was the formation of two dimeric species in a time-dependent manner. The signals in the mass spectra at 11 and 15 min with *m*/*z* = 623.2 and 572.1 can be assigned to [((*p*-cymene)Ru)_2_(μ-OH)(μ-pyrazolate)_2_]^+^ and [((*p*-cymene)Ru)_2_(μ-OH)_2_(μ-pyrazolate)]^+^ (Figure S59). The formation of these dimeric compounds has been described in the literature before.^57^ Due to the low absorption of this compound, only mass signals were detectable. Based on HPLC-MS results, we postulate a hydrolysis mechanism of tridentate naphthoquinone complexes, where the most labile bond (Ru–*O*2) is cleaved and water coordinates to the metal center (Scheme 3). The newly formed hydroxido compound is in equilibrium with the corresponding aqua complex, which also has a neutral net charge. Two molecules of the aqua or hydroxido complexes can react with [((*p*-cymene)Ru)_2_(μ-OH)(μ-pyrazolate)_2_]^+^ by releasing the naphthoquinone ligands. In a subsequent step, this dimer can react with a hydroxyl ion and yield the bis-hydroxido compound [((*p*-cymene)Ru)_2_(μ-OH)_2_(μ-pyrazolate)]^+^. This hypothesis was the basis for further theoretical simulations to support the proposed mode of aquation.

### Theoretical Studies

To complement experimental observations, theoretical calculations at the density functional theory (DFT) level of theory were conducted. The crystal structures of **1a**–**7a** and **9a** were taken as starting points for subsequent structure relaxations using the PBEh-3c method.^46^ The respective structures related to the corresponding minimum energy are in good agreement with experimental values. The computed bond distances are given in Table S16 for comparison to the experimental values in Table 1.

In order to investigate the probability of the hydrolysis of the complexes and to support the previous assumption of Ru–*O*2 cleavage upon hydrolysis, the aqua complex was optimized along with the transition state of the aquation process. The starting guess of the latter was obtained from a nudged elastic band calculation.^49^ The energies of the aqua complexes (negative binding energies) that report the stability of the aqua complexes relative to the free complexes and the free water molecules are given along with the relative Gibbs free energies of the corresponding transition states in Table 2.^58^ All energy values are reported relative to the initial complex and a separate water molecule, computed with the same level of theory. As it is visible, the aqua complex is energetically unfavored compared to the initial complex and a free water molecule. Remarkably, the stabilities of the aqua complexes differ strongly from each other. While complex **4a** shows the most unfavorable energetics regarding the formation of an aqua complex, complexes **6a** and **7a** show very favorable energetics. Further, complexes **2a** and **3a** show similar energetics and aquation is also energetically unfavored compared to complexes **1a**–**7a**. Aqua complexes **1a**, **5a** as well as **8a**–**9a** are energetically in between the most stable and most unstable structures. It can be seen that aqua complex **1a** is slightly lower in energy; however, the barrier to overcome, i.e., the energy of its transition state, is among the highest.

**Table 2:** Gibbs Free Energy of the Transition State and Aqua Complex **1a**–**9a** Computed with the PBEh-3c Method

	transition state Δ*G* (kcal/mol)	aqua complex Δ*G* (kcal/mol)
**1a**	30.15	15.27
**2a**	31.06	19.31
**3a**	29.54	18.99
**4a**	29.95	22.98
**5a**	28.21	16.62
**6a**	26.30	14.00
**7a**	26.16	13.90
**8a**	30.42	16.78
**9a**	31.03	16.70

In addition to the energetics, the geometrical features were analyzed. A correlation between the bond distances of Ru to *O*2 and *O*2 and the closest C atom could be found (Figures S69A and B). Both bond distances are smaller, the higher the binding energy is and the less likely the aqua complex is formed. These results are consistent with chemical intuition, as they suggest stronger bonds indicated by shorter bond lengths. Furthermore, these results indicate that the breaking of the Ru–*O*2 bond is one key factor in the hydrolysis of these complexes. In addition, the binding energy of each complex is plotted against the bond distances in Figure S69.

As can be seen in the reaction profile in Figure 6, the stability measurements correlate with the theoretical studies, where **2a**, **3a**, and **4a** exhibit the highest stability, while **6a** and **7a** hydrolyze very fast. The reaction barriers are similar for all complexes, and the stability depends predominantly on the relative energy of the respective aqua complex supporting the postulated aquation mode.

### Amino Acid Interaction Studies

The targets and mode of action of ruthenium arene compounds heavily depend on the ligand sphere. Both interactions with peptides and proteins (e.g., RAPTA-C) and DNA (e.g., RM175) have been reported in the literature.^15,59^ A key factor for the anticancer activity of metal-based compounds lies in adduct formation with sulfur- and nitrogen-containing amino acids (e.g., l-methionine, l-cysteine, and l-histidine).^60−62^ In order to investigate the behavior of the complexes of this work toward possible biological targets, *N*- and *C*-protected amino acids (*N*-acetyl-l-His-OMe, *N*-acetyl-l-Cys-OMe, and *N*-acetyl-l-Met-OMe) were incubated with the most stable (**4a**), most active (**3a**), as well as least stable (**6a**) and least active compound (**8a**) (for activity data, see the section “MTT Assay” below).

**Figure 7:**
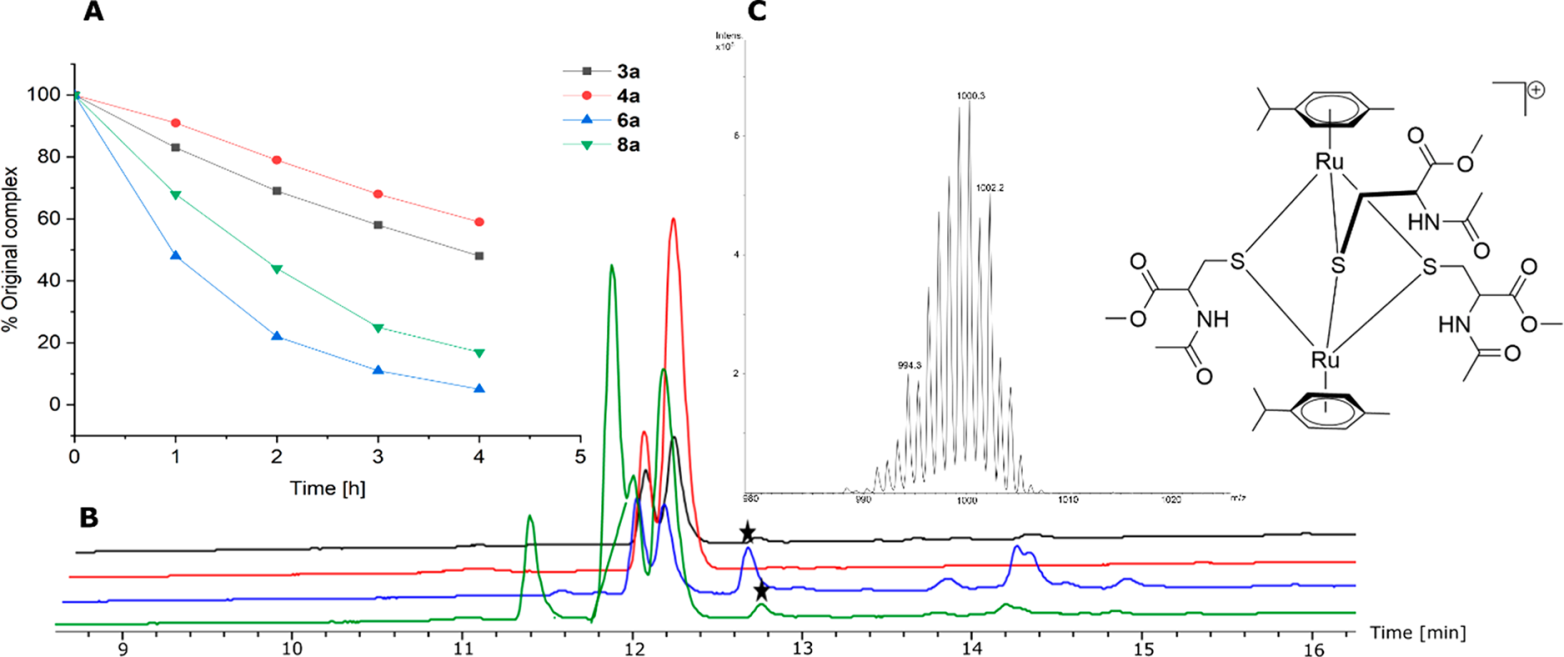
(A) Peak area of initial complexes vs time in the presence of *N*- and C- protected amino acids. (B) Chromatograms of**3a** (black), **4a** (red), **6a** (blue), and **8a** (green) after 1 h in the presence of *N*-acetyl-l-His-OMe, *N*-acetyl-l-Cys-OMe, and *N*-acetyl-l-Met-OMe at 37 °C; the stars indicate [((*p*-cym)Ru)_2_(μ-*S*-*N*-acetyl-l-Cys-OMe)_3_]. (C) Mass spectra and structure of the trithiolato bridged dimer.

Amino acids accelerate the decomposition of the initial organometallic, as indicated by the reduced area of the complex signals in the chromatograms (**3a**, **4a**, **6a**) (Figure 7, Figures S60–S67). The amount of **8a** was calculated by increase of the ligand’s peak area, since the complex signal overlaps with other signals, which would distort the peak area.

After 1 h of incubation time, only 50% (**6a**) and 68% (**8a**) of the initial complexes can be detected and formation of a trithiolato bridged dimer [((*p*-cym)Ru))_2_(μ-*S*-*N*-acetyl-l-Cys-OMe)_3_] (12.6 min, *m*/*z* = 1000.3) can be observed (Figure 6). In contrast, more than 80% of **3a** and **4a** was intact and only minor amounts of dimer could be measured after 1 h. After 4 h, only traces of intact **6a** and **8a** could be detected. These results show that more active (**3a**) and stable (**4a**) complexes exhibit higher inertness against thiols. The formation of trithiolato bridged ruthenium dimers indicated a high affinity to thiol groups, which are an important group when studying binding sites of biomolecules (e.g., HSA) and glutathione (GSH). Especially GSH plays an important role in cancer biology (e.g., cell protection and proliferation, DNA synthesis and resistances).^63^ Contrary to the amino acid free stability studies, [((*p*-cymene)Ru)_2_(μ-OH)(μ-pyrazolate)_2_]^+^ and [((*p*-cymene)Ru)_2_(μ-OH)_2_(μ-pyrazolate)]^+^ could not be detected under the applied conditions. The longer incubation time yielded complex spectra with various adducts and decomposition products. Thus, further experiments are necessary for detailed information about the additional formed species and also regarding the substitution mechanism of the tridentate ligand by thiols.

### Biological Studies

#### MTT Assay

Cytotoxicity of compounds was determined against three different human cancer cell lines (A549 (non-small cell lung cancer), SW480 (colon cancer), and CH1/PA-1 (ovarian teratocarcinoma)) by MTT assays (Table 3). Compared to the free ligands, complexation led to an increase of cytotoxicity in almost all cell lines. The highest increase was observed for complexes **2a** and **3a** with 3 orders of magnitude in SW480 colon cancer cells, yielding IC_50_ values in the nanomolar range (46 nM for **3a**), which is in the same range as the most active ruthenium arene compounds reported until now (20 nM).^64−66^ With exception of the Mannich products **8a** and **9a**, the complexes (**1a**–**7a**) exhibited high activity in A549 cells (0.76–65 μM), contrary to many metal arene compounds, which are typically more active in chemo-sensitive CH-1/PA-1 cells.^67−70^

**Table 3:** Cytotoxicity of Hydroxy-1,4-naphthoquinones (**1**–**9**), Their Corresponding Complexes (**1a**–**9a**), and the Parental Chlorido Compound (**I**–**III**, **V**)^a^

compound	A549 (μM)	SW480 (μM)	CH1/PA-1 (μM)
**1**(30)	157 ± 13	247 ± 17	246 ± 24
**1a**(29)	65 ± 3	13 ± 3	160 ± 7
**2**(31)	210 ± 32	116 ± 37	129 ± 29
**2a**(29)	1.2 ± 0.2	0.094 ± 0.031	>50
**3**(33)	158 ± 22	101 ± 11	173 ± 10
**3a**	0.76 ± 0.14	0.046 ± 0.007	62 ± 5
**4**	10 ± 1	12 ± 2	13 ± 2
**4a**	2.1 ± 0.3	0.28 ± 0.03	9.1 ± 0.6
**5**(32)	42 ± 14	5.5 ± 0.6	3.3 ± 0.2
**5a**	2.9 ± 0.3	0.31 ± 0.01	4.0 ± 0.7
**6**	237 ± 24	168 ± 36	128 ± 29
**6a**	54 ± 17	1.5 ± 0.3	122 ± 4
**7**	265 ± 24	152 ± 8	141 ± 32
**7a**	42 ± 9	1.3 ± 0.2	115 ± 5
**8**	>200	160 ± 26	>200
**8a**	>200	33 ± 2	146 ± 18
**9**	>100	130 ± 24	164 ± 24
**9a**	>200	45 ± 5	119 ± 6
**1*****H*****-pyrazole**(29)	>200	>200	>200
**I**(30)	98 ± 24	86 ± 20	84 ± 15
**II**(31)	47 ± 4	15 ± 3	31 ± 10
**III**(33)	75 ± 9	27 ± 1	71 ± 12
**V**(32)	20 ± 5	4.1 ± 1.5	4.1 ± 0.6

a 50% inhibitory concentrations in human carcinoma cell lines A549, SW480, and CH1/PA-1. Values are means ± SDs obtained by the MTT assay (exposure time: 96 h).

The synthesized complexes **1a**–**3a** and **6a**–**9a** showed extraordinarily high activity in intrinsically chemo-resistant cancer cell lines (SW480, A549), where **4a** and **5a** are highly cytotoxic in all three cell lines. These results indicate that the substituent in position 3 of the naphthoquinone backbone highly affects the cytotoxicity in SW480 cells, where alkyl chains and halides enhanced activities. Furthermore, the synthesized complexes (**1a**–**3a**, **5a**–**7a**) exhibited improved cytotoxicity compared to the parental chlorido complexes (**I**–**III**, **V**) (Figure 4, Table 3). Complexes bearing a labile halido leaving group tend to hydrolyze quickly, yielding the respective aqua complex. The formed species reacts with biological donor molecules. Additionally, the observed fast cleavage of the naphthoquinone ligand in aqueous solution also hampers the transport of intact complex to tumor cells. However, the tridentate ligand scaffold prevents fast hydrolysis, due to the lack of a labile leaving group. Further experiments are necessary to elucidate the remarkable cytotoxic behavior in SW480 cells.

#### ROS Assay

Reactive oxygen species (ROS) play an important role in cellular functions, such as signal transduction, by modifying the structure of proteins, transcription factors, and genes or signaling cell growth, regulation of enzyme activity, and elimination of pathogens.^71^ The main endogenous ROS source is mitochondria, where they are produced as byproducts of oxidative phosphorylation. However, NADPH oxidase, peroxisomes, cytochrome P450, endoplasmic reticulum, and lysosomes also produce reactive oxygen species.^71,72^

**Figure 8:**
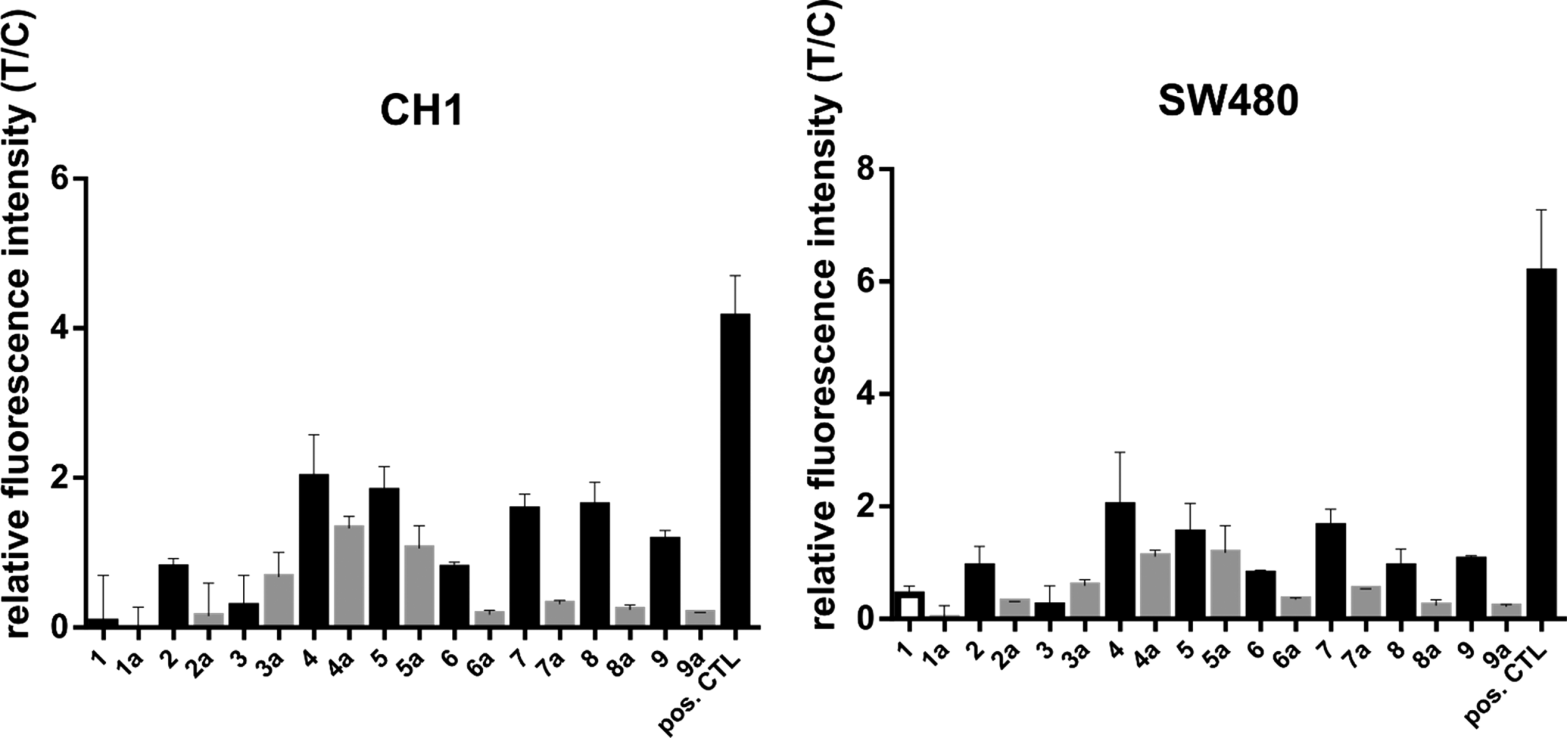
ROS levels upon treatment with ligands **1**–**9** and complexes **1a**–**9a** in CH1/PA-1 and SW480 cancer cells, reflected by relative fluorescence intensity (T/C). Values are means ± SDs obtained with the DCFH-DA assay (exposure time: 2 h, *c* = 200 μM). Positive control: TBHP.

Cancer cells have an accelerated metabolism, due to their hyperproliferation and higher ROS levels. Additionally, cancerous cells exhibit a higher concentration of antioxidants. The balance between ROS and antioxidants is responsible for cell survival. Hence, this equilibrium is a promising target for anticancer therapy, as provoking/causing additional oxidative stress may ultimately result in cell death. Thus, employing compounds which increase ROS levels in cancer cells or decrease the antioxidant concentration may be a possible approach.^73^ Since naphthoquinones are known as redox-active and ROS-producing compounds, ligands **1**–**9** and their corresponding complexes (**1a**–**9a**) were investigated for ROS formation in cancer cells (Figures S73–S78).^28^ The biggest difference in cytotoxicity was observed between SW480 and CH1/PA-1 cells; thus, the ROS assay was performed in these two cell lines, in order to determine if ROS generation might be responsible for the differences in cytotoxicity. Increased ROS levels were observed for **4**–**9** at concentrations of 20 and 200 μM, while **1**–**3** act as antioxidants at higher concentrations (Figure 8). Contrary to the free naphthoquinone ligands, the complexes show an antioxidant effect, especially at higher concentrations (200 μM). ROS formation was merely observed for **4a** and **5a** at 200 μM. The results showed similar ROS formation in both cancer cell lines, indicating that reactive oxygen species cannot account for the differences in cytotoxic activity. Overall, it seems that the formation of ROS does not contribute to the remarkable cytotoxic properties of the complex.

#### Plasmid Assay

**Figure 9:**
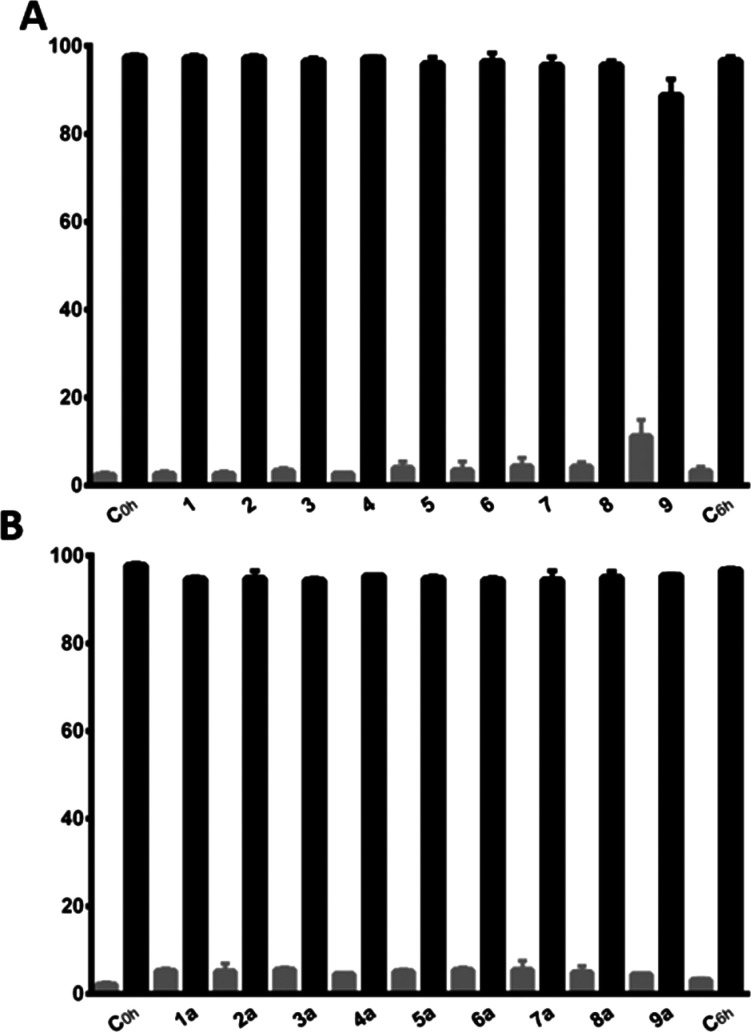
Quantified data from the electrophoretic plasmid DNA interaction studies shown in Figure S81. The black columns correspond to the open-circular (oc) form and white columns to the supercoiled (sc) form of the pUC19 plasmid after 6 h of incubation and the controls. (A) Results for ligands**1**–**9**. (B) Results for complexes **1a**–**9a**.

The cell-free dsDNA plasmid assay serves to figure out whether compounds are capable of altering the secondary structure of DNA (which may result from various forms of interaction such as cross-linking, intercalation, or strand breakage) and, hence, whether DNA could be a possible target. The plasmid dsDNA is mostly present in negatively supercoiled (sc) form, and upon interaction with the compounds, it may gradually converge to the open circular (oc) form in terms of electrophoretic mobility or may be converted into oc, linear, or interhelically cross-linked DNA. Based on the quantified data, the ruthenium-containing complexes induce, on average, about 2 times more formation of the oc form of the plasmid DNA than their ligand counterparts. Interestingly, compound **9** shows the highest ability (11 ± 4%) to induce nicks, resulting in complete untwisting of the supercoiled form to the open circular form (Figure 9). However, this effect is very minor and neither qualitatively nor quantitatively comparable with platinum drugs which strongly interfere with DNA by cross-linkage and have DNA as their main target.^74^ This behavior was also observed for *p*-cymene complexes bearing mono- and bidentate pyridine derivatives.^19,75^ In summary, compounds **1**–**9** and **1a**–**9a** show rather negligible induction of DNA strand breaks in the plasmid and no signs of cross-linking within 6 h of incubation (Figure S79) in comparison to the positive control (Figure S80).

## Conclusion

A series of nine tridentate naphthoquinone-based ruthenium arene complexes was synthesized and characterized by 2D NMR spectroscopy, X-ray diffraction, and elemental analyses. The behavior in aqueous solution was studied by UV/vis and HPLC-MS experiments. Based on the obtained data, hydrolysis via cleavage of the Ru–*O*2 bond was postulated and this assumption could be confirmed by DFT calculations. Drug stability is a crucial factor in preclinical development and massively impacts the potency of novel metallodrugs. Overall, substituents at position 3 of the naphthoquinone backbone with a positive inductive effect (**2a**–**5a**) improved the aqueous stability and decelerated the formation of dimeric ruthenium arene species ([((*p*-cym)Ru)_2_(μ-OH)(μ-pyrazolate)_2_]^+^ and [((*p*-cym)Ru)_2_(μ-OH)_2_(μ-pyrazolate)]^+^). Furthermore, amino acid incubation studies have shown a high affinity toward thiol-containing residues, which is important information for future investigations with biomolecules (e.g., HSA and GSH). Substituents with alkyl chains (and +*I* effect) lead to increased cytotoxicity in colon and lung cancer cell lines (A549 and SW480), where the highest activity (46 nM) was observed in SW480 cells for complex **3a**. The cytotoxicity in chemosensitive CH1/PA-1 cancer cells is tremendously lower for almost all of these complexes. In ROS assays and plasmid interaction assays, the complexes neither caused notable increases in ROS levels nor plasmid interactions. Thus, their cytotoxic potency does not arise from ROS formation nor DNA interactions. Future work will be devoted to elucidate the mode of action of these highly potent organometallics.

## Supporting Information Available

Materials, NMR spectra of ligands (Figures S1–S8) and complexes (Figures S9–S26), experimental parameters for X-ray analysis, crystal structures (Figures S27–S33), sample and crystal data, data collection, and structure refinement (Tables S1–S15), UV–vis spectra (Figures S34–S42), HPLC and HPLC-MS chromatograms (Figures S43–S67), optimized structures (Figure S68), bond lengths (Tables S16–S18), bond distance vs binding energy (Figure S69), concentration–effect curves (Figures S70–S72), ROS assay results (Figures S73–S78), and electropherograms (Figures S79–S80) (PDF)

## Accession Codes

CCDC 2067988–2067994 contain the supplementary crystallographic data for this paper. These data can be obtained free of charge via www.ccdc.cam.ac.uk/data_request/cif, or by emailing data_request@ccdc.cam.ac.uk, or by contacting The Cambridge Crystallographic Data Centre, 12 Union Road, Cambridge CB2 1EZ, UK; fax: +44 1223 336033.
