# Supplementary material for: Tridentate 3-Substituted Naphthoquinone Ruthenium Arene Complexes: Synthesis, Characterization, Aqueous Behavior, and Theoretical and Biological Studies
Source: Inorg Chem. 2021 Jun 11;60(13):9805–19. doi: 10.1021/acs.inorgchem.1c01083 (PMC8261824; doi:10.1021/acs.inorgchem.1c01083)
Supplement: Supplementary file 1 — ic1c01083_si_001.pdf [file ic1c01083_si_001.pdf]

Supporting Information

Tridentate 3-substituted naphthoquinone ruthenium arene complexes: Synthesis, characterization, aqueous behavior, theoretical and biological studies

*Heiko Geisler,[a] Julia Westermayr,[b], Klaudia Cseh,[a] Dominik Wenisch,[a] Valentin Fuchs,[a] Sophia Harringer,[a] Sarah Plutzar,[a] Natalie Gajic,[a] Michaela Hejl,[a] Michael A. Jakupec,[a,c] Philipp Marquetand,[d,e] and Wolfgang Kandioller[a,c]**

[a] *University of Vienna, Faculty of Chemistry, Institute of Inorganic Chemistry, Waehringer Str. 42, 1090 Vienna, Austria; E-Mail: wolfgang.kandioller@univie.ac.at; Tel.: +43 1 4277 52609*

[b] *University of Warwick, Department of Chemistry, Gibbet Hill, Coventry, CV47AL, United Kingdom*

[c] *Research Cluster "Translational Cancer Therapy Research", University of Vienna, Waehringer Str. 42, 1090, Austria*

[d] *University of Vienna, Faculty of Chemistry, Institute of Theoretical Chemistry, Waehringer Str. 17, 1090 Vienna, Austria*

[e] *Vienna Research Platform on Accelerating Photoreaction Discovery, University of Vienna, Währinger Str. 17, 1090 Wien, Austria*

Contents

Experimental section 3

Materials 3

X-ray Analysis 17

Stability measurements 25

UV-Vis spectra 25

HPLC-MS 30

Amino acid incubations studies 35

Theoretical studies 37
Bond lengths 38
Biological studies.. 40
MTT assay 40
ROS assay (DCFH-DA assay) 43
Plasmid assay 49
References.. 50

Experimental section

Materials

Following chemicals were purchased from commercial suppliers: 2-Methyl-1,4-naphthoquinone, Na_2CO_3, Lawsone, $Fe(NO_3)_3$ nonahydrate, 3,3-dimethylallyl bromide, $Pd(Ph_3)_4$, NEt_3, 2,3-dichloro-1,4-naphthoquinone, 2,3-dibromo-1,4-naphthoquinone and 1*H*-pyrazole were from Acros-Fisher. $NaHCO_3$, acetaldehyde, S-proline, $NaBH_4$, thiomorpholine, *N*-acetyl-L-cysteine methyl ester from Sigma-Aldrich. HCl, SiO2, NaOH, KOH, and 30% H_2O_2 from VWR. Hantzsch ester (TCI), *N*-acetyl-L-methionine methyl ester (Iris Biotech), *N*-acetyl-L-histidine methyl ester (BLDpharm), cyclohexene (FLUKA) and $RuCl_3$ xH_2O (Johnson Matthey).

NMR Spectra

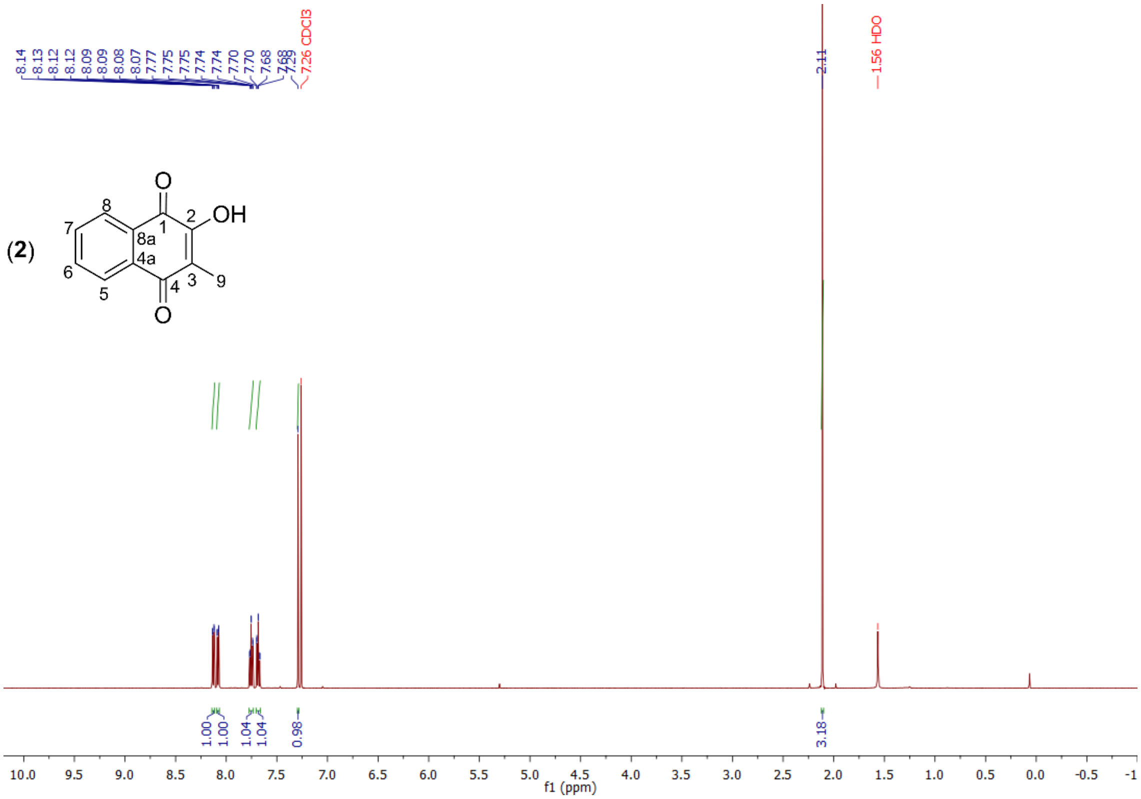

Figure S 1: ^{1}H-NMR ($CDCl_3$, 500.10 MHz) spectrum of (**2**)

(3)

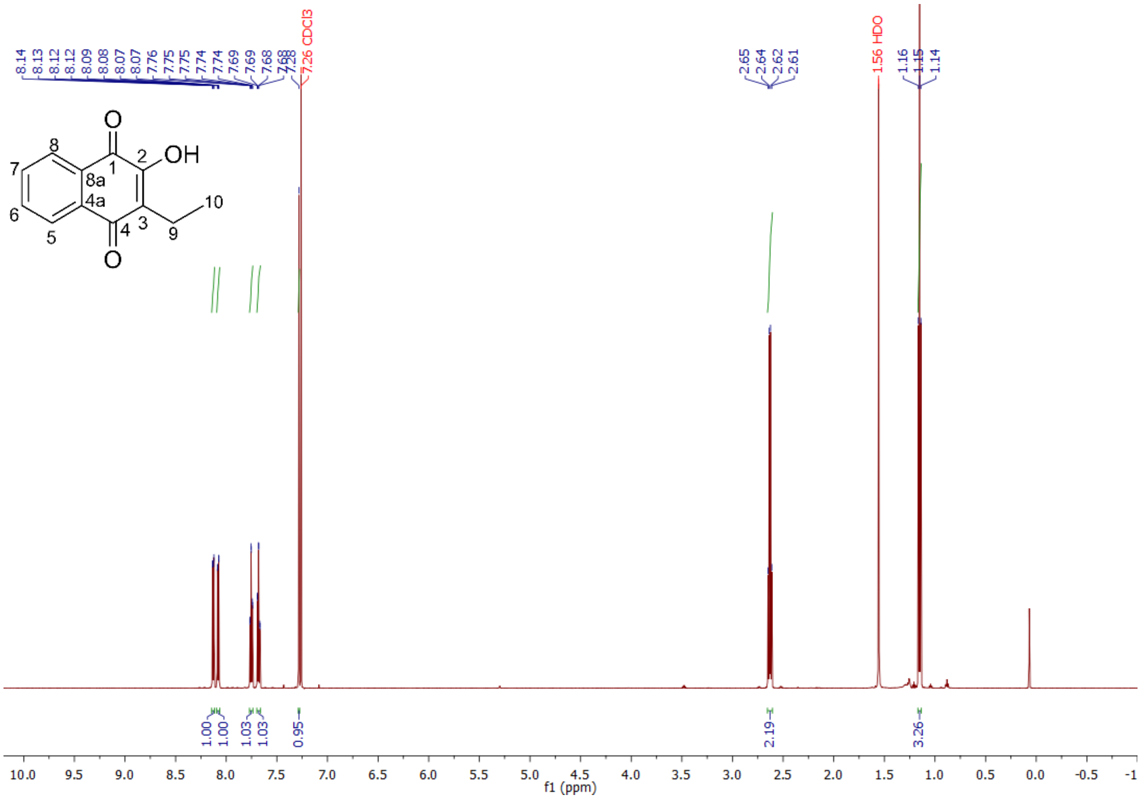

Figure S 2: ^{1}H-NMR ($CDCl_3$, 600.25 MHz) spectrum of (**3**)

(4)

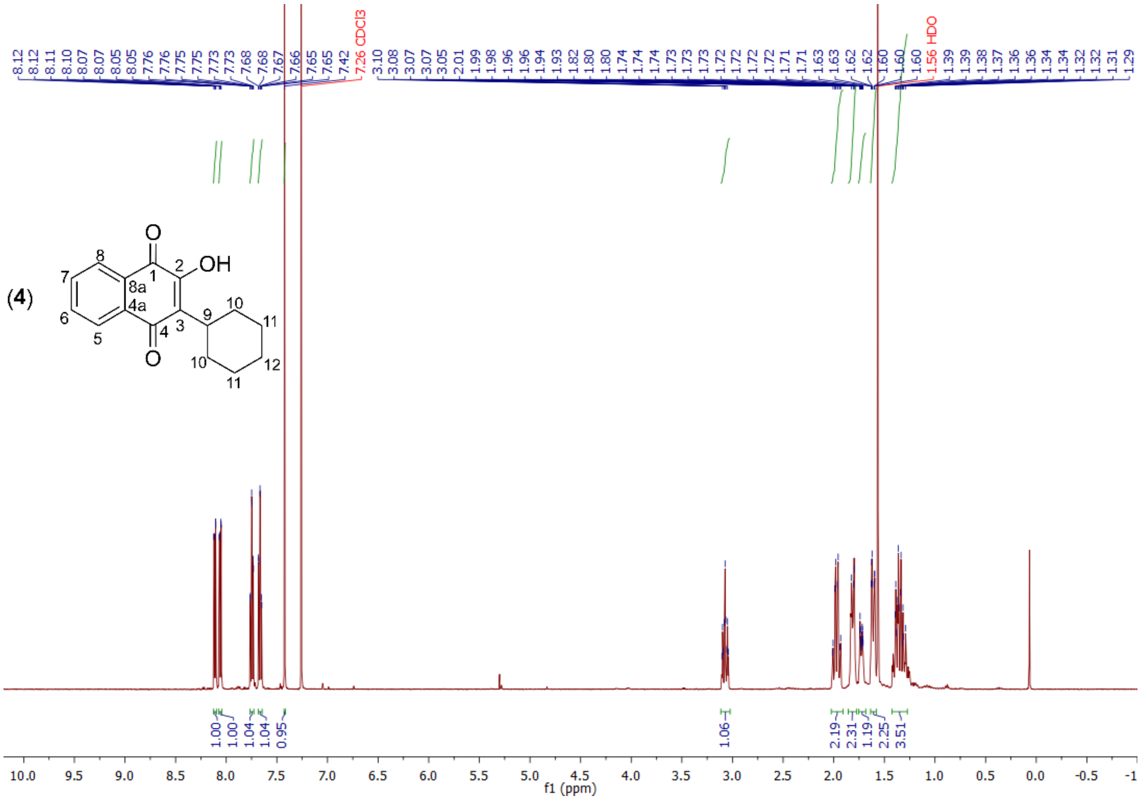

Figure S 3: ^{1}H-NMR ($CDCl_3$, 500.10 MHz) spectrum of (**4**)

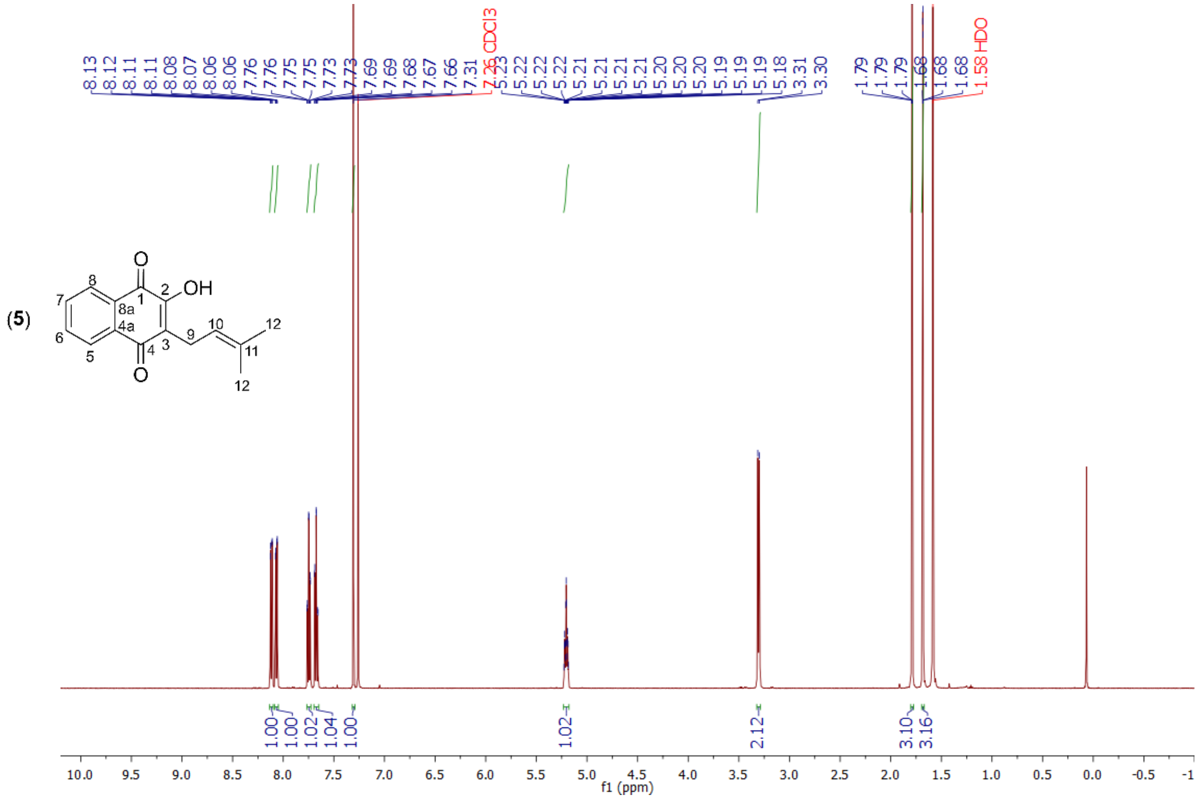

Figure S 4: ^{1}H-NMR ($CDCl_3$, 500.10 MHz) spectrum of (**5**)

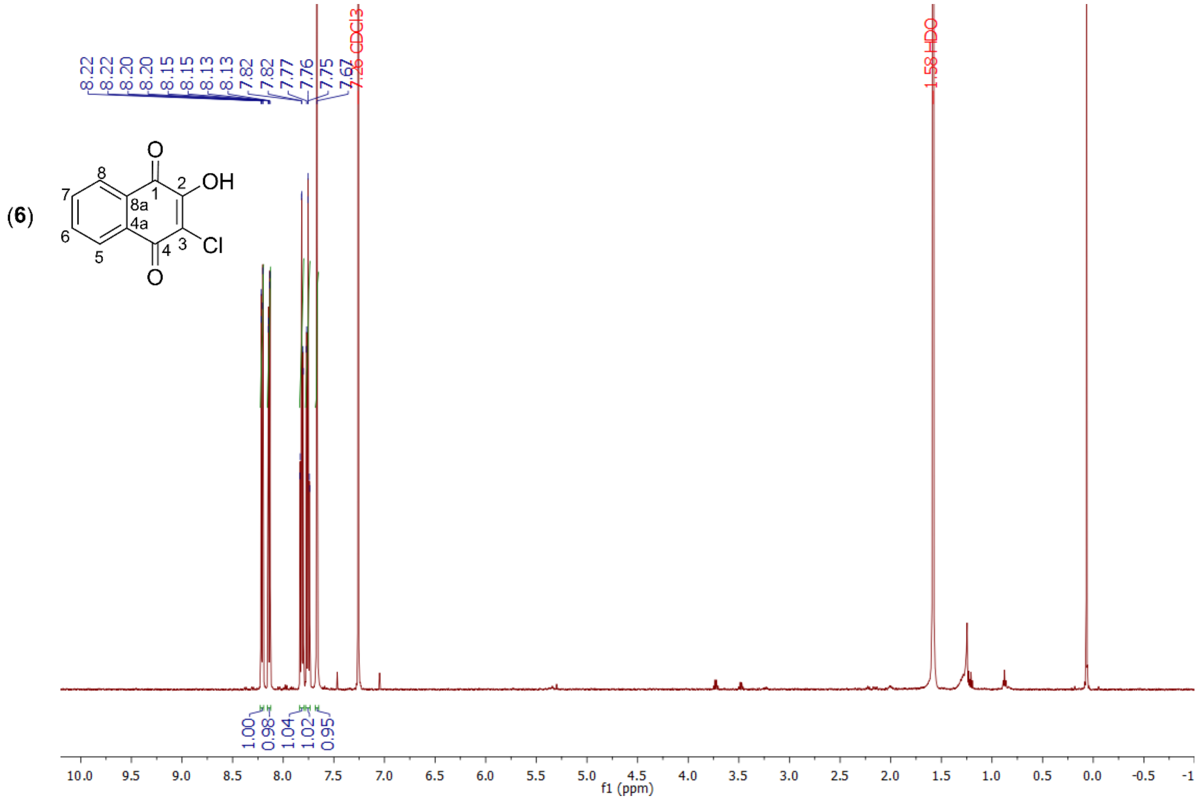

Figure S 5: ^{1}H-NMR ($CDCl_3$, 500.10 MHz) spectrum of (**6**)

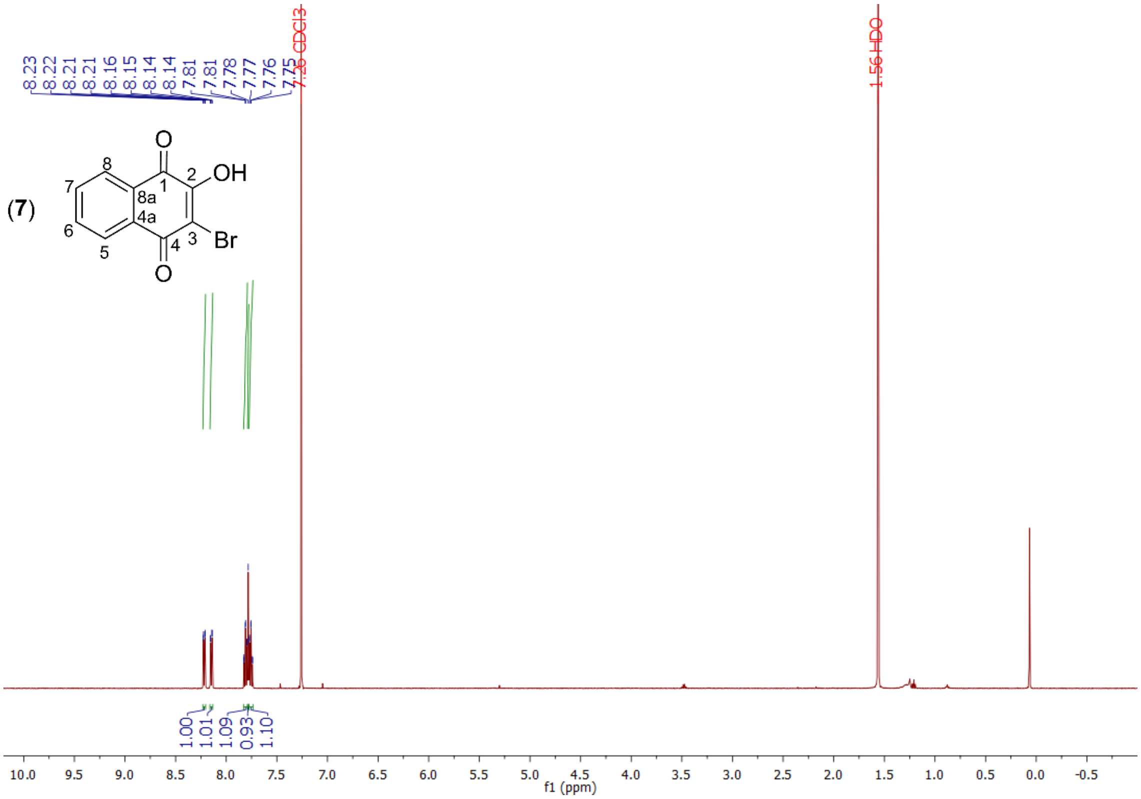

Figure S 6: ^{1}H-NMR ($CDCl_3$, 500.10 MHz) spectrum of (**7**)

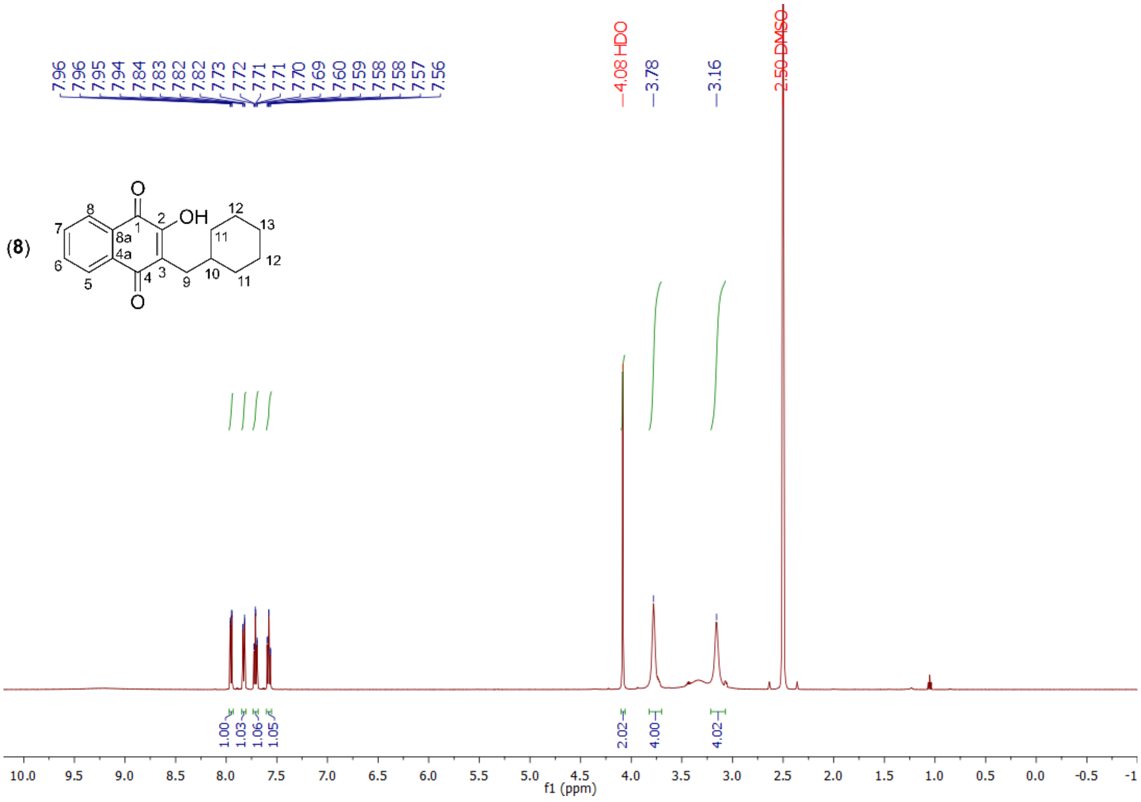

Figure S 7: ^{1}H-NMR (DMSO-d_6, 500.10 MHz) spectrum of (**8**)

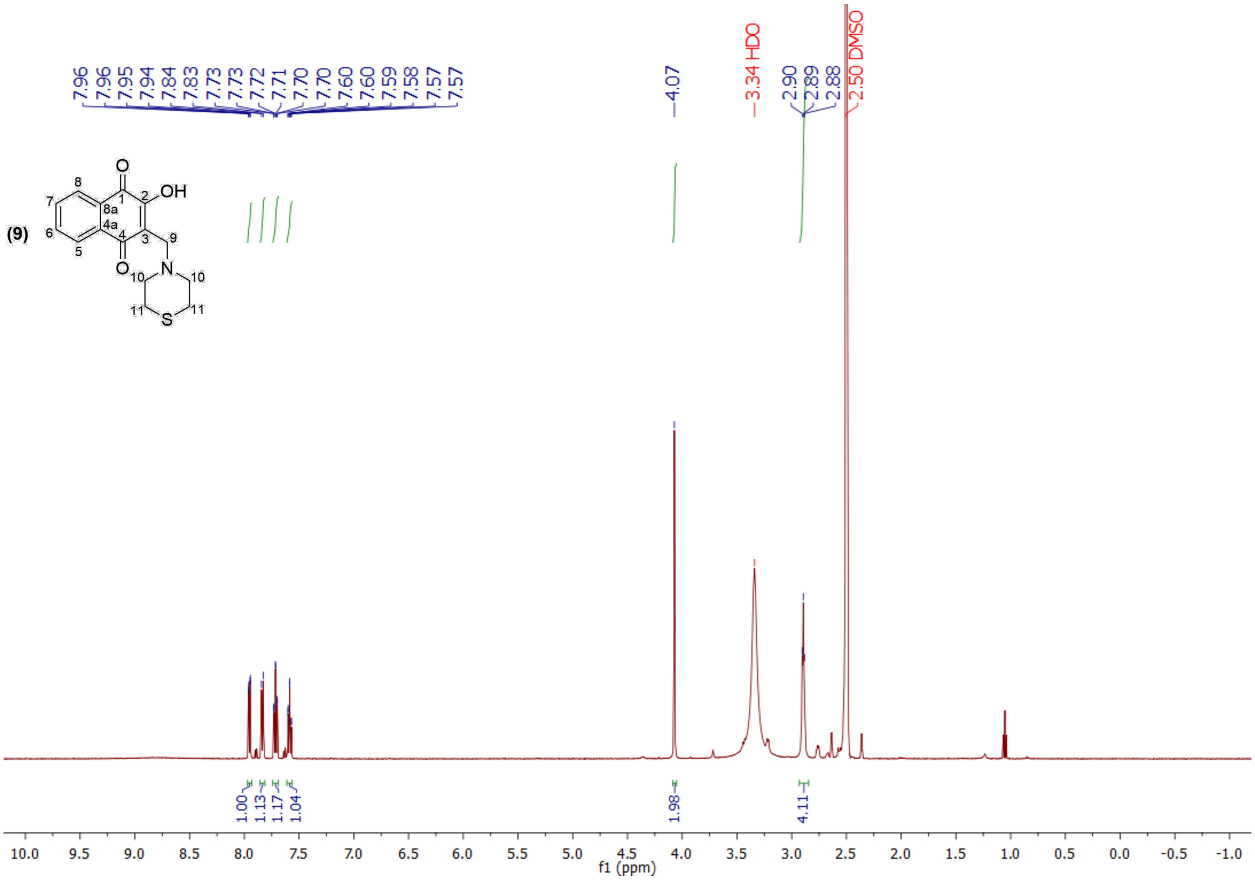

Figure S 8: ^{1}H-NMR (DMSO-d_6, 500.10 MHz) spectrum of (**9**)

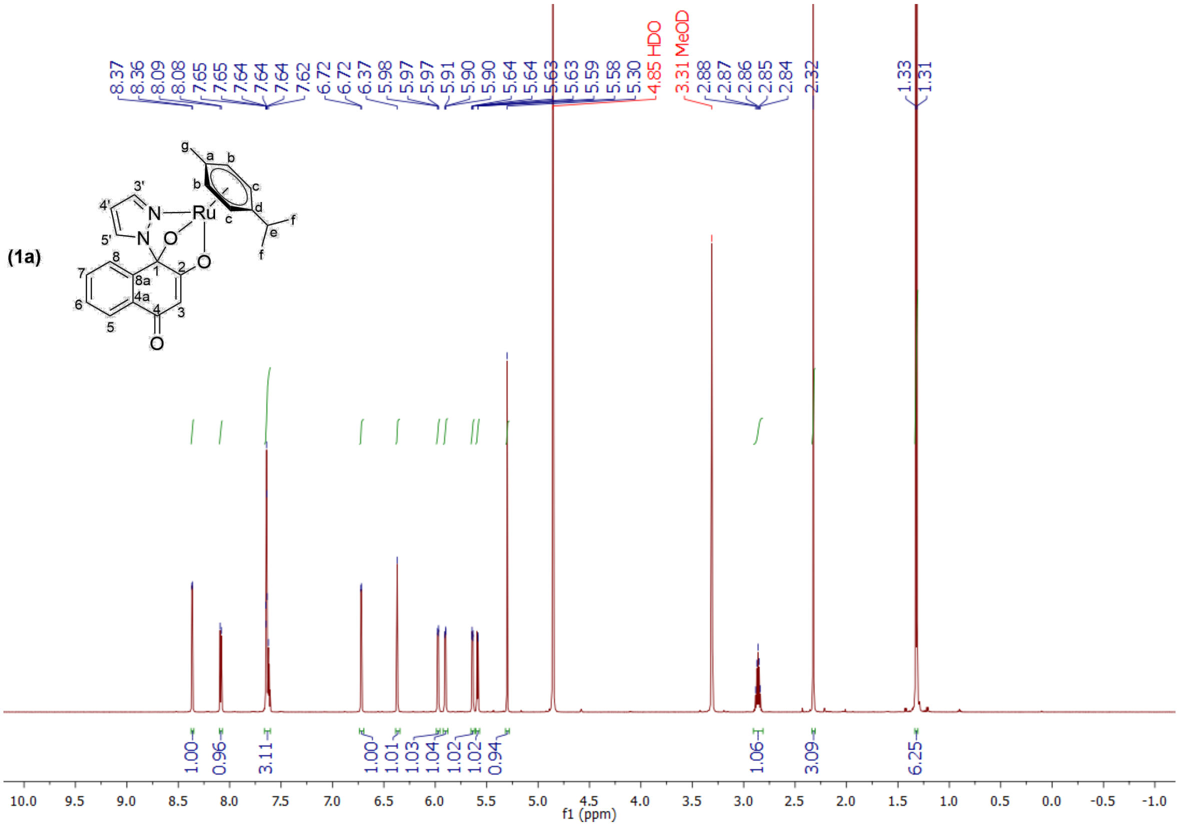

Figure S 9: ^{1}H-NMR (MeOH-d_4, 600.25 MHz) spectrum of (**1a**)

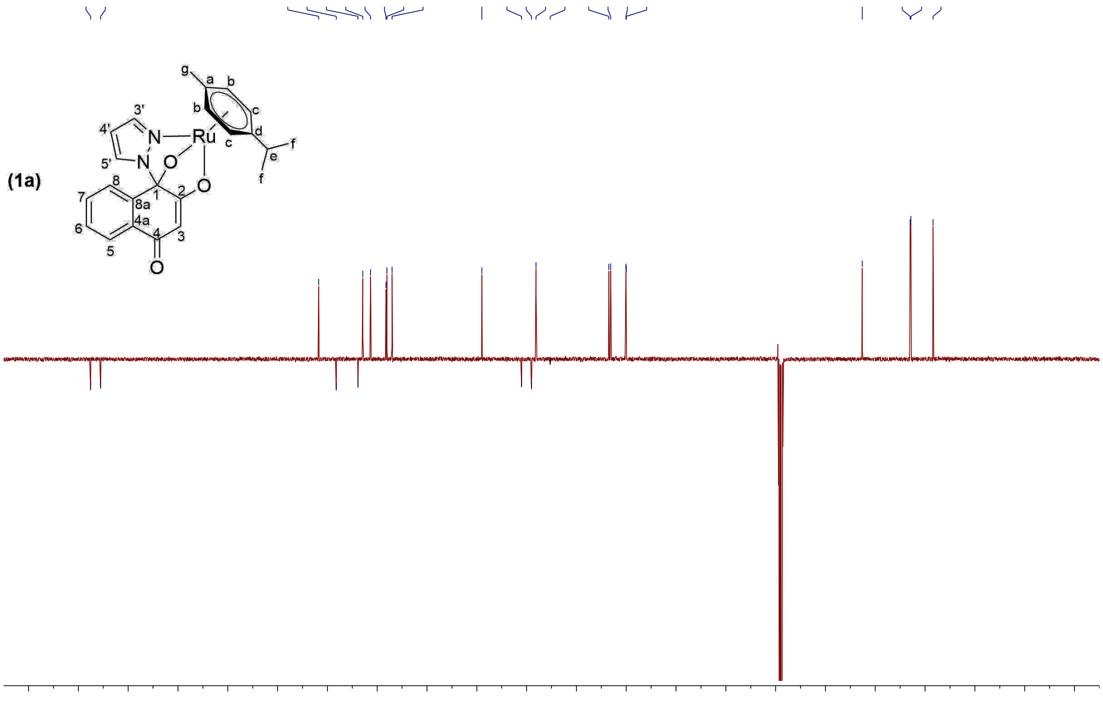

Figure S 10: ^{13}C-NMR (MeOH-d_4, 600.25 MHz) spectrum of (**1a**)

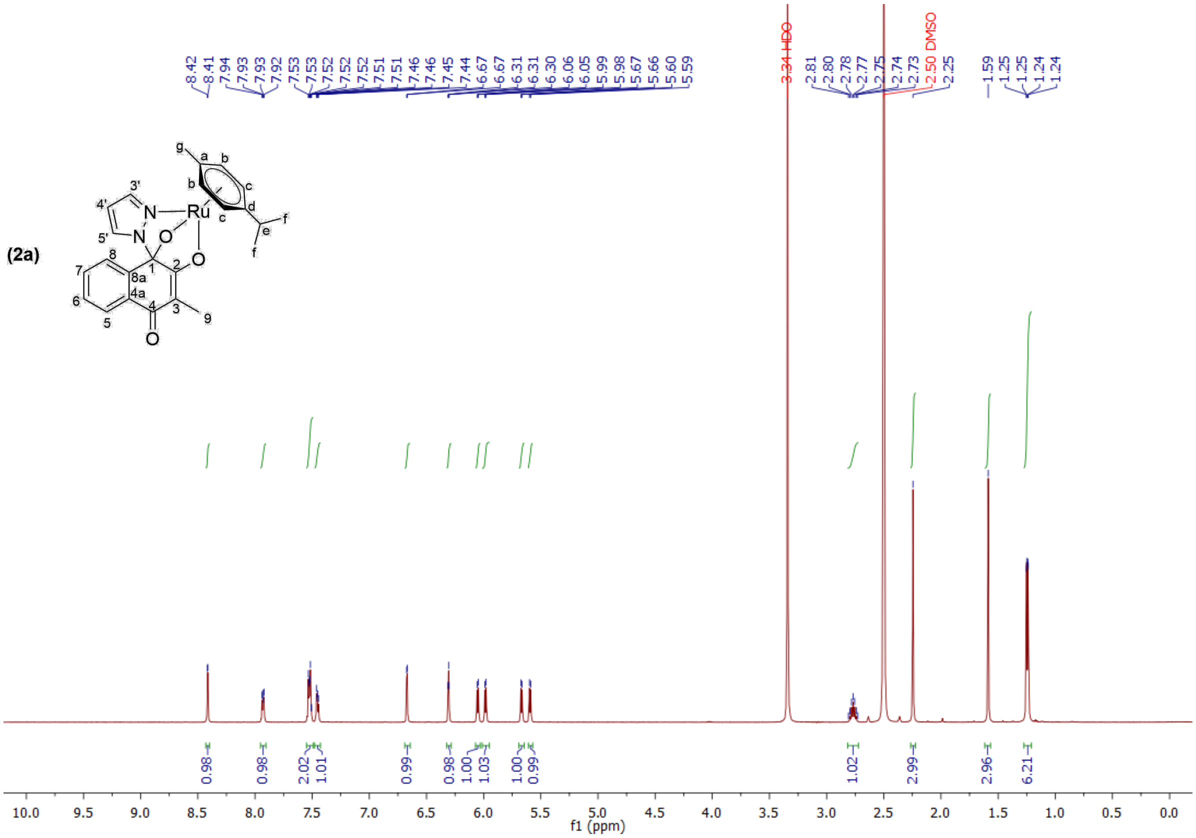

Figure S 11: ^{1}H-NMR (DMSO-d_6, 500.10 MHz) spectrum of (**2a**)

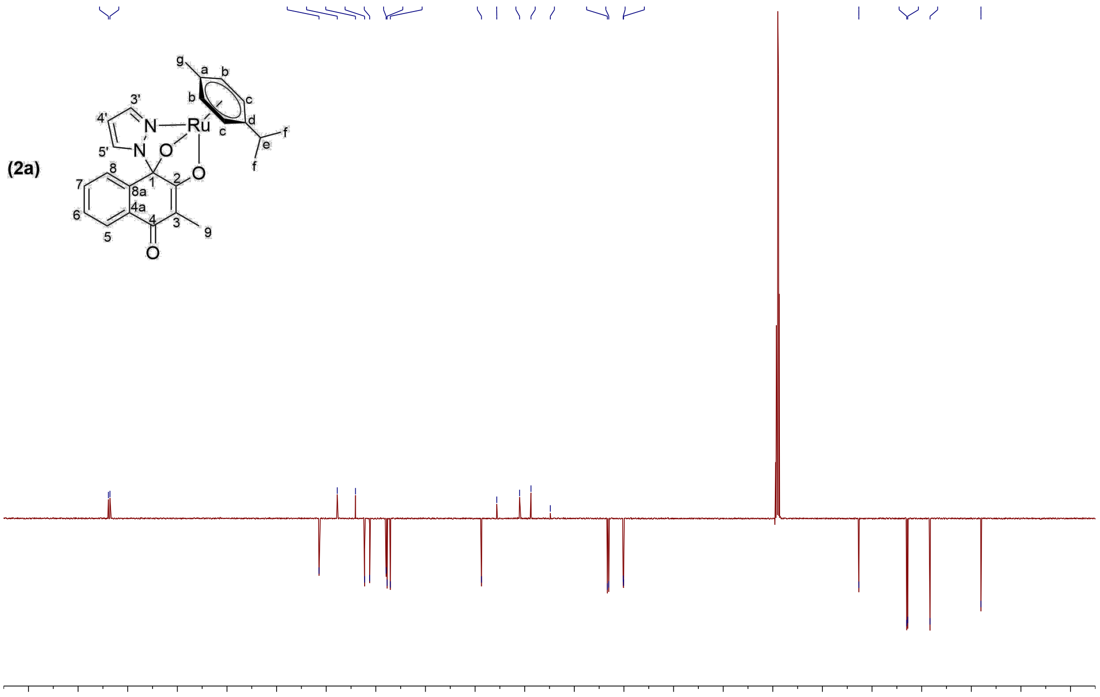

Figure S 12: ^{13}C-NMR (MeOD-d_4, 600.25 MHz) spectrum of (**2a**)

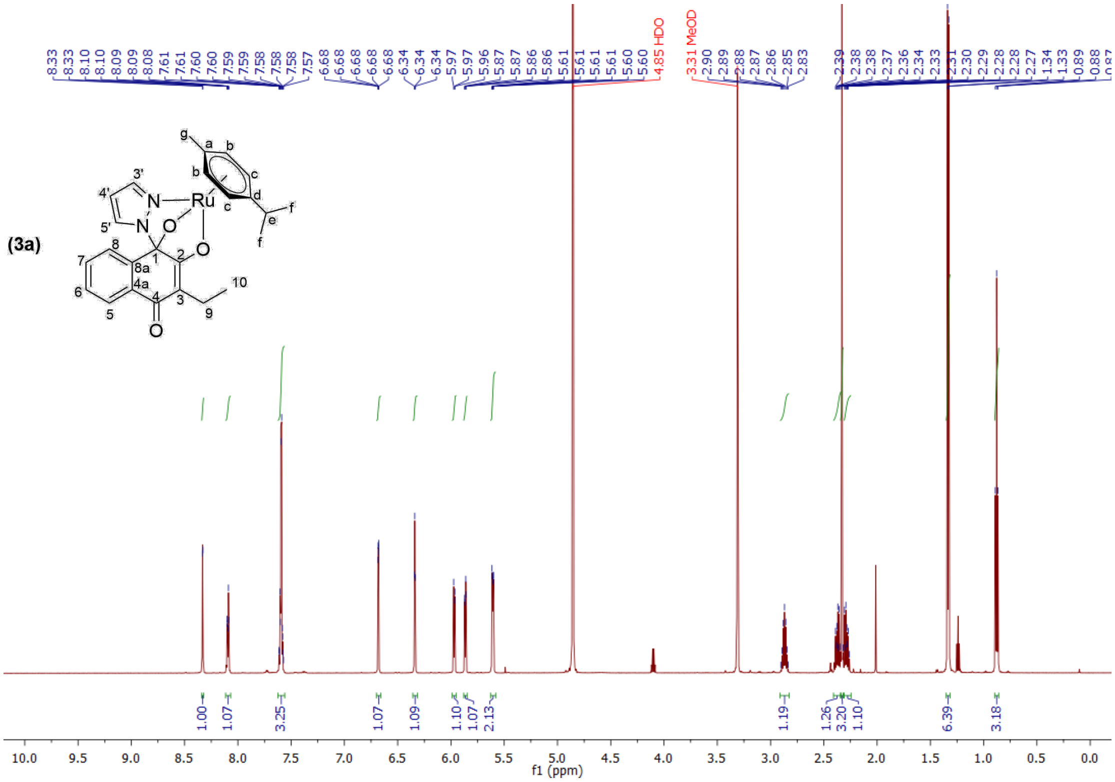

Figure S 13: ^{1}H-NMR (MeOD-d_4, 600.25 MHz) spectrum of (**3a**)

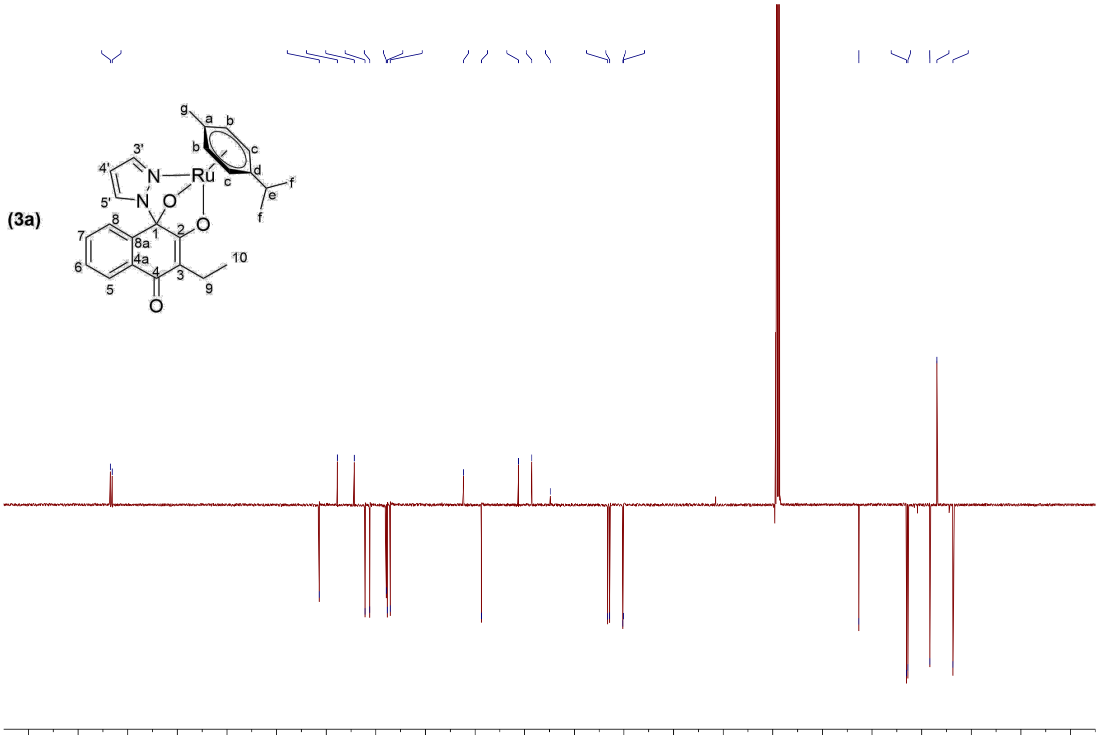

Figure S 14: ^{13}C-NMR (MeOD-d_4, 600.25 MHz) spectrum of (**3a**)

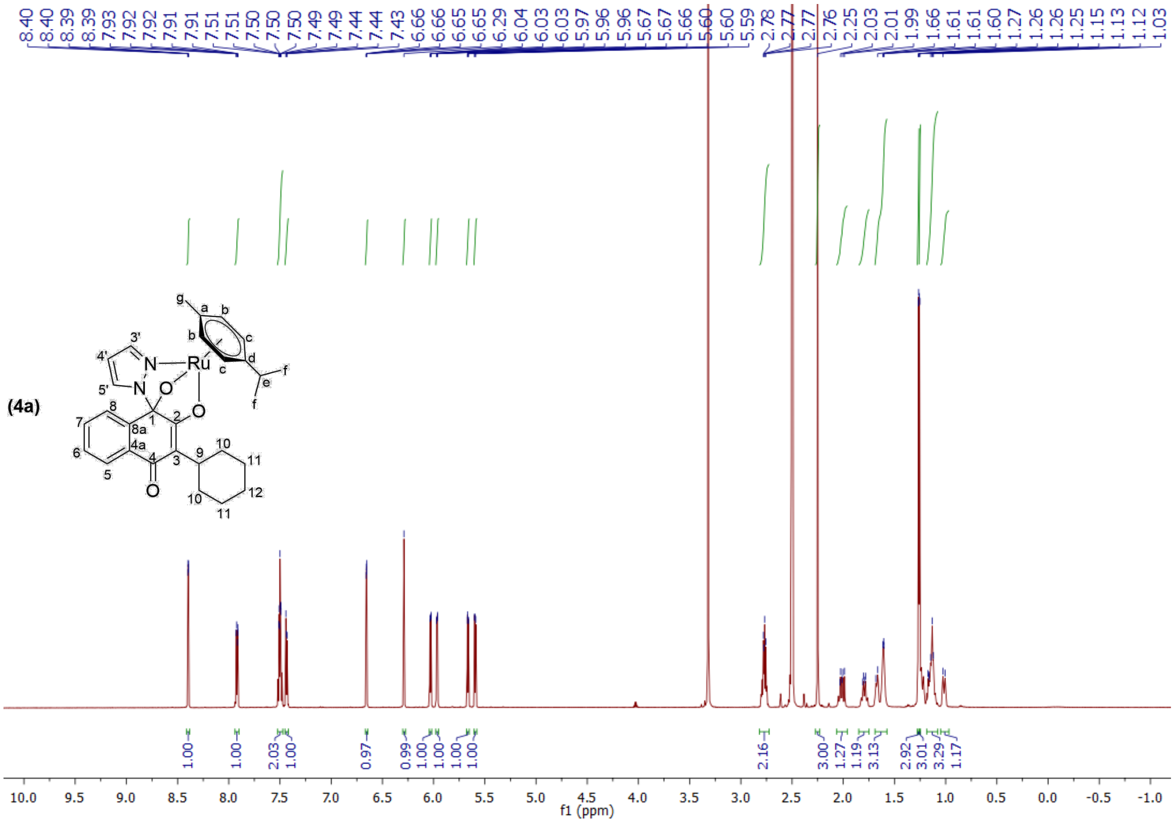

Figure S 15: ^{1}H-NMR (MeOD-d_4, 600.25 MHz) spectrum of (**4a**)

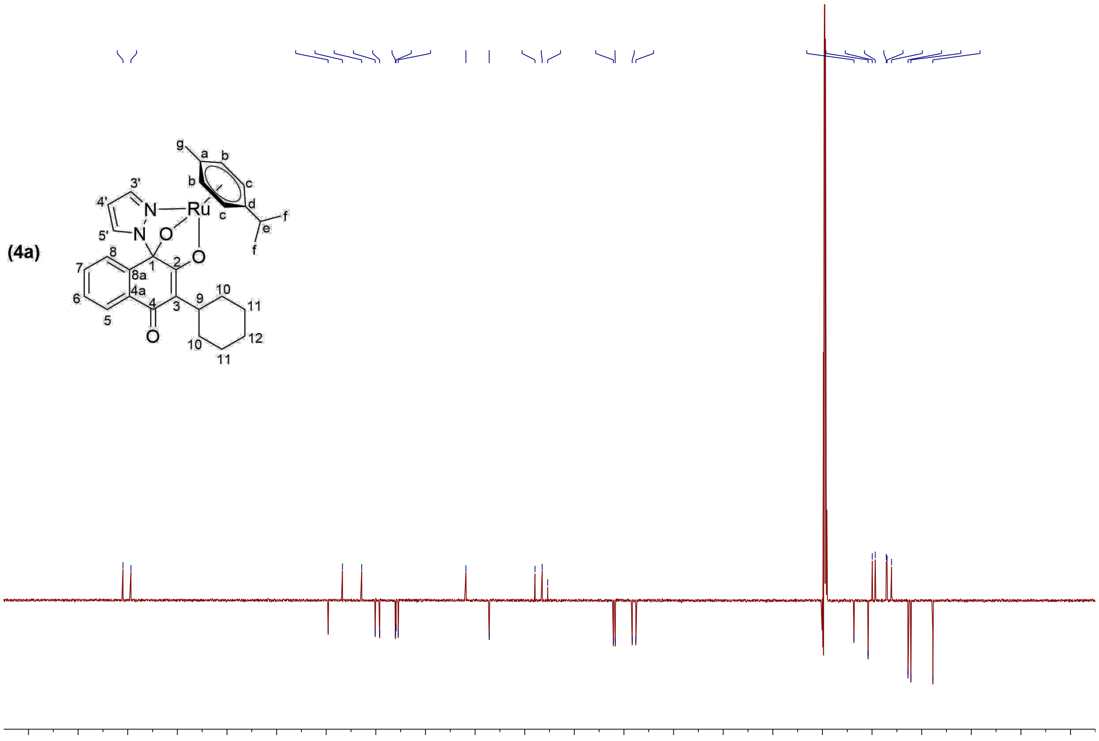

Figure S 16: ^{13}C-NMR (DMSO-d_6, 600.25 MHz) spectrum of (**4a**)

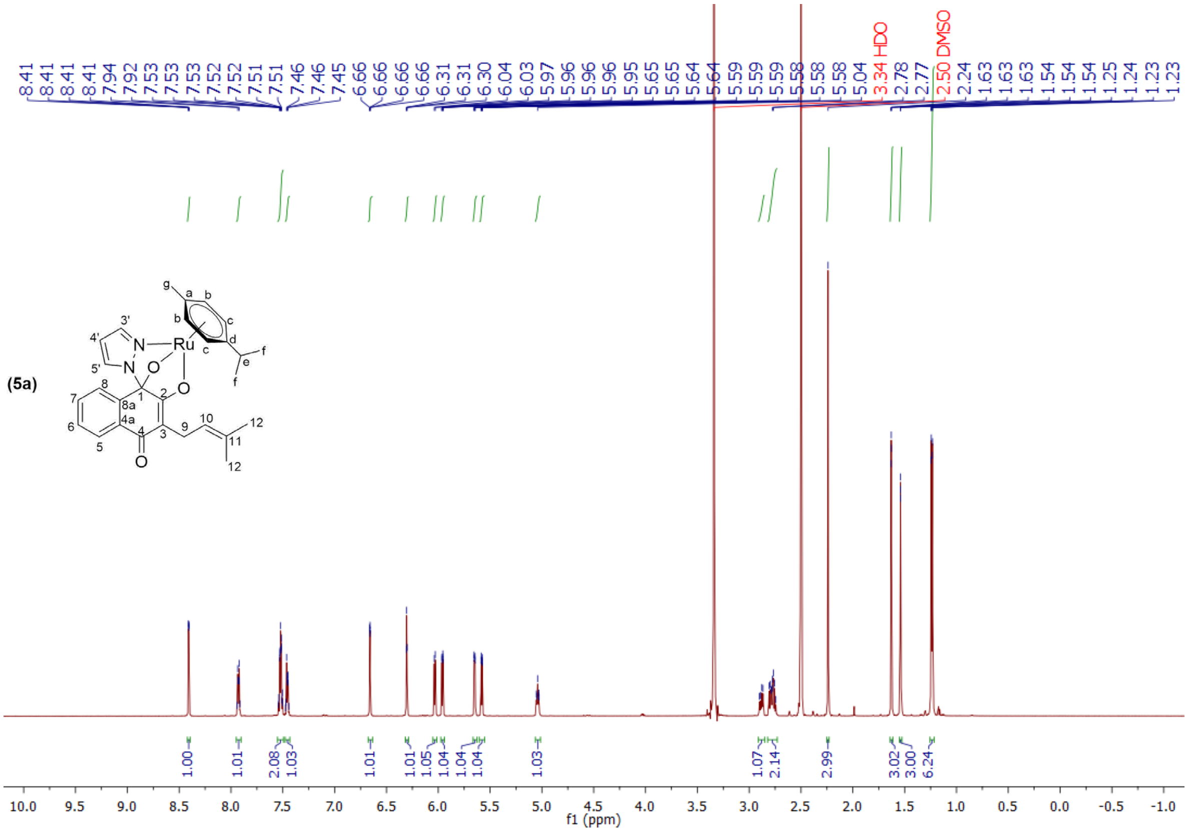

Figure S 17: ^{1}H-NMR (MeOD-d_4, 600.25 MHz) spectrum of (**5a**)

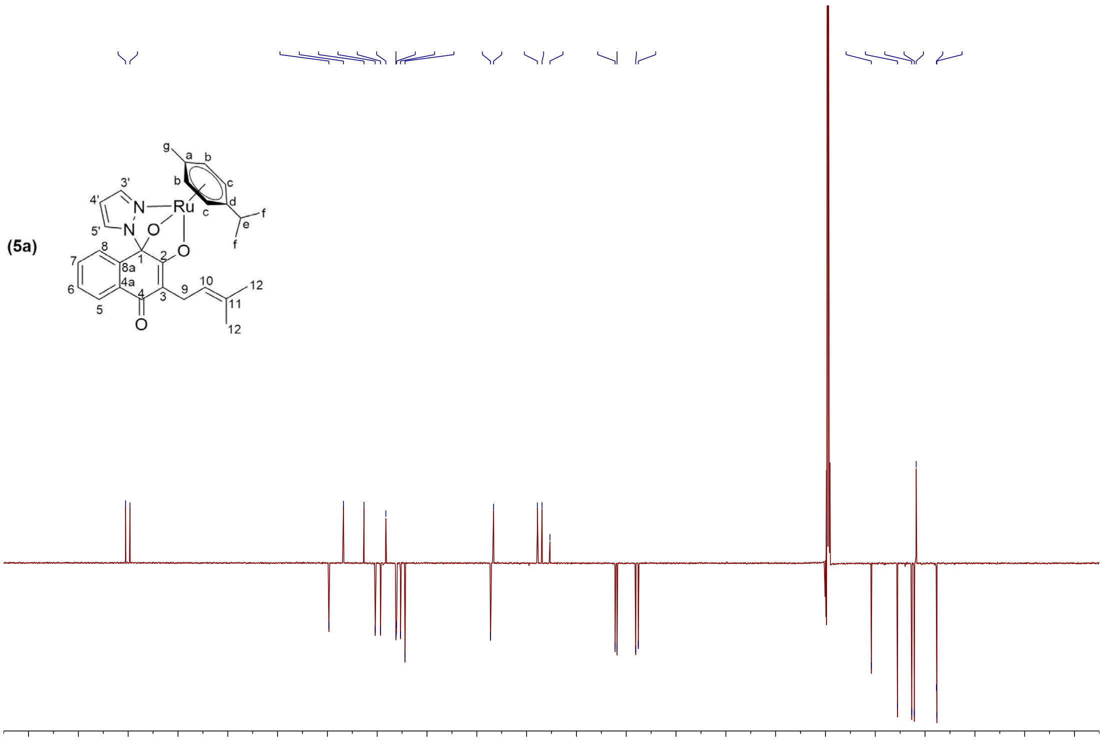

Figure S 18: ^{13}C-NMR (DMSO-d_6, 600.25 MHz) spectrum of (**5a**)

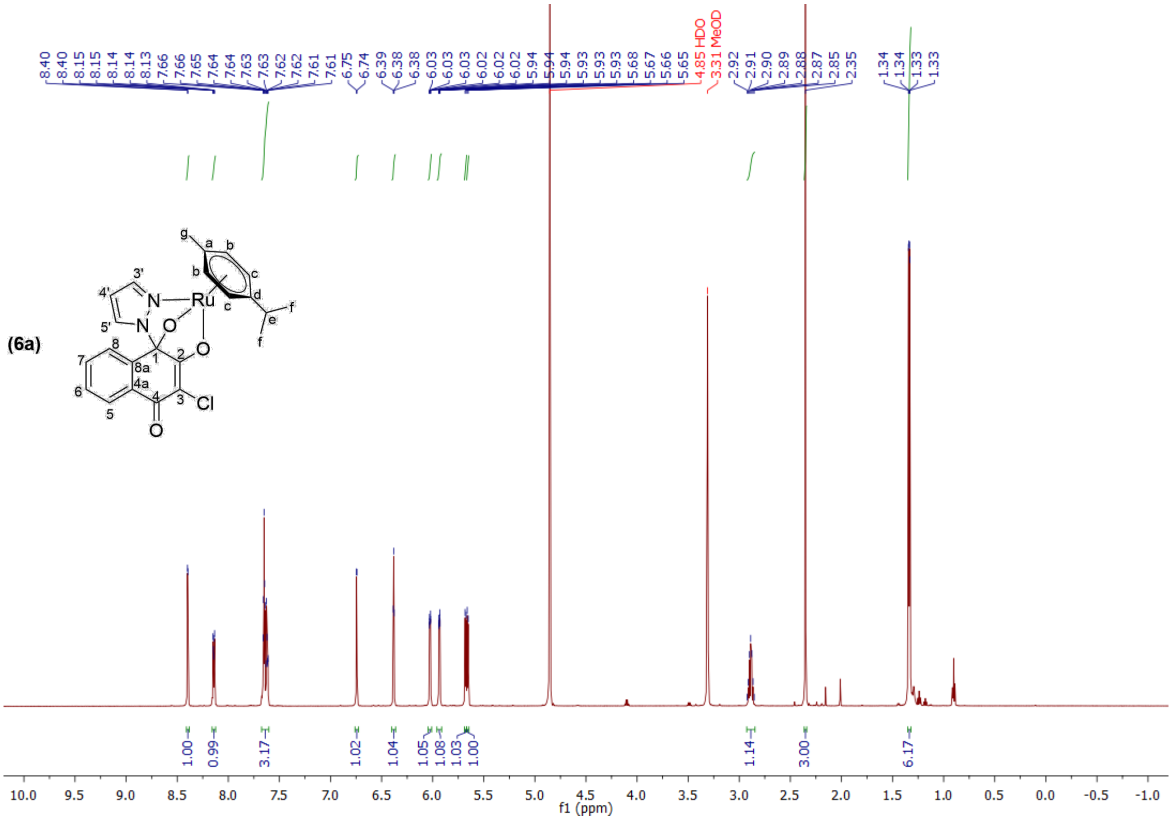

Figure S 19: ^{1}H-NMR (MeOD-d_4, 600.25 MHz) spectrum of (**6a**)

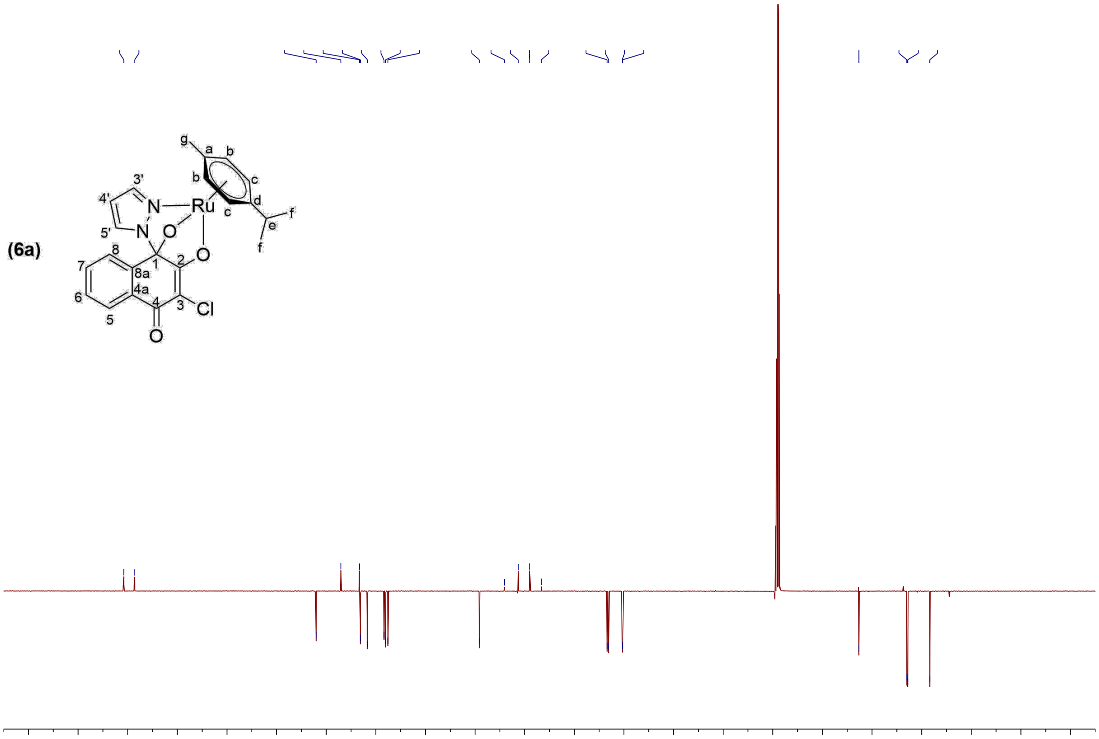

Figure S 20: ^{13}C-NMR (MeOD-d_4, 600.25 MHz) spectrum of (**6a**)

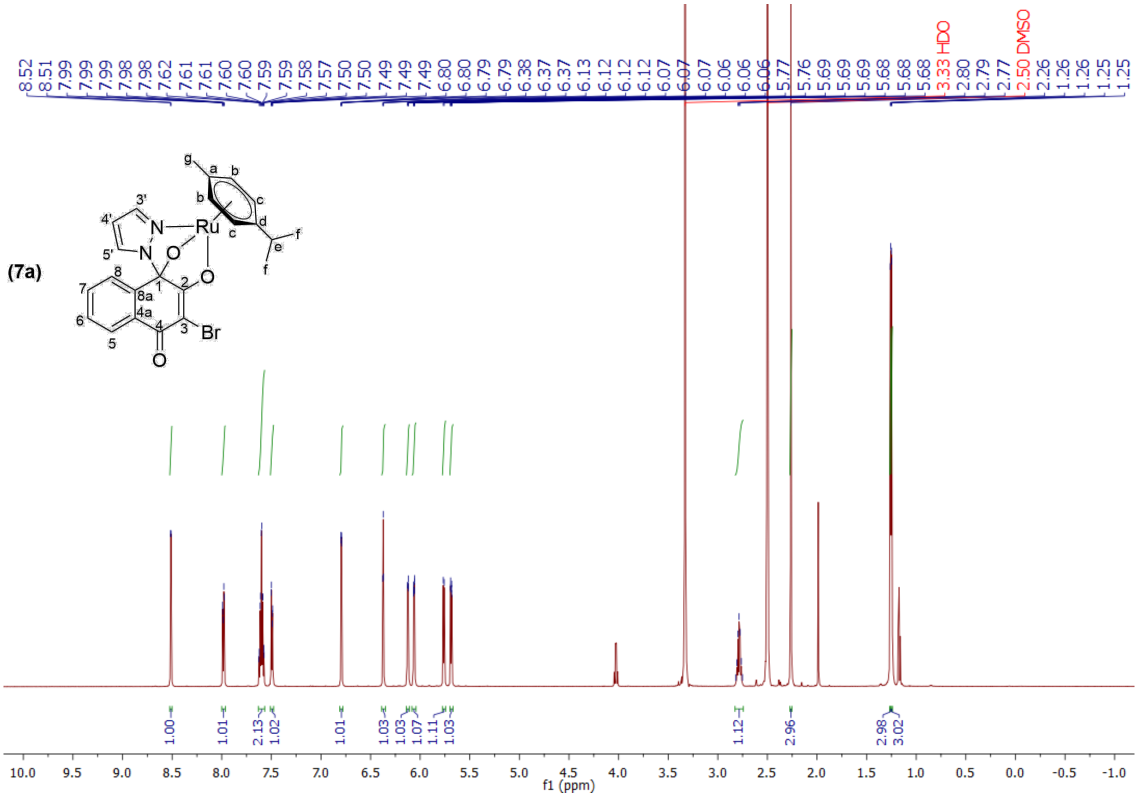

Figure S 21: ^{1}H-NMR (DMSO-d_6, 600.25 MHz) spectrum of (**7a**)

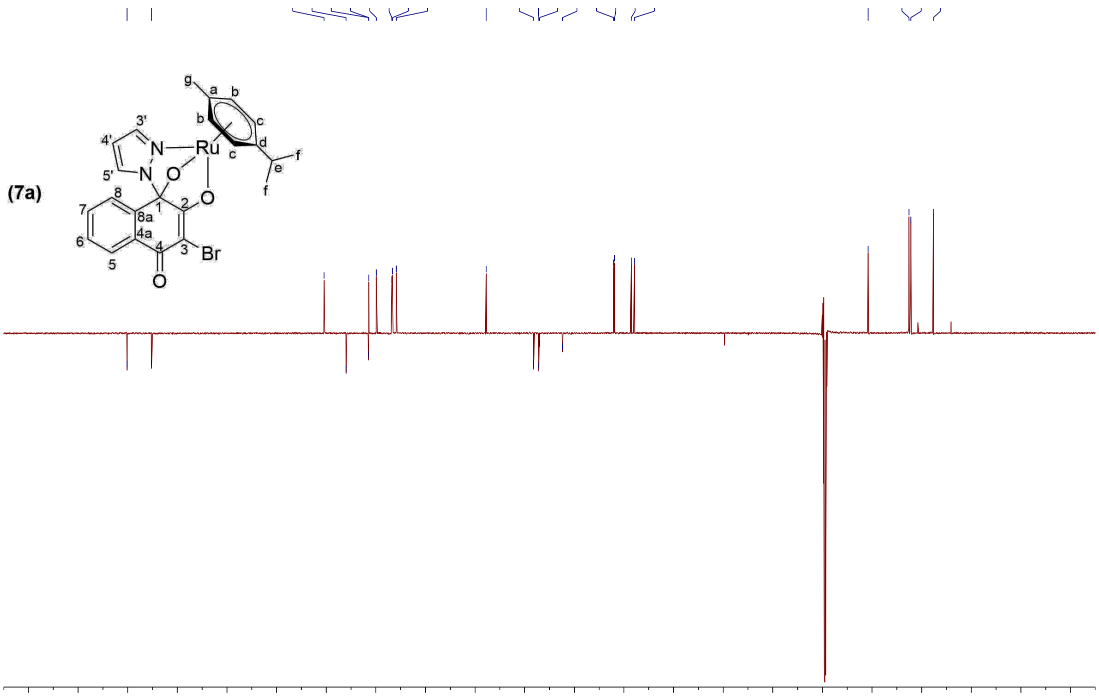

Figure S 22: ^{13}C-NMR (DMSO-d_6, 600.25 MHz) spectrum of (**7a**)

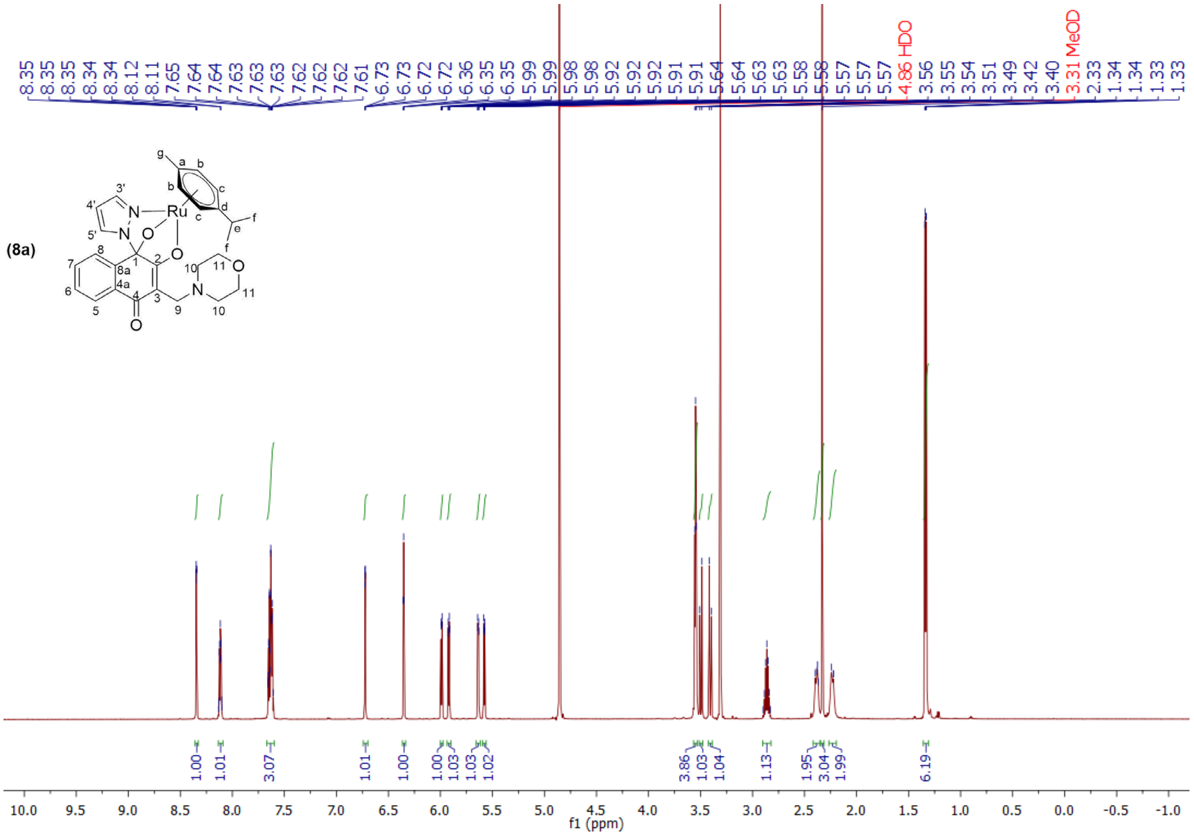

Figure S 23: ^{1}H-NMR (MeOD-d_4, 600.25 MHz) spectrum of (**8a**)

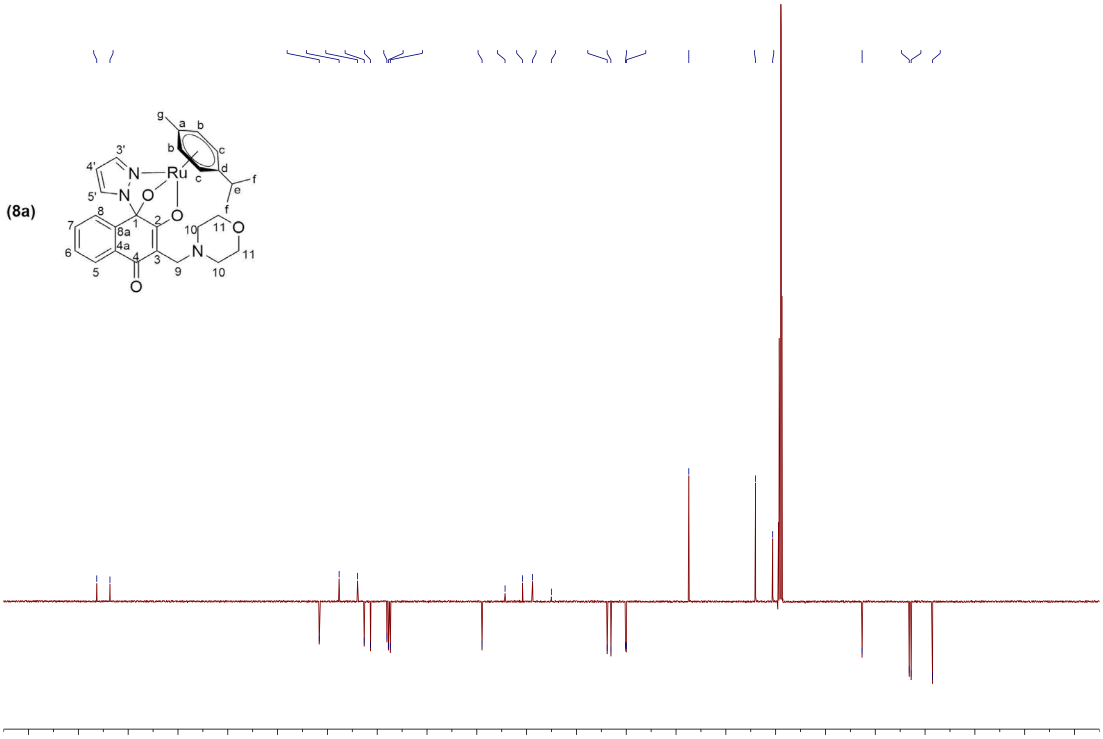

Figure S 24: ^{13}C-NMR (MeOD-d_4, 600.25 MHz) spectrum of (**8a**)

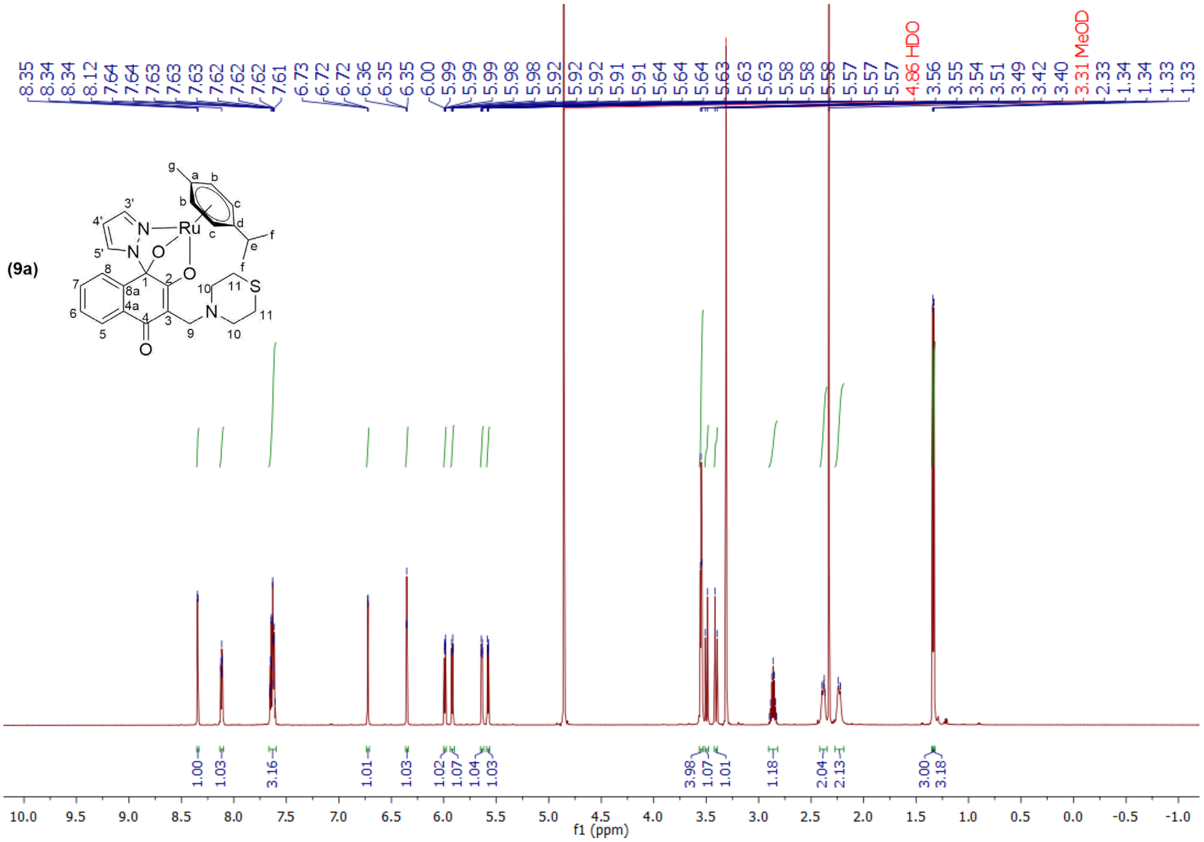

Figure S 25: ^{1}H-NMR (MeOD-d_4, 600.25 MHz) spectrum of (**9a**)

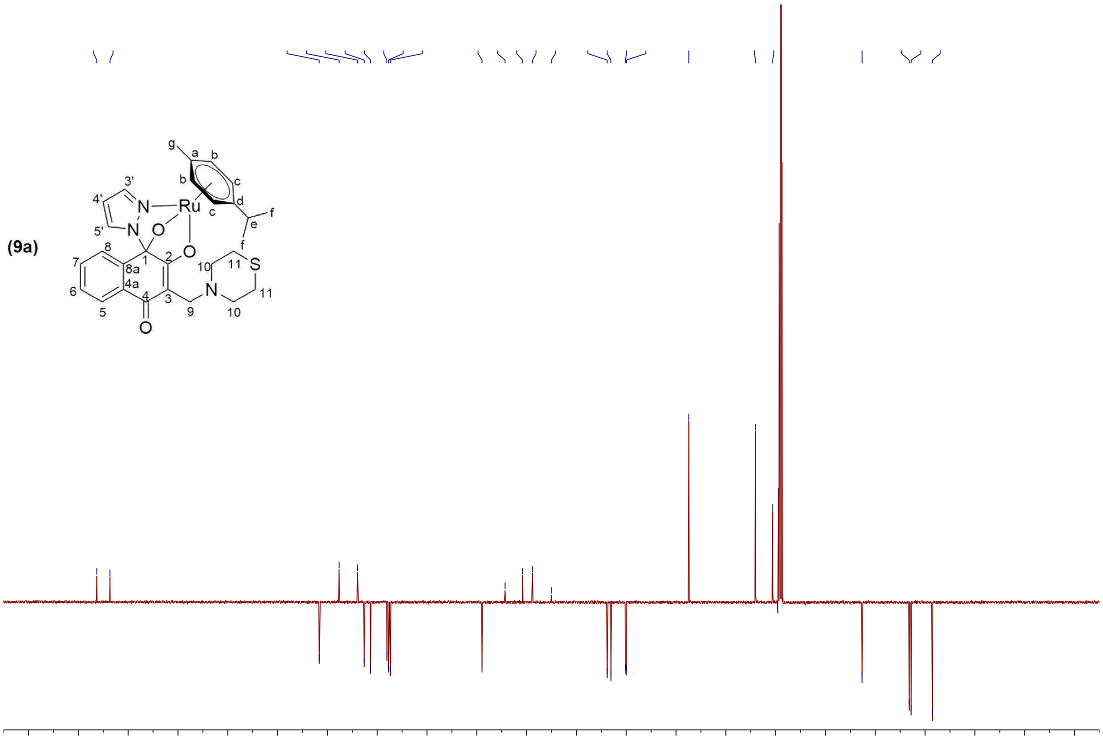

Figure S 26: ^{13}C-NMR (MeOD-d_4, 600.25 MHz) spectrum of (**9a**)

X-ray Analysis

The X-ray intensity data were measured on Bruker D8 Venture diffractometer equipped with multilayer monochromator, Mo and Cu K/α INCOATEC micro focus sealed tube and Oxford cooling system. The structures were solved by either *Direct Methods, Intrinsic Phasing or Patterson Method*. Non-hydrogen atoms were refined with *anisotropic displacement parameters*. Hydrogen atoms were inserted at calculated positions and refined with riding model. The following software was used: *Bruker SAINT software package*[1] using a narrow-frame algorithm for frame integration, *SADABS*[2] for absorption correction, *OLEX2*[3] for structure solution, refinement, molecular diagrams and graphical user-interface, *Shelxle*[4] for refinement and graphical user-interface *SHELXS-2008*[5] for structure solution, *SHELXL-2015*[6] for refinement, *Platon*[7] for symmetry check. Experimental data and CCDC-Codes Experimental data (Available online: http://www.ccdc.cam.ac.uk/conts/retrieving.html) can be found in Table S1. Crystal data, data collection parameters, and structure refinement details are given in Tables S2 to S13. Asymmetric Unit visualized in Figure S 27 to S 33.

Table S 1: Experimental parameter and CCDC-Code.

**Sample**	**Machine**	**Source**	**Temp.**	**Detector Distance**	**Time/ Frame**	**#Frames**	**Frame width**	**CCDC**
	Bruker		[K]	[mm]	[s]		[°]	
**1a**	D8	Mo	130	40	20	587	0.500	2067989
**3a**	D8	Mo	100	40	10	3208	0.360	2067988
**4a**	D8	Mo	150	40	1	1558	0.360	2067993
**5a**	D8	Mo	110	40	20	360	1.000	2067992
**6a**	D8	Mo	100	40	20	3638	0.360	2067994
**7a**	D8	Cu	100	30	5	2913	0.500	2067990
**9a**	D8	Mo	100	40	1	561	0.800	20676991

1a

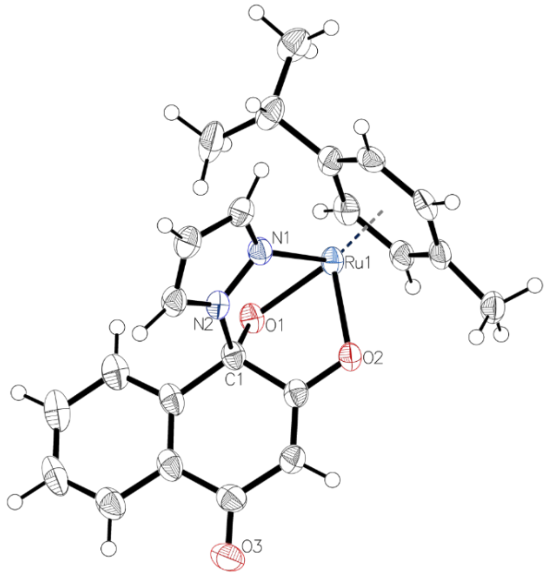

Figure S 27: Crystal structure [**1a**] drawn with 50% displacement ellipsoid. The bond precision for C-C single bonds is 0.0046Å. Squeeze was used. According void contain 86, 23e-.

Table S 2: Sample and crystal data.

Radiation [Å]	MoKα (λ = 0.71073)	Z	2	Measurement method	\f and \w scans
Crystal habit	clear yellow block	a [Å]	8.0395(3)		
Crystal size [mm^3]	0.07 × 0.055 × 0.01	b [Å]	9.8872(5)	Abs. correction type	multiscan
Empirical formula	C23H22N2O3Ru	c [Å]	16.0291(7)	Abs. correction Tmin	0.0628
Formula weight [g/mol]	475.49	α [°]	103.4273(16)	Abs. correction Tmax	0.0916
Temperature [K]	130.0	β [°]	92.9282(15)	Density (calculated) [g/cm^3]	1.398
Crystal system	Triclinic	γ [°]	112.6467(16)	Absorption coefficient [mm^{-1}]	0.718
Space group	P-1	Volume [Å^3]	1129.65(9)	F (000) [e$^-$]	484.0

Table S 3: Data collection and structure refinement.

2Θ range for data collection [°]	4.64 to 50.682	Index ranges		Goodness-of-fit on F^2	1.040
Reflections collected	10426	h	$-8 \leq h \leq 9$	Diff. peak and hole [e$^-$Å^{-3}]	0.52/-0.49
Data / restraints / parameters	4087/0/265	k	$-11 \leq k \leq 11$		
Refinement method	Patterson Methods	l	$-19 \leq l \leq 19$	Function minimized	Σ w (F$_o^2$ - F$_c^2$)^2
		all data	R1 = 0.0369, wR2 = 0.0685	Weighting scheme	where
		I>2σ(I)	R1 = 0.0305, wR2 = 0.0658	w=1/[σ^2(Fo^2) + (0.0278P)^2 + 0.3665P]	P=(F$_o^2$+2F$_c^2$)/3

3a

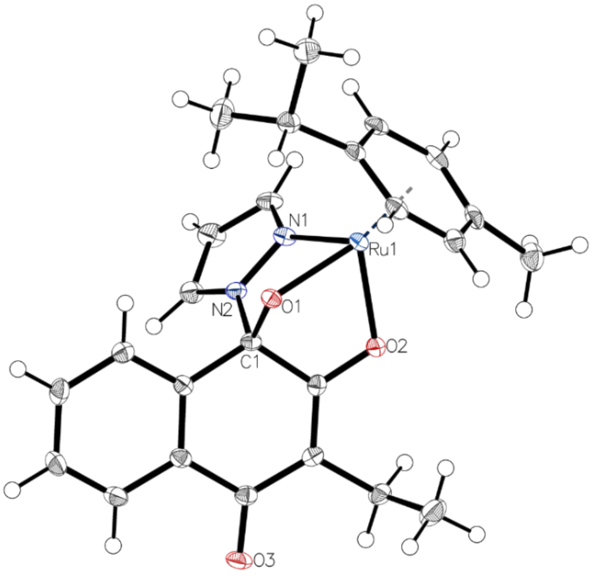

Figure S 28: Crystal structure [**3a**] drawn with 50% displacement ellipsoid. The bond precision for C-C single bonds is 0.0040Å

Table S 4: Sample and crystal data.

Radiation [Å]	MoKα (λ = 0.71073)	Z	4	Measurement method	\f and \w scans
Crystal habit	clear yellow block	a [Å]	9.8420(4)		
Crystal size [mm^3]	0.09 × 0.08 × 0.05	b [Å]	16.0482(8)	Abs. correction type	multiscan
Empirical formula	C25H26N2O3Ru	c [Å]	13.6389(5)	Abs. correction Tmin	0.2338
Formula weight [g/mol]	503.55	α [°]	90	Abs. correction Tmax	0.2650
Temperature [K]	100.0	β [°]	95.3229(16)	Density (calculated) [g/cm^3]	1.559
Crystal system	Monoclinic	γ [°]	90	Absorption coefficient [mm^{-1}]	0.761
Space group	P21/n	Volume [$Å^3$]	2144.93(16)	F (000) [e^-]	1032.0

Table S 5: Data collection and structure refinement.

2Θ range for data collection [°]	3.928 to 50.7	Index ranges		Goodness-of-fit on F^2	1.079
Reflections collected	67632	h	$-11 \le h \le 11$	Diff. peak and hole [$e^-Å^{-3}$]	1.75/-0.48
Data / restraints / parameters	3928/0/284	k	$-19 \le k \le 19$		
Refinement method	Direct Methods	l	$-16 \le l \le 16$	Function minimized	Σ w $(F_o^2 - F_c^2)^2$
		all data	R1 = 0.0292, wR2 = 0.0666	Weighting scheme	where
		$I>2\sigma(I)$	R1 = 0.0264, wR2 = 0.0656	$w=1/[\sigma^2(Fo^2) + (0.0200P)^2 + 5.4208P]$	$P=(F_o^2+2F_c^2)/3$

4a

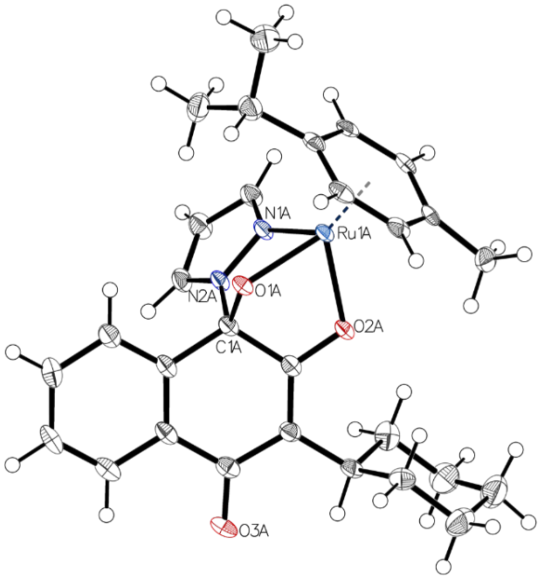

Figure S 29: Crystal structure [**4a**] drawn with 50% displacement ellipsoid. The bond precision for C-C single bonds is 0.0040Å. Solvent and second independent molecule omitted for clarity.

Table S 6: Sample and crystal data.

Radiation [Å]	MoKα (λ = 0.71073)	Z	4	Measurement method	\f and \w scans
Crystal habit	clear yellow block	a [Å]	12.8106(6)		
Crystal size [mm^3]	0.416 × 0.162 × 0.141	b [Å]	13.3834(5)	Abs. correction type	multiscan
Empirical formula	C29.5H33ClN2O3Ru	c [Å]	16.5381(6)	Abs. correction Tmin	0.4249
Formula weight [g/mol]	600.10	α [°]	99.811(2)	Abs. correction Tmax	0.4954
Temperature [K]	150.0	β [°]	97.1353(17)	Density (calculated) [g/cm^3]	1.477
Crystal system	Triclinic	γ [°]	101.6508(17)	Absorption coefficient [mm^{-1}]	0.713
Space group	P-1	Volume [Å^3]	2699.3(2)	F (000) [e$^-$]	1236.0

Table S 7: Data collection and structure refinement.

2Θ range for data collection [°]	4.016 to 52.044	Index ranges		Goodness-of-fit on F^2	1.062
Reflections collected	52564	h	$-15 \leq h \leq 15$	Diff. peak and hole [e$^-$Å^{-3}]	1.72/-1.10
Data / restraints / parameters	10627/0/664	k	$-16 \leq k \leq 16$		
Refinement method	Direct Methods	l	$-20 \leq l \leq 20$	Function minimized	Σ w $(F_o^2 - F_c^2)^2$
		all data	R1 = 0.0418, wR2 = 0.0895	Weighting scheme	where
		I>2σ(I)	R1 = 0.0327, wR2 = 0.0854	$w=1/[\sigma^2(Fo^2) + (0.0516P)^2 +1.2684P]$	$P=(F_o^2+2F_c^2)/3$

5a

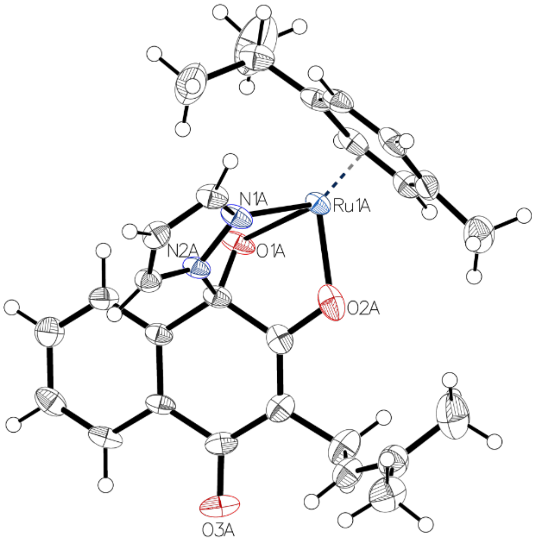

Figure S 30: Crystal structure [**5a**] drawn with 50% displacement ellipsoid. The bond precision for C-C single bonds is 0.0040Å. Second independent molecule omitted for clarity. Shelx Compatible Restraints and Constraints are used, because of high unstable atoms. Additionally AFIX 31 was used for stabilizing.

Table S 8

Radiation [Å]	MoKα (λ = 0.71073)	Z	8	Measurement method	\f and \w scans
Crystal habit	clear yellow block	a [Å]	13.7082(3)		
Crystal size [mm^3]	0.13 × 0.1 × 0.06	b [Å]	20.9715(8)	Abs. correction type	multiscan
Empirical formula	C28H28.94N2O3Ru	c [Å]	17.7788(6)	Abs. correction Tmin	0.6937
Formula weight [g/mol]	542.54	α [°]	90	Abs. correction Tmax	0.7460
Temperature [K]	100.0	β [°]	105.2829(12)	Density (calculated) [g/cm^3]	1.462
Crystal system	Monoclinic	γ [°]	90	Absorption coefficient [mm^{-1}]	0.668
Space group	P21/n	Volume [$Å^3$]	4930.2(3)	F (000) [e^-]	2232.0

Table S 9

2Θ range for data collection [°]	3.878 to 60.164	Index ranges		Goodness-of-fit on F^2	1.064
Reflections collected	144132	h	$-19 \leq h \leq 19$	Diff. peak and hole [$e^-Å^{-3}$]	0.65/-1.21
Data / restraints / parameters	14476/182/740	k	$-29 \leq k \leq 29$		
Refinement method	Direct Methods	l	$-25 \leq l \leq 24$	Function minimized	Σ w $(F_o^2 - F_c^2)^2$
		all data	R1 = 0.0767, wR2 = 0.1077	Weighting scheme	where
		I>2σ(I)	R1 = 0.0423, wR2 = 0.0966	$w=1/[\sigma^2(Fo^2) + (0.0442P)^2 + 2.8882P]$	$P=(F_o^2+2F_c^2)/3$

6a

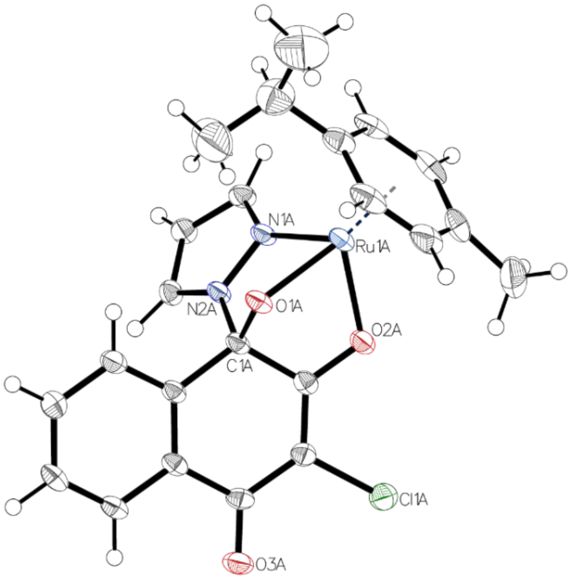

Figure S 31: Crystal structure [**6a**] drawn with 50% displacement ellipsoid. The bond precision for C-C single bonds is 0.0051Å. Solvent and second independent molecule omitted for clarity.

Table S 10: Sample and crystal data.

Radiation [Å]	MoKα (λ = 0.71073)	Z	8	Measurement method	\f and \w scans
Crystal habit	clear yellow needle	a [Å]	13.6374(5)		
Crystal size [mm^3]	0.06 × 0.04 × 0.03	b [Å]	19.7452(8)	Abs. correction type	multiscan
Empirical formula	C23.5H22Cl2N2O3Ru	c [Å]	17.2012(8)	Abs. correction Tmin	0.4669
Formula weight [g/mol]	522.40	α [°]	90	Abs. correction Tmax	0.5642
Temperature [K]	100.0	β [°]	104.952(2)	Density (calculated) [g/cm^3]	1.640
Crystal system	Monoclinic	γ [°]	90	Absorption coefficient [mm^{-1}]	0.968
Space group	P21/n	Volume [Å^3]	4475.0(3)	F (000) [e$^-$]	2232.0

Table S 11: Data collection and structure refinement.

2Θ range for data collection [°]	3.988 to 60.068	Index ranges		Goodness-of-fit on F^2	1.065
Reflections collected	214444	h	$-19 \leq h \leq 19$	Diff. peak and hole [e$^-$Å^{-3}]	1.79/-1.93
Data / restraints / parameters	13099/668/580	k	$-27 \leq k \leq 27$		
Refinement method	Direct Methods	l	$-24 \leq l \leq 24$	Function minimized	Σ w $(F_o^2 - F_c^2)^2$
		all data	R1 = 0.0569, wR2 = 0.1106	Weighting scheme	where
		I>2σ(I)	R1 = 0.0468, wR2 = 0.1069	$w=1/[\sigma^2(Fo^2) + (0.0298P)^2 + 15.0024P]$	$P=(F_o^2+2F_c^2)/3$

7a

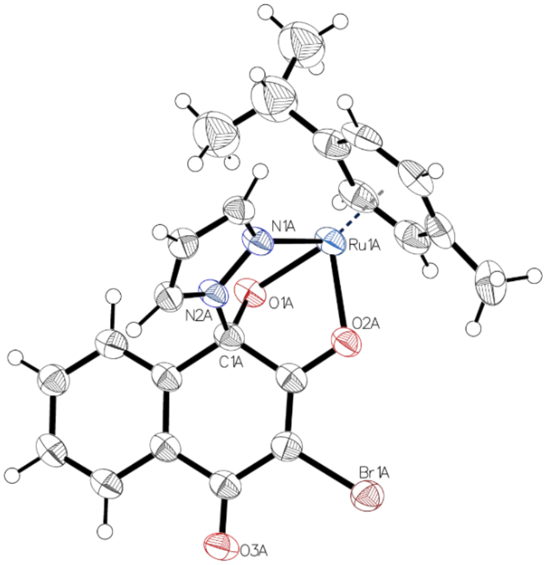

Figure S 32: Crystal structure [**7a**] drawn with 50% displacement ellipsoid. The bond precision for C-C single bonds is 0.0058Å. Solvent and second independent molecule omitted for clarity.

Table S 12: Sample and crystal data.

Radiation [Å]	CuKα (λ = 1.54178)	Z	8	Measurement method	\f and \w scans
Crystal habit	clear yellow needle	a [Å]	13.6698(5)		
Crystal size [mm^3]	0.18 × 0.03 × 0.03	b [Å]	19.8725(8)	Abs. correction type	multiscan
Empirical formula	C23.5H22BrClN2O3Ru	c [Å]	17.1917(7)	Abs. correction Tmin	0.0485
Formula weight [g/mol]	596.86	α [°]	90	Abs. correction Tmax	0.1665
Temperature [K]	100.0	β [°]	104.9430(10)	Density (calculated) [g/cm^3]	1.757
Crystal system	Monoclinic	γ [°]	90	Absorption coefficient [mm^{-1}]	9.029
Space group	P21/n	Volume [Å^3]	4512.2(3)	F (000) [e$^-$]	2376.0

Table S 13: Data collection and structure refinement.

2Θ range for data collection [°]	6.936 to 137.002	Index ranges		Goodness-of-fit on F^2	1.102
Reflections collected	50340	h	$-16 \leq h \leq 16$	Diff. peak and hole [e$^-$Å^{-3}]	1.40/-0.84
Data / restraints / parameters	8271/4/559	k	$-23 \leq k \leq 23$		
Refinement method	Intrinsic Phasing	l	$-20 \leq l \leq 18$	Function minimized	Σ w $(F_o^2 - F_c^2)^2$
		all data	R1 = 0.0341, wR2 = 0.0855	Weighting scheme	where
		I>2σ(I)	R1 = 0.0333, wR2 = 0.0848	w=1/[σ^2(Fo^2) + (0.0351P)^2 + 7.8994P]	P=$(F_o^2+2F_c^2)/3$

9a

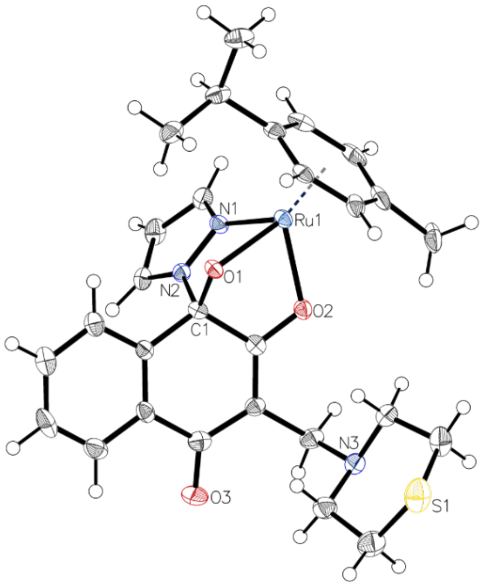

Figure S 33: Crystal structure [**9a**] drawn with 50% displacement ellipsoid. The bond precision for C-C single bonds is 0.0042Å. Solvent omitted for clarity. Squeeze was used. According void contain 185, 46e-.

Table S 14: Sample and crystal data.

Radiation [Å]	MoKα (λ = 0.71073)	Z	8	Measurement method	\f and \w scans
Crystal habit	clear yellow block	a [Å]	14.4763(4)		
Crystal size [mm^3]	0.184 × 0.14 × 0.074	b [Å]	17.7996(5)	Abs. correction type	multiscan
Empirical formula	C28H31.5N3O3.5RuS	c [Å]	24.4151(6)	Abs. correction Tmin	0.2260
Formula weight [g/mol]	599.19	α [°]	90	Abs. correction Tmax	0.2574
Temperature [K]	100.0	β [°]	98.5242(9)	Density (calculated) [g/cm^3]	1.279
Crystal system	Monoclinic	γ [°]	90	Absorption coefficient [mm^{-1}]	0.603
Space group	C2/c	Volume [$Å^3$]	6221.6(3)	F (000) [e^-]	2468.0

Table S 15: Data collection and structure refinement.

2Θ range for data collection [°]	4.576 to 50.694	Index ranges		Goodness-of-fit on F^2	1.038
Reflections collected	52459	h	$-17 \leq h \leq 17$	Diff. peak and hole [$e^-Å^{-3}$]	0.49/-0.35
Data / restraints / parameters	5699/0/336	k	$-21 \leq k \leq 21$		
Refinement method	Patterson Method	l	$-29 \leq l \leq 27$	Function minimized	Σ w $(F_o^2 - F_c^2)^2$
		all data	R1 = 0.0479, wR2 = 0.0788	Weighting scheme	where
		I>2σ(I)	R1 = 0.0349, wR2 = 0.0734	$w=1/[\sigma^2(Fo^2) + (0.0274P)^2 + 5.7123P]$	$P=(F_o^2+2F_c^2)/3$

Stability measurements

UV-Vis spectra

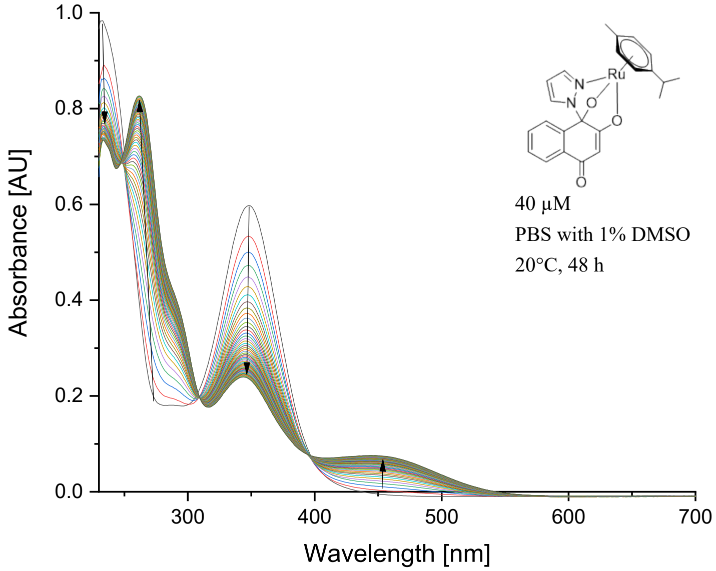

Figure S 34: UV-Vis spectrum of compound **1a**

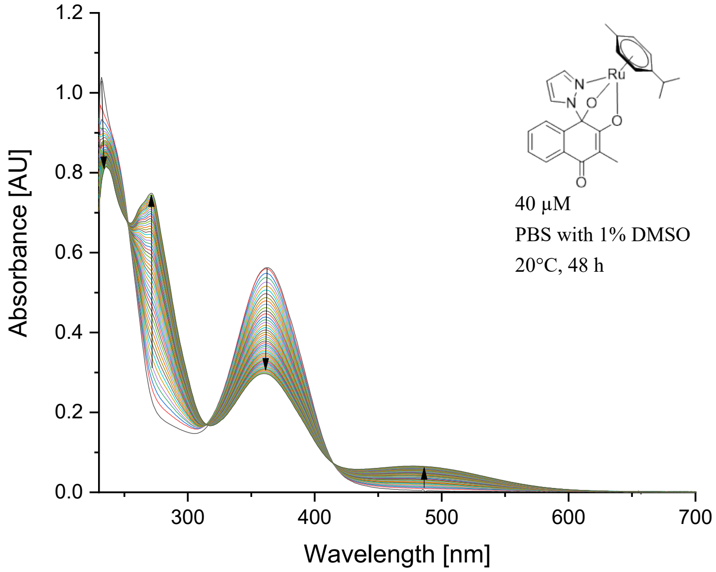

Figure S 35: UV-Vis spectrum of compound **2a**

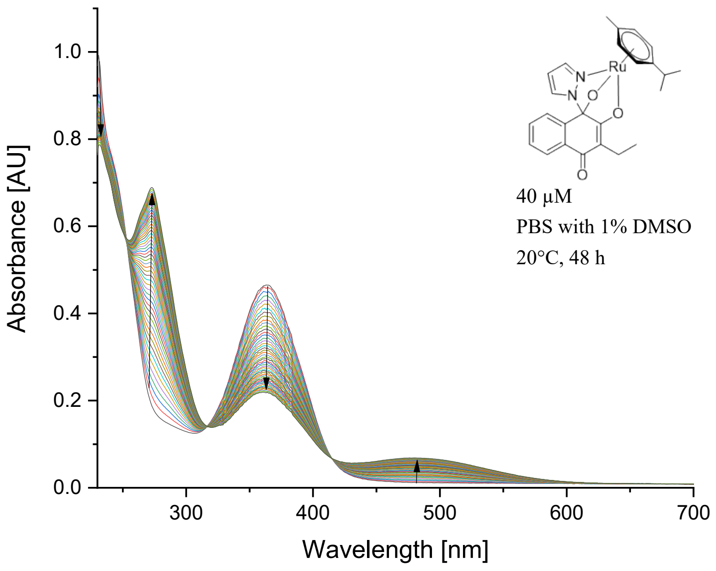

Figure S 36: UV-Vis spectrum of compound **3a**

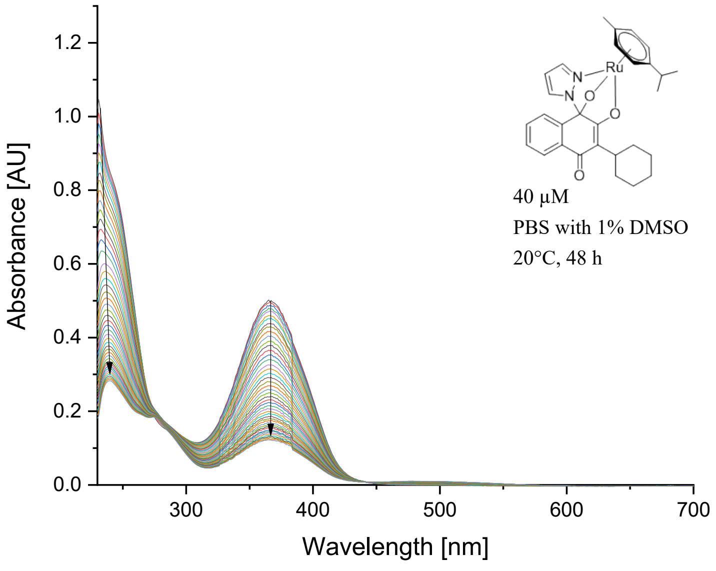

Figure S 37: UV-Vis spectrum of compound **4a**

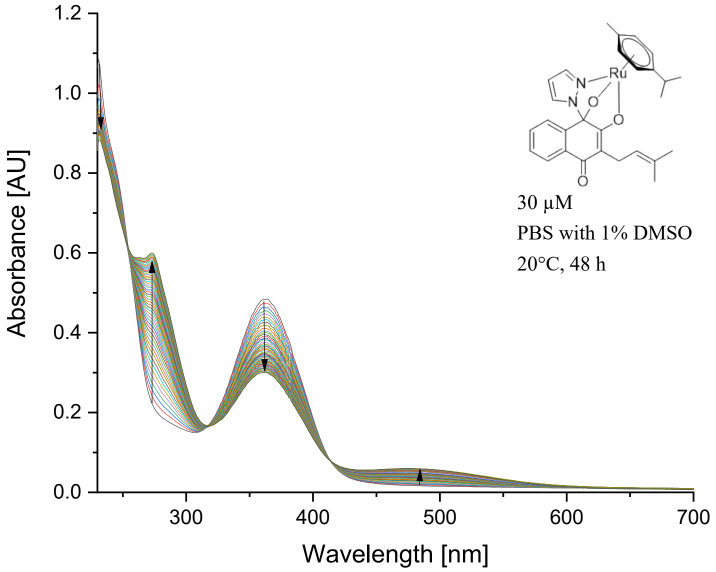

Figure S 38: UV-Vis spectrum of compound **5a**

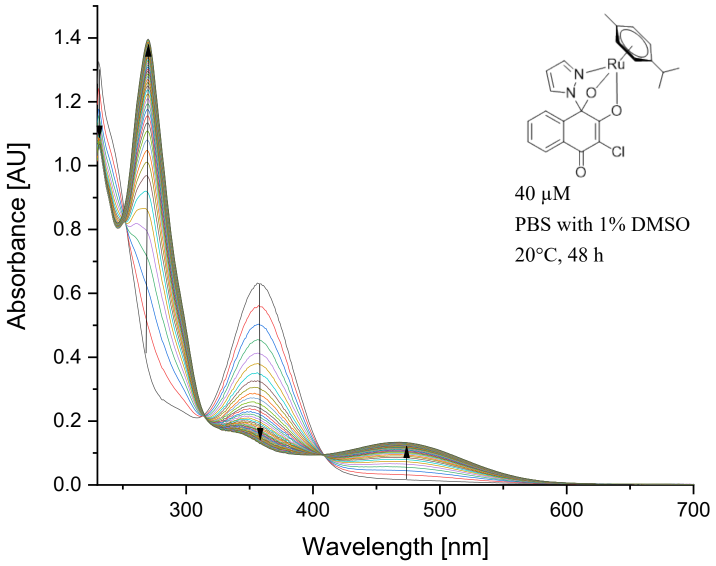

Figure S 39: UV-Vis spectrum of compound **6a**

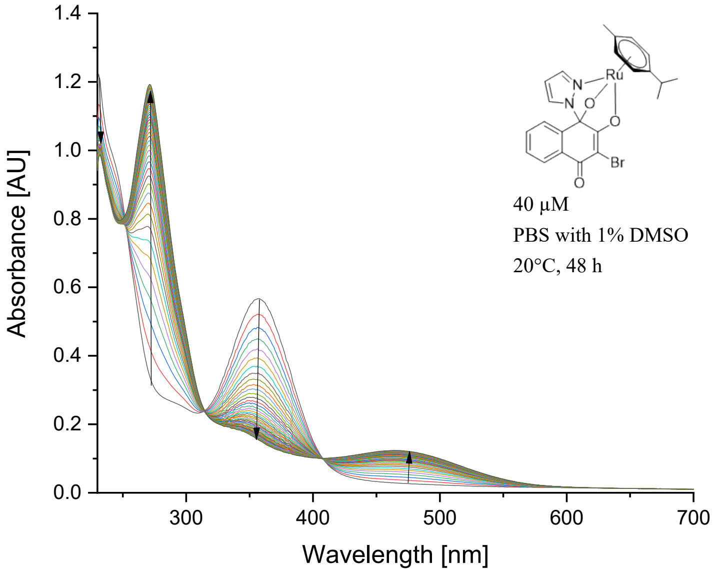

Figure S 40: UV-Vis spectrum of compound **7a**

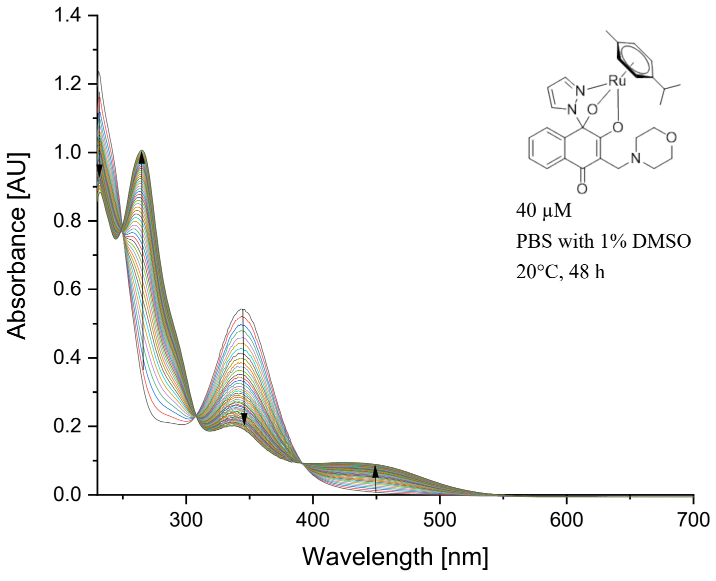

Figure S 41: UV-Vis spectrum of compound **8a**

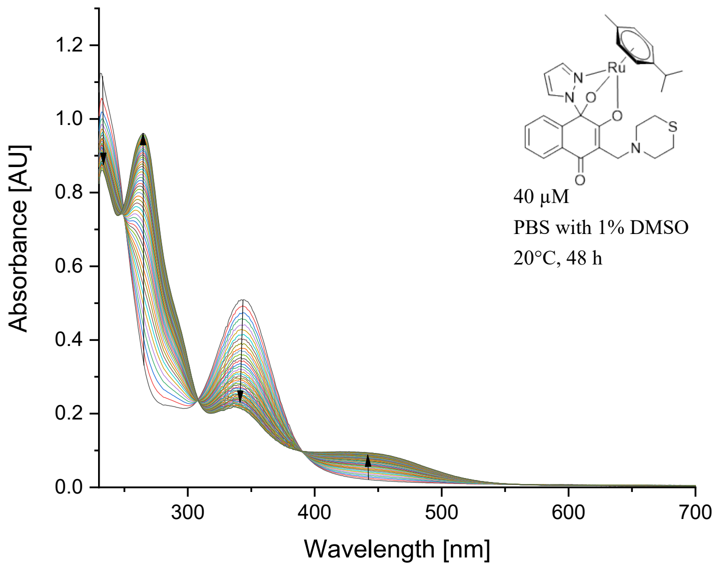

Figure S 42: UV-Vis spectrum of compound **9a**

HPLC-MS

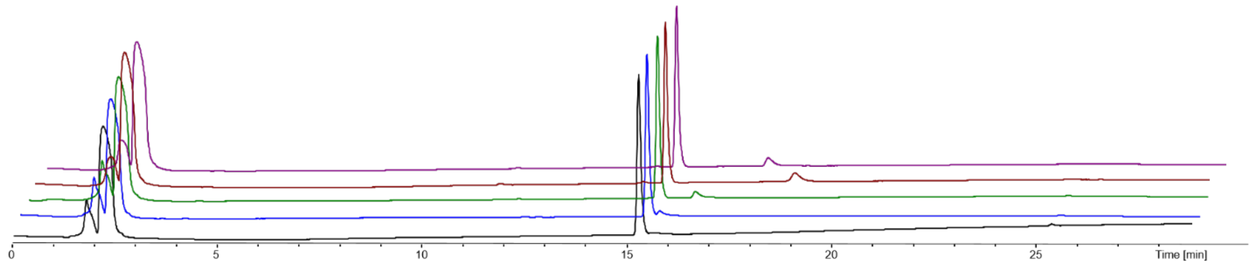

Figure S 43: HPLC chromatogram of compound **1a** after 0, 1, 2, 3, 4, 24 h (from front to back; conditions see experimental section).

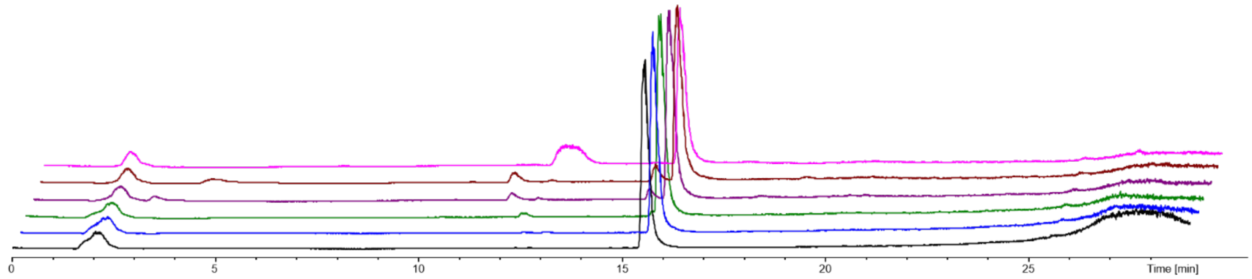

Figure S 44: HPLC-MS chromatogram of compound **1a** after 0, 1, 2, 3, 4, 24 h (from front to back; conditions see experimental section).

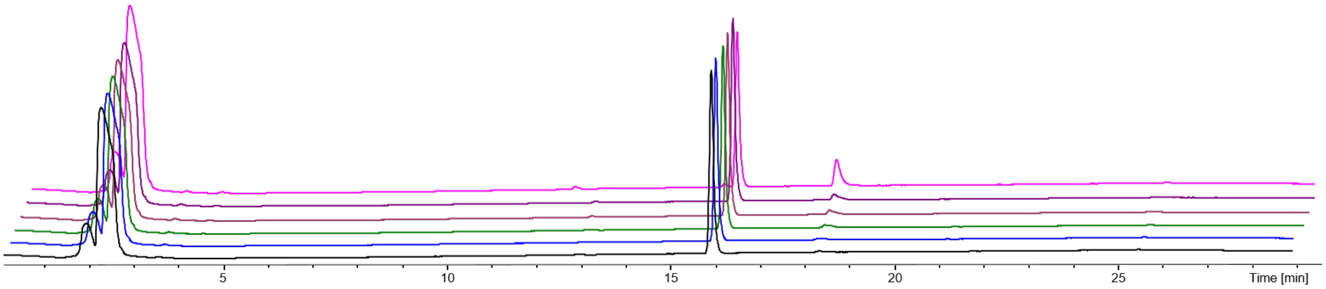

Figure S 45: HPLC chromatogram of compound **2a** after 0, 1, 2, 3, 4, 24 h (from front to back; conditions see experimental section).

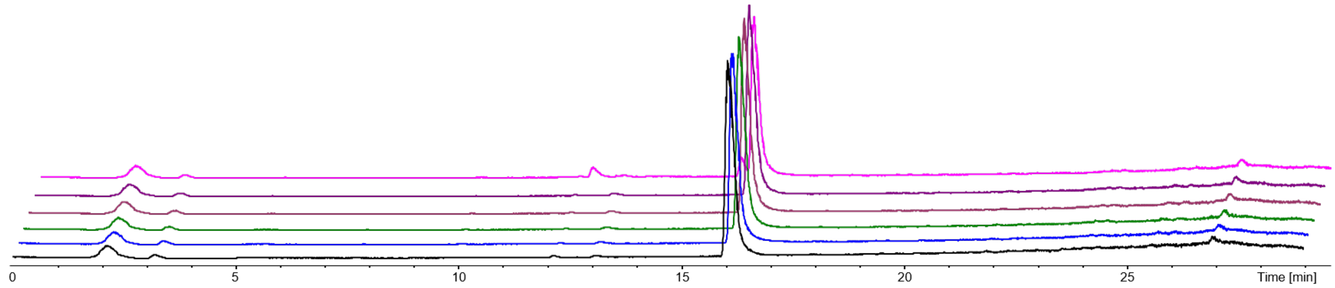

Figure S 46: HPLC-MS chromatogram of compound **2a** after 0, 1, 2, 3, 4, 24 h (from front to back; conditions see experimental section).

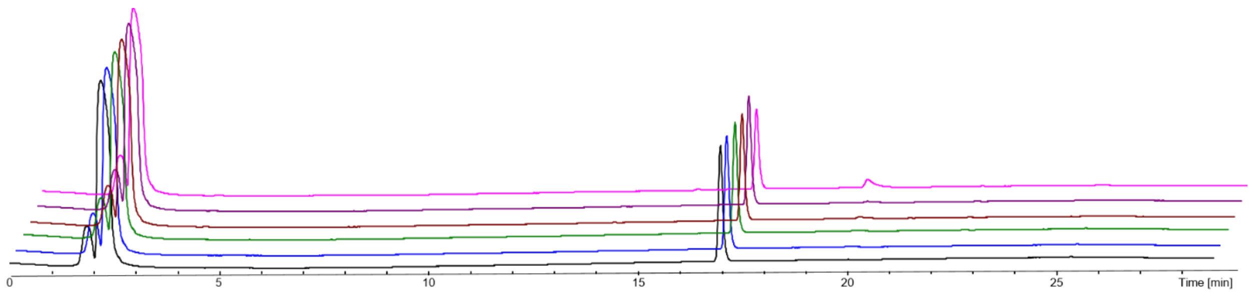

Figure S 47: HPLC chromatogram of compound **3a** after 0, 1, 2, 3, 4, 24 h (from front to back; conditions see experimental section).

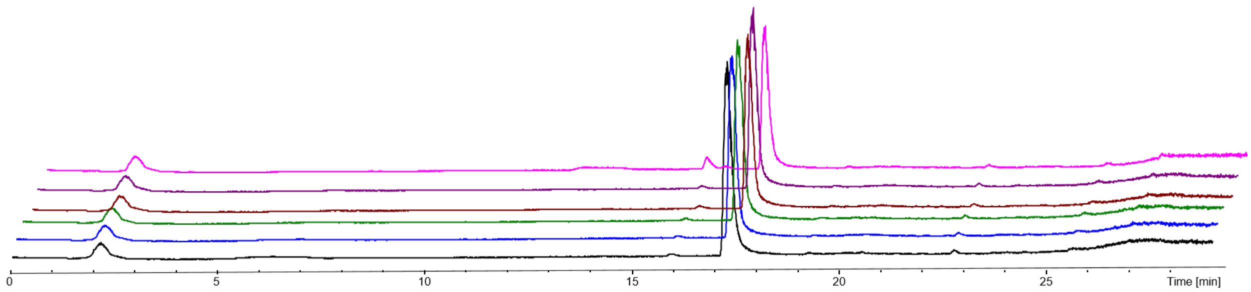

Figure S 48: HPLC-MS chromatogram of compound **3a** after 0, 1, 2, 3, 4, 24 h (from front to back; conditions see experimental section).

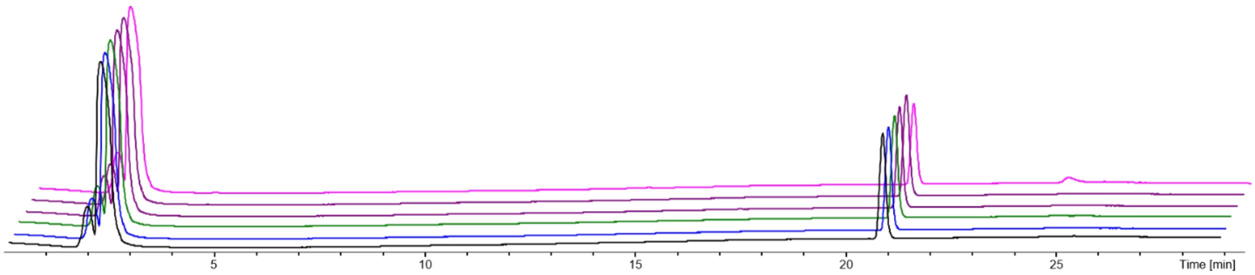

Figure S 49: HPLC chromatogram of compound **4a** after 0, 1, 2, 3, 4, 24 h (from front to back; conditions see experimental section).

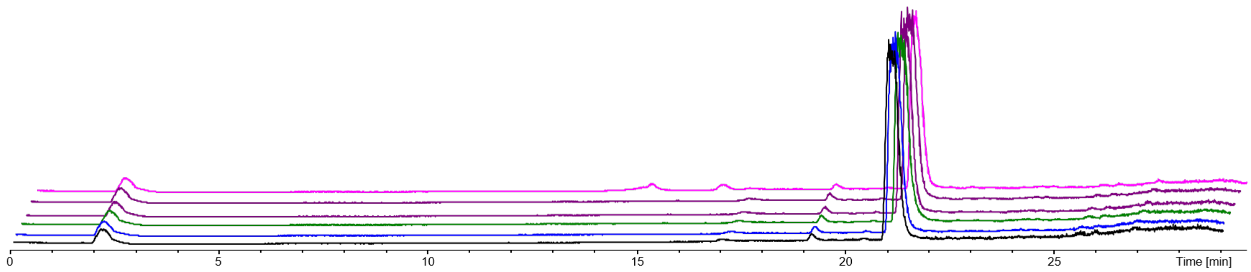

Figure S 50: HPLC-MS chromatogram of compound **4a** after 0, 1, 2, 3, 4, 24 h (from front to back; conditions see experimental section).

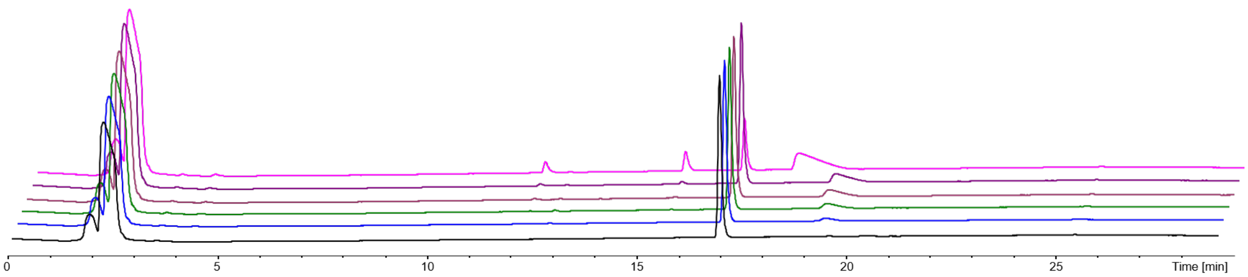

Figure S 51: HPLC chromatogram of compound **6a** after 0, 1, 2, 3, 4, 24 h (from front to back; conditions see experimental section).

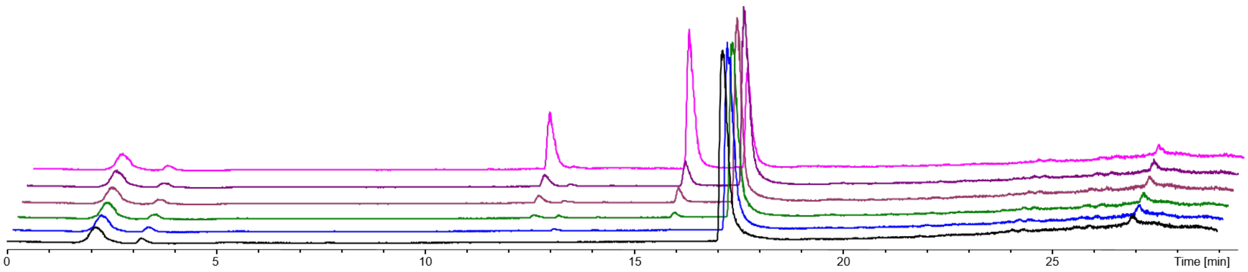

Figure S 52: HPLC-MS chromatogram of compound **6a** after 0, 1, 2, 3, 4, 24 h (from front to back; conditions see experimental section).

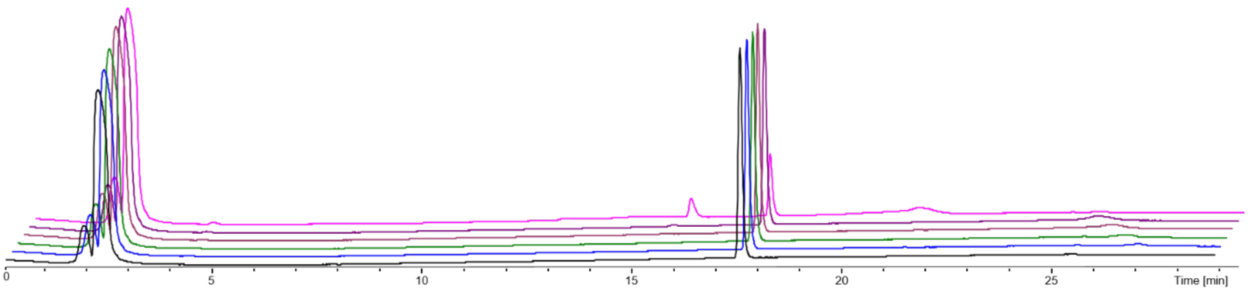

Figure S 53: HPLC chromatogram of compound **7a** after 0, 1, 2, 3, 4, 24 h (from front to back; conditions see experimental section).

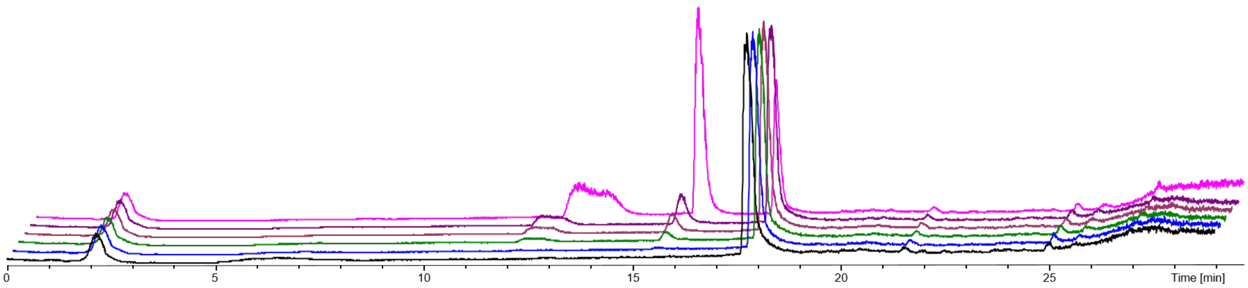

Figure S 54: HPLC-MS chromatogram of compound **7a** after 0, 1, 2, 3, 4, 24 h (from front to back; conditions see experimental section).

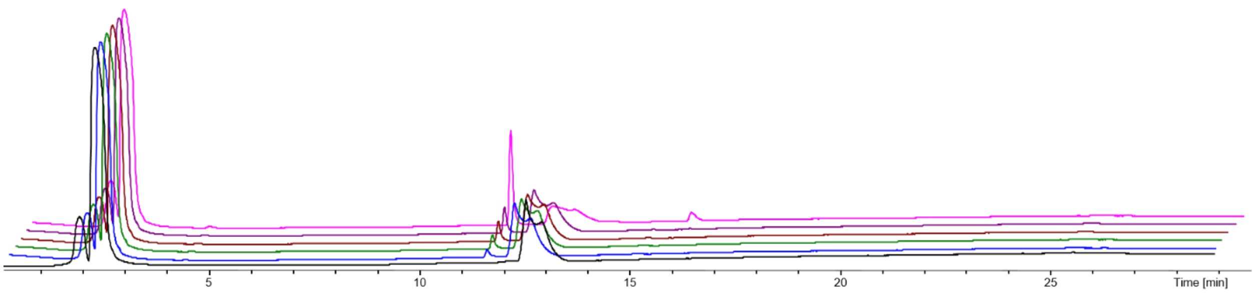

Figure S 55: HPLC chromatogram of compound **8a** after 0, 1, 2, 3, 4, 24 h (from front to back; conditions see experimental section).

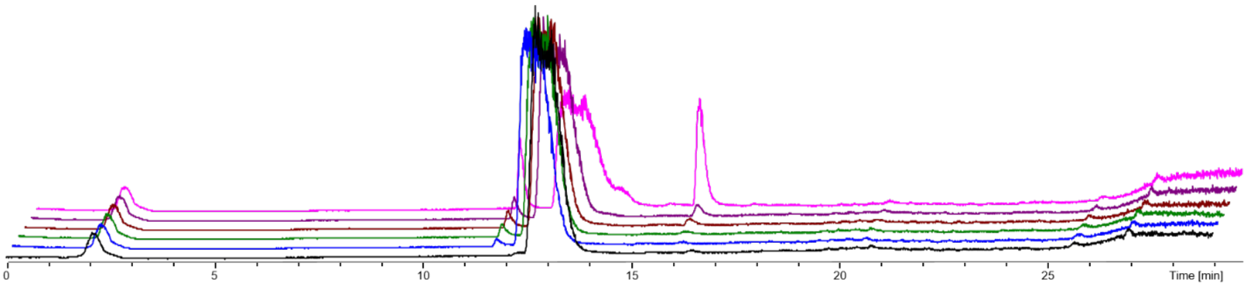

Figure S 56: HPLC-MS chromatogram of compound **8a** after 0, 1, 2, 3, 4, 24 h (from front to back; conditions see experimental section).

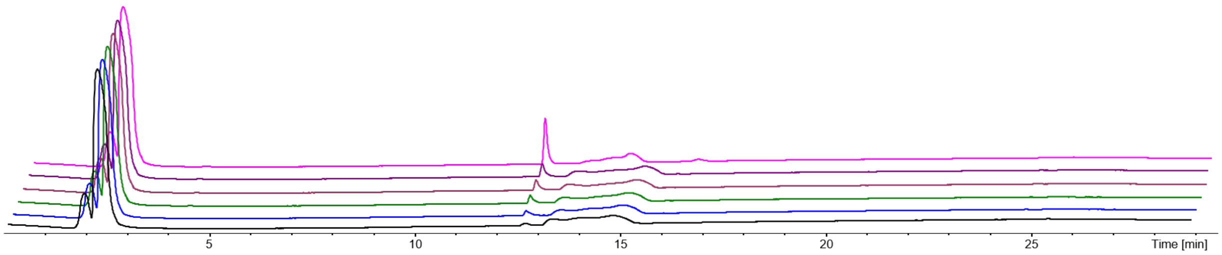

Figure S 57: HPLC chromatogram of compound **9a** after 0, 1, 2, 3, 4, 24 h (from front to back; conditions see experimental section).

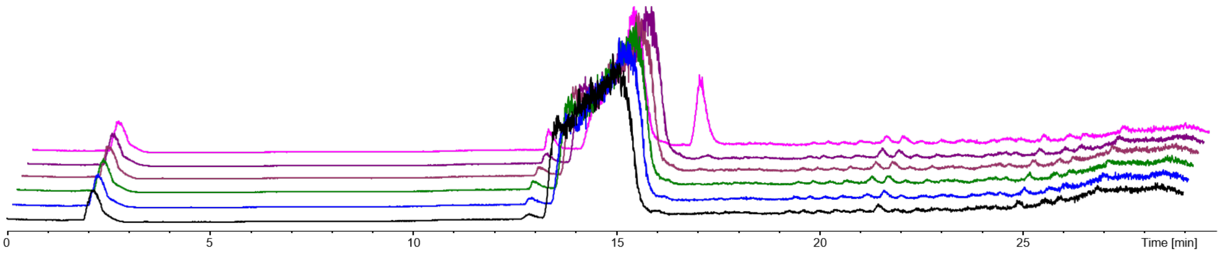

Figure S 58: HPLC-MS chromatogram of compound **9a** after 0, 1, 2, 3, 4, 24 h (from front to back; conditions see experimental section).

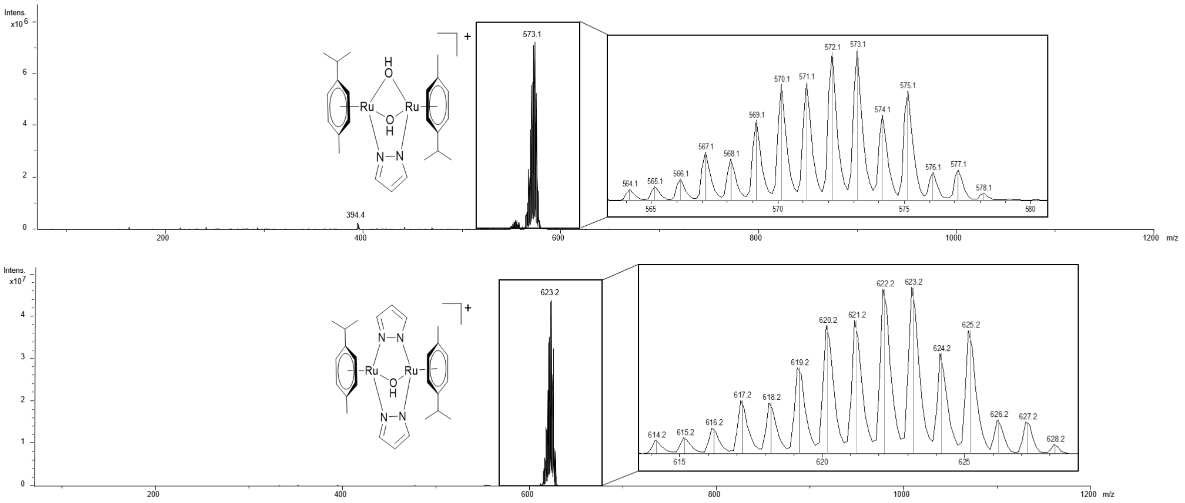

Figure S 59: Mass spectra of [((*p*-cymene)Ru)$_2$(μ-OH)(μ-pyrazolate)$_2$]$^+$ (*m/z* = 623.2) and [((*p*-cymene)Ru)$_2$(μ-OH)$_2$(μ-pyrazolate)]$^+$ (*m/z* = 572.1).

Amino acid incubations studies

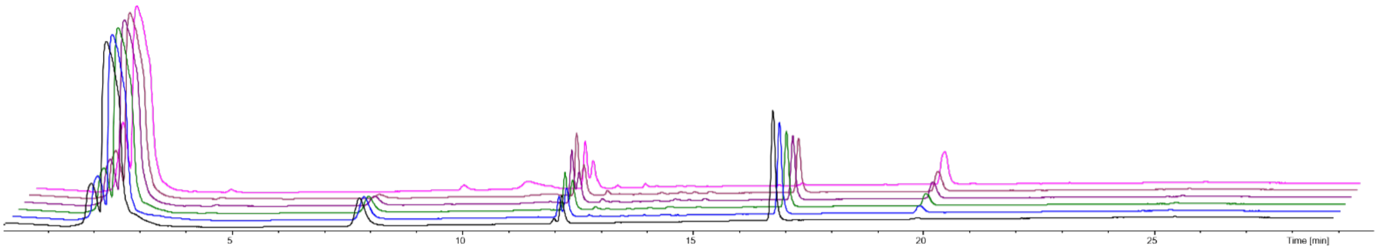

Figure S 60: HPLC chromatogram of compound **3a** after 0, 1, 2, 3, 4, 24 h (from front to back; conditions see experimental section).

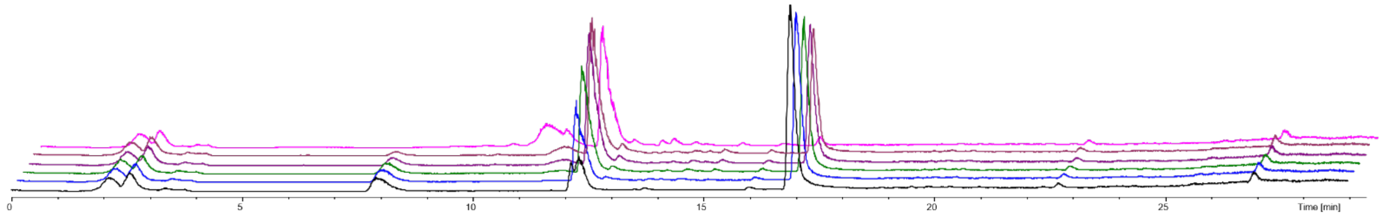

Figure S 61: HPLC-MS chromatogram of compound **3a** after 0, 1, 2, 3, 4, 24 h (from front to back; conditions see experimental section).

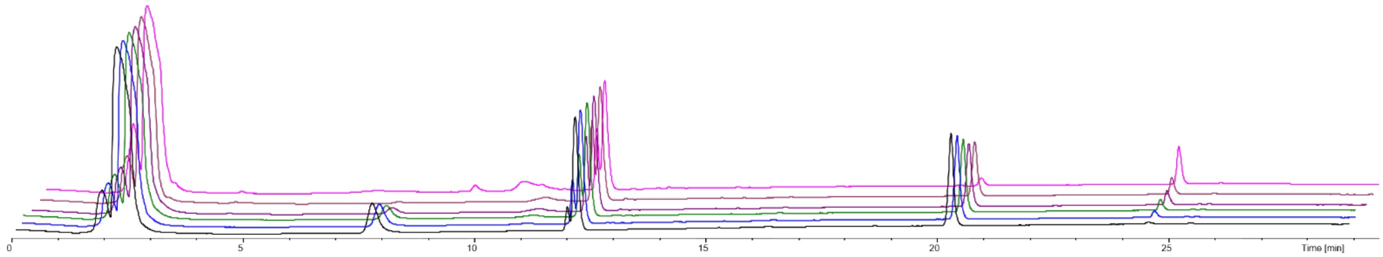

Figure S 62: HPLC chromatogram of compound **4a** after 0, 1, 2, 3, 4, 24 h (from front to back; conditions see experimental section).

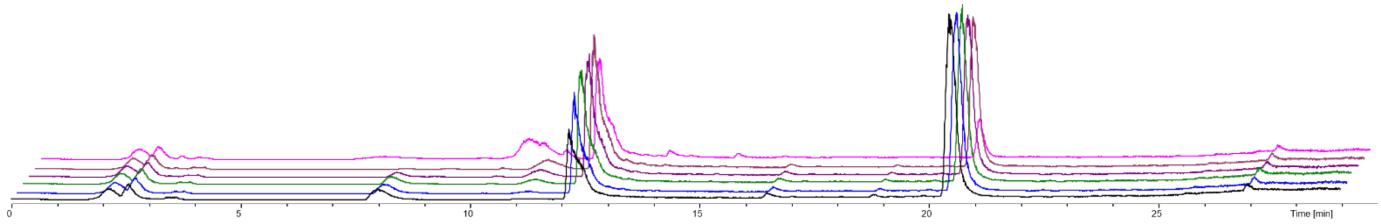

Figure S 63: HPLC-MS chromatogram of compound **4a** after 0, 1, 2, 3, 4, 24 h (from front to back; conditions see experimental section).

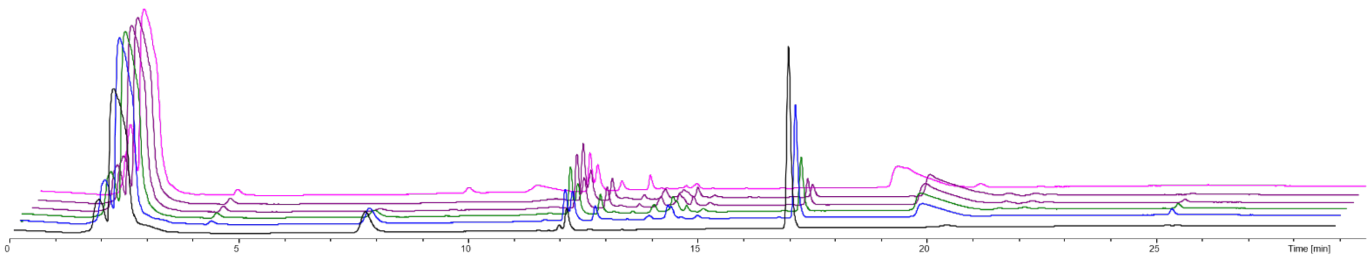

Figure S 64: HPLC chromatogram of compound **6a** after 0, 1, 2, 3, 4, 24 h (from front to back; conditions see experimental section).

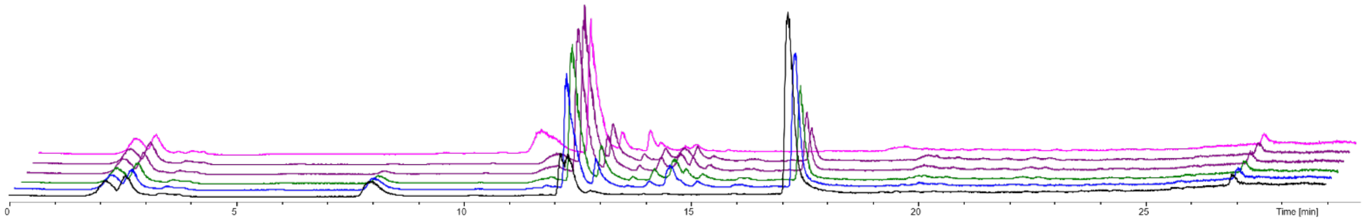

Figure S 65: HPLC-MS chromatogram of compound **6a** after 0, 1, 2, 3, 4, 24 h (from front to back; conditions see experimental section).

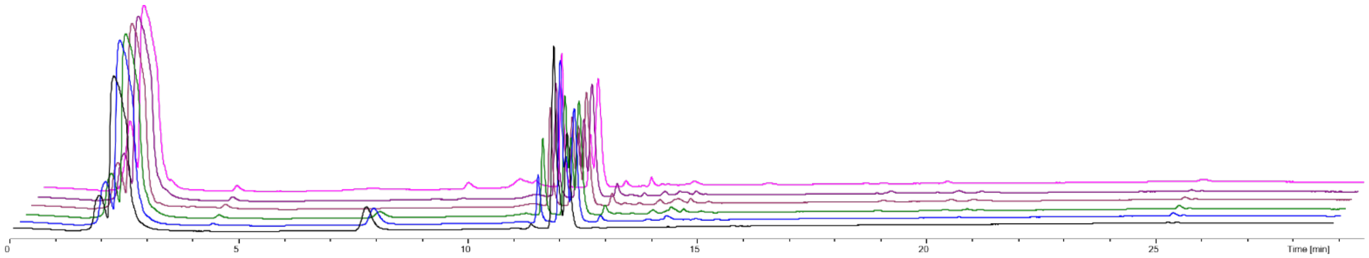

Figure S 66: HPLC chromatogram of compound **8a** after 0, 1, 2, 3, 4, 24 h (from front to back; conditions see experimental section).

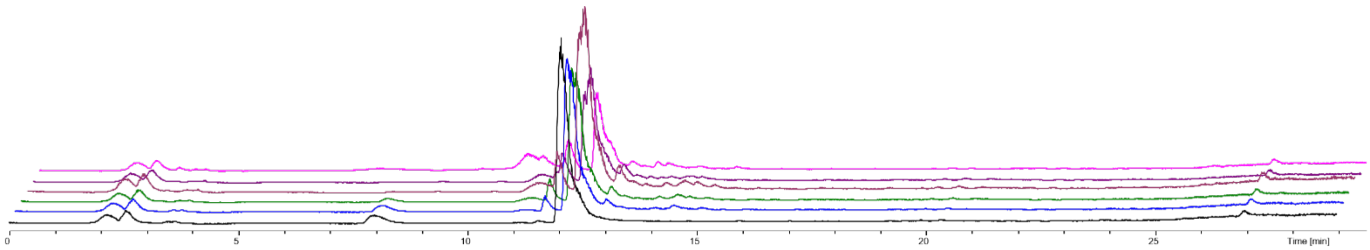

Figure S 67: HPLC-MS chromatogram of compound **8a** after 0, 1, 2, 3, 4, 24 h (from front to back; conditions see experimental section).

Theoretical studies

The optimised structures shown in Fig. S1 are part of the supporting information in xyz-format. The initial structures 1a-9a are named "min.xyz", the aqua-complexes "aqua.xyz" and the transition states "ts.xyz".

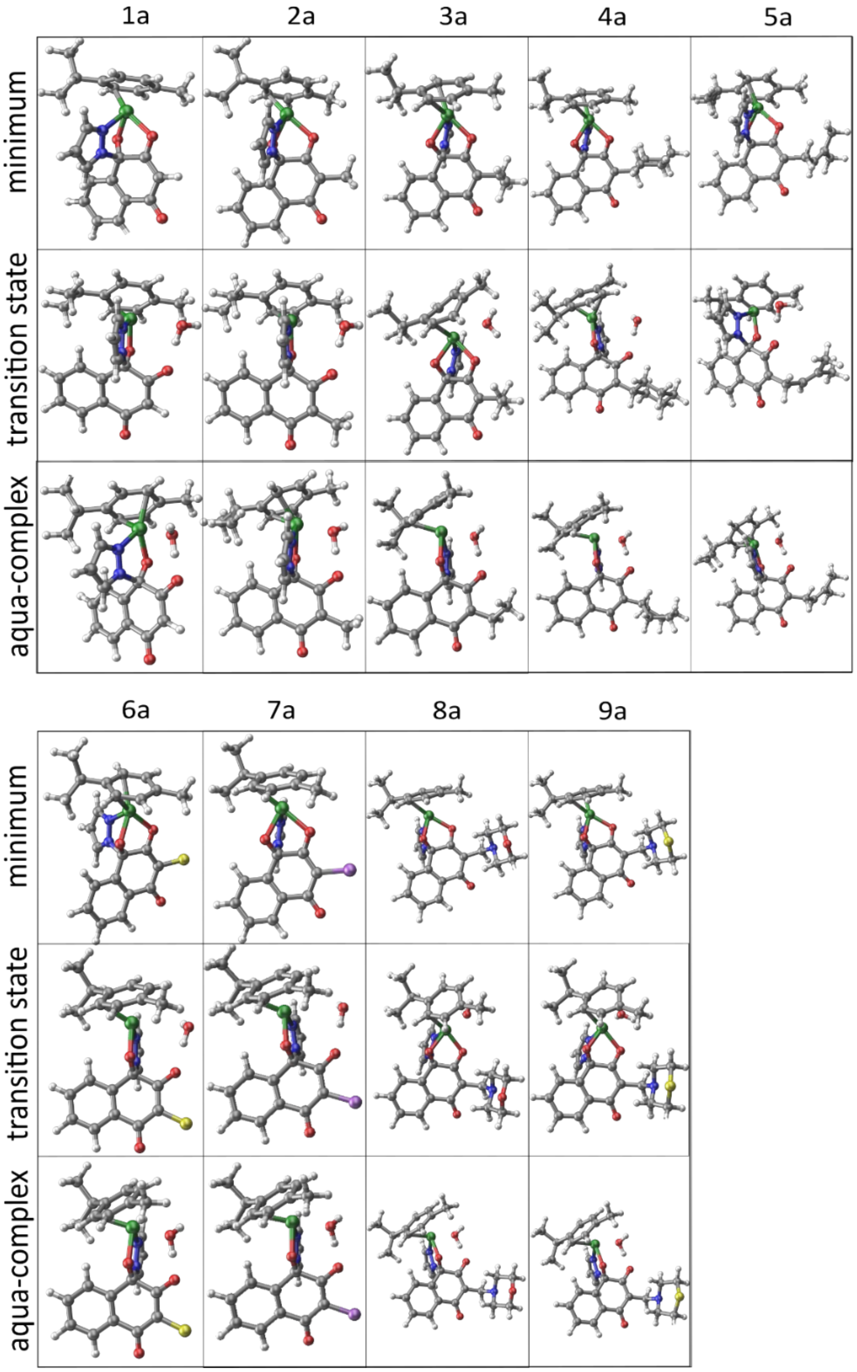

Figure S 68: Optimized structures at DFT/PBEh-3c level of theory of the synthesised complexes, the aqua-complex and the transition state structure.

Bond lengths

For comparison with the experimental values from Table 1 in the main text, the computed bond lengths are given in Table S1. Nomenclature is consistent with the main manuscript.

Table S 16: Bond distances of the optimised structures as obtained from X-ray spectroscopy.

	Ru-O1	Ru-O2	Ru-N2	C1-N1
**1a**	2.0312	2.0899	2.08186	1.4981
**2a**	2.0850	2.0295	2.08195	1.4988
**3a**	2.0310	2.0861	2.0800	1.4990
**4a**	2.0290	2.0788	2.0813	1.4982
**5a**	2.0295	2.0920	2.0797	1.4977
**6a**	2.0321	2.0985	2.0816	1.4960
**7a**	2.0310	2.1006	2.0817	1.4953
**8a**	2.0858	2.0879	2.0822	1.4978
**9a**	2.0260	2.0879	2.0819	1.4976

Table S 17: Bond distances of the transition state.

	Ru-O1	Ru-O2	Ru-N2	C1-N1
**1a**	1.9386	3.8859	2.0359	1.4734
**2a**	1.9386	3.8859	2.0359	1.4734
**3a**	1.9999	2.0821	2.0670	1.4931
**4a**	1.9351	3.9380	2.0337	1.4707
**5a**	1.9386	3.8598	2.0359	1.4771
**6a**	1.9447	3.7026	2.0359	1.4775
**7a**	1.9468	3.6828	2.0366	1.4786
**8a**	2.0388	2.0475	2.0048	1.5003
**9a**	2.0203	2.0608	2.0303	1.4991

Table S 18: Bond distances of the optimised aqua-complex

	Ru-O1	Ru-O2	Ru-N2	C1-N1
**1a**	2.0328	3.7290	2.0586	1.4956
**2a**	2.0386	3.7150	2.0595	1.4958
**3a**	2.0340	3.7178	2.0604	1.4955
**4a**	2.0335	3.7146	2.0598	1.4969
**5a**	2.0342	3.7296	2.0553	1.4951
**6a**	2.0346	3.7464	2.0573	1.4948
**7a**	2.0358	3.7448	2.0576	1.4940
**8a**	2.0342	3.7150	2.0611	1.4961
**9a**	2.0336	3.7124	2.0611	1.4946

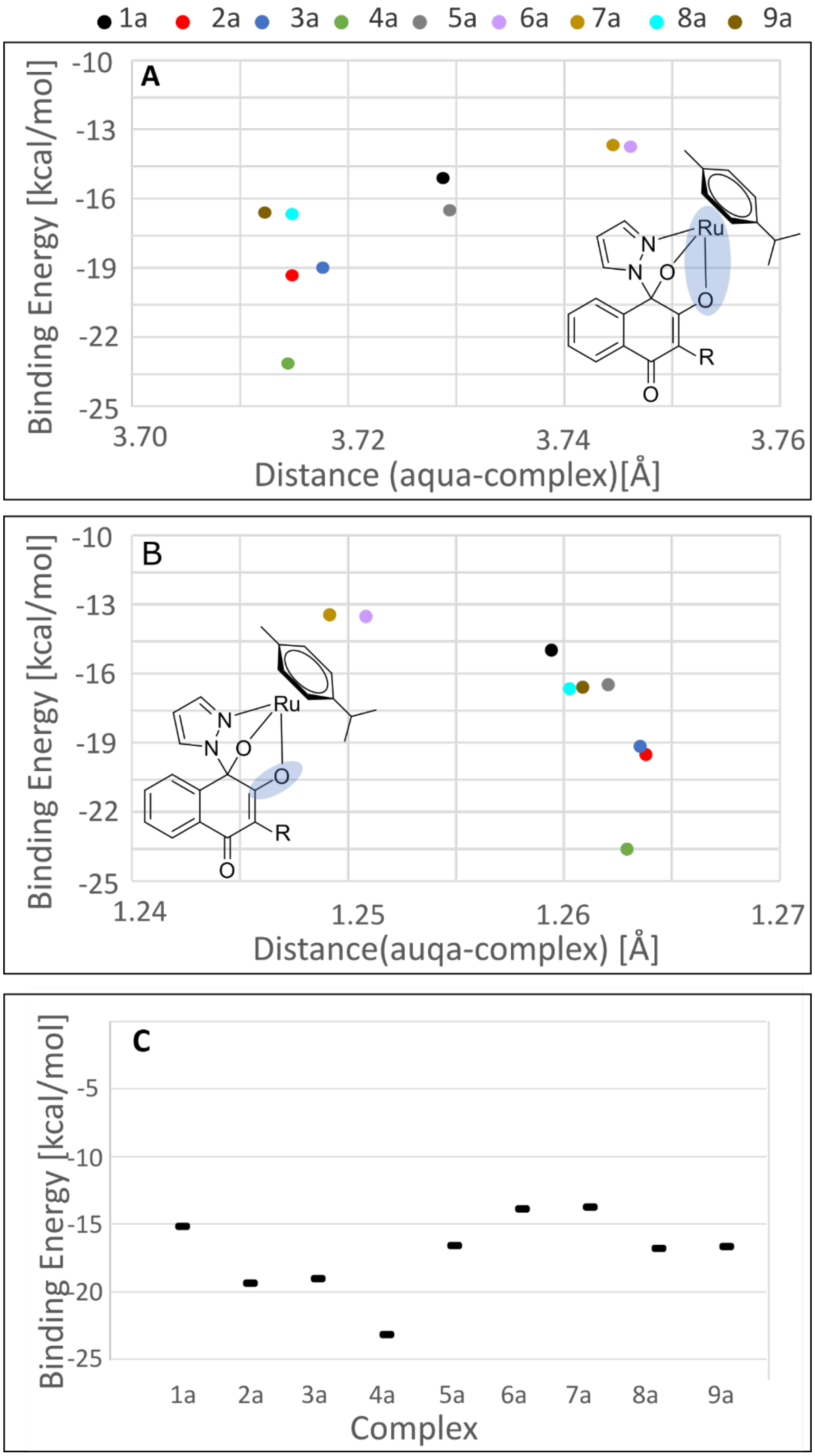

Figure S 69: **A** and **B**: Ru-*O2* and C-*O2* bond distance for the aqua-complex plotted against the binding energy. C: Relative binding energies plotted for all complexes (**1a-9a**)

Biological studies

MTT assay

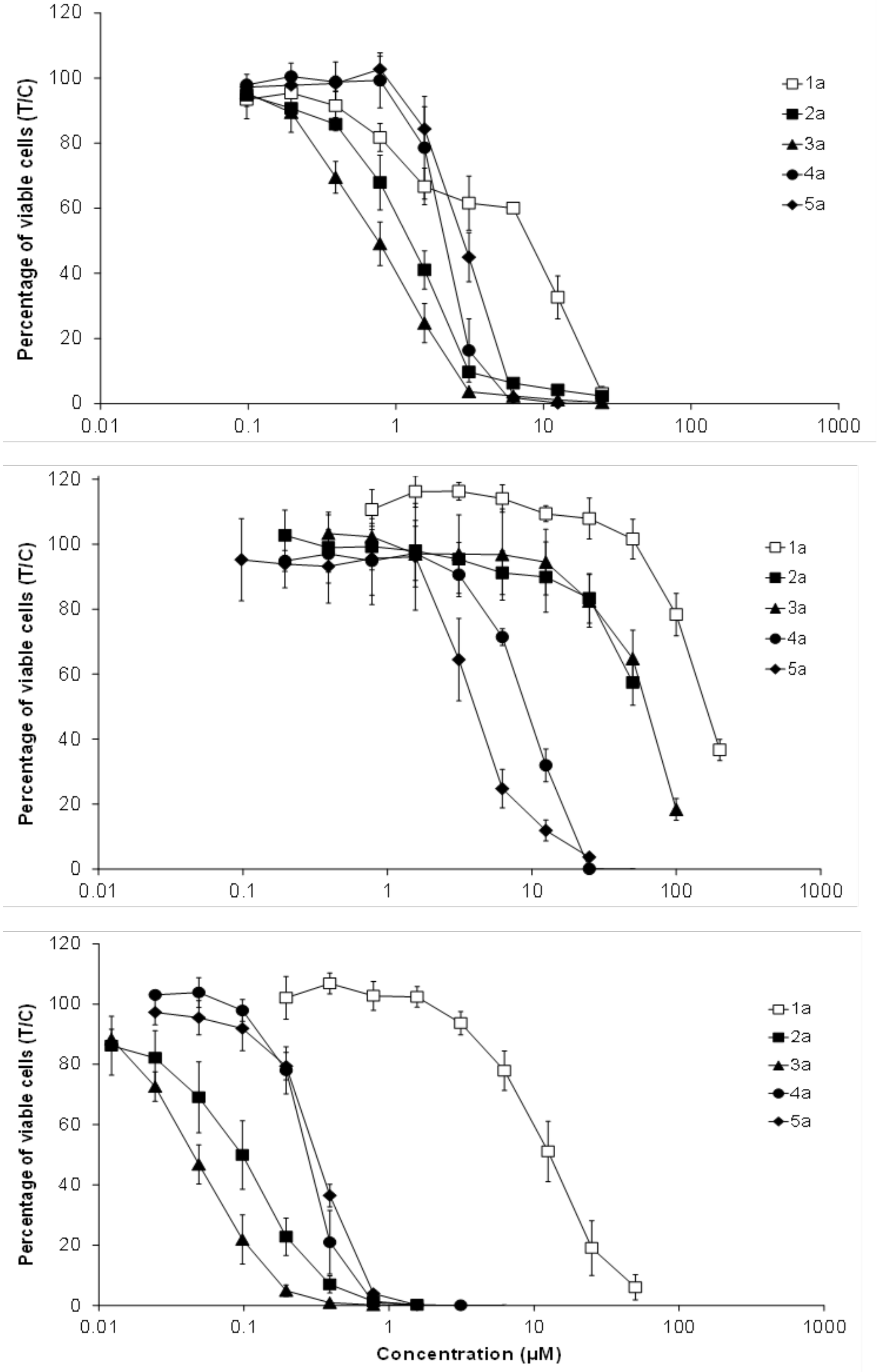

Figure S 70: Concentration–effect curves of **1a**–**5a** in A549 (top), CH1/PA-1 (middle) and SW480 (bottom) cells. Values are means ± standard deviations of at least three independent 96-h MTT assays.

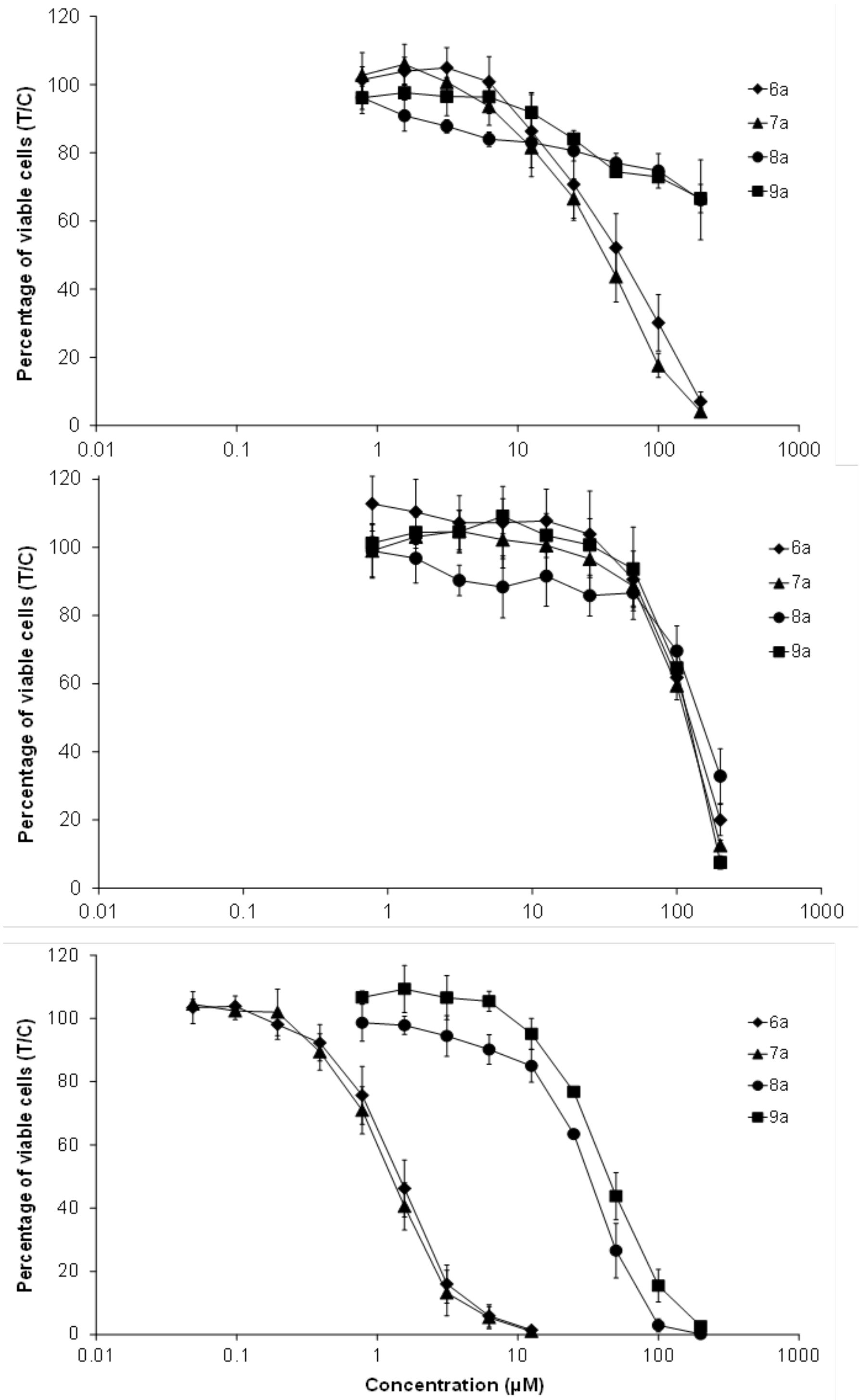

Figure S 71: Concentration–effect curves of **6a**–**9a** in A549 (top), CH1/PA-1 (middle) and SW480 (bottom) cells. Values are means ± standard deviations of at least three independent 96-h MTT assays.

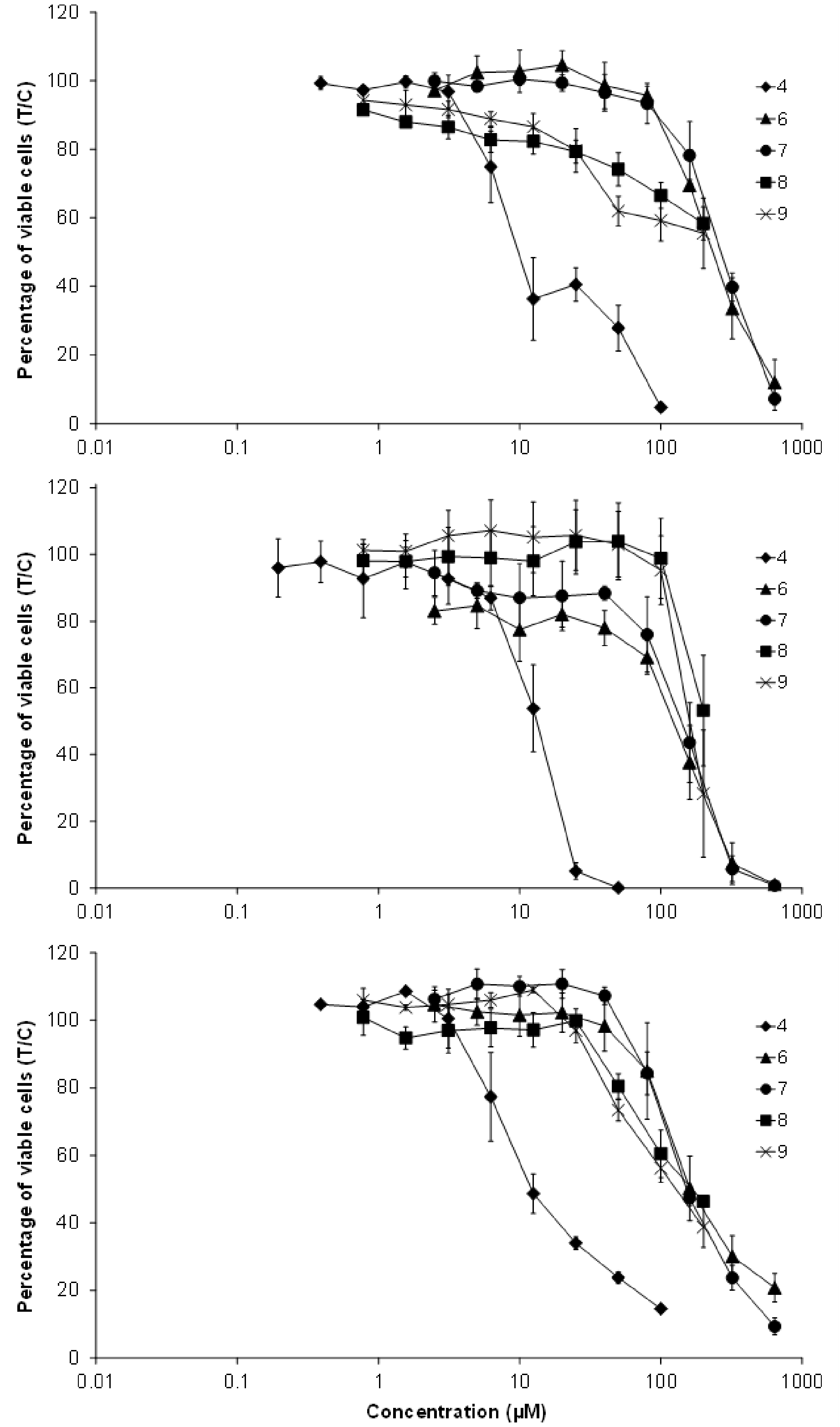

Figure S 72: Concentration–effect curves of **4**, **6**–**9** in A549 (top), CH1/PA-1 (middle) and SW480 (bottom) cells. Values are means ± standard deviations of at least three independent 96-h MTT assays.

ROS assay (DCFH-DA assay)

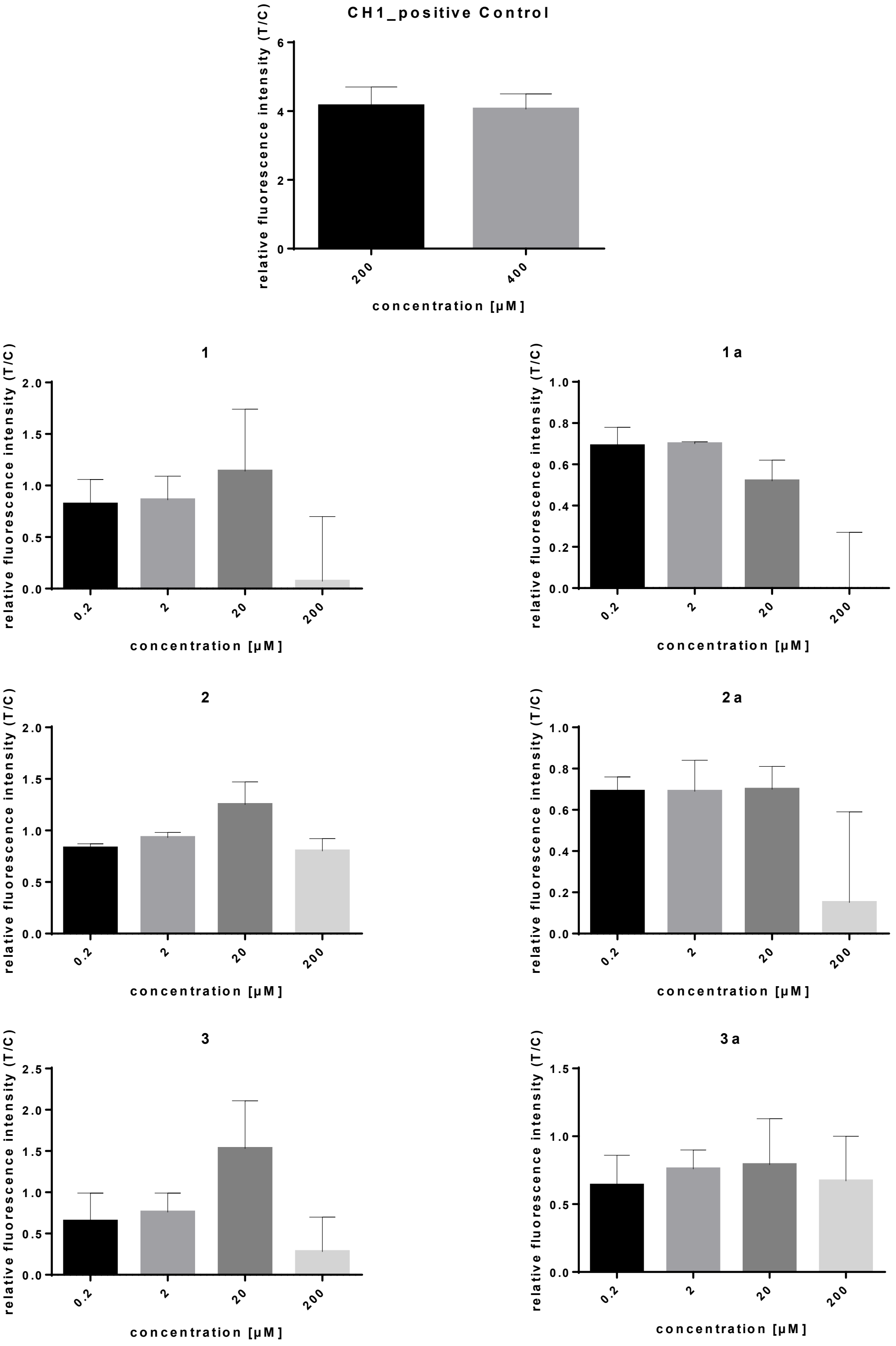

Figure S 73: ROS results for ligands (**1-3**) and complexes (**1a-3a**); exposure time 2h, CH1/PA-1 cancer cells.

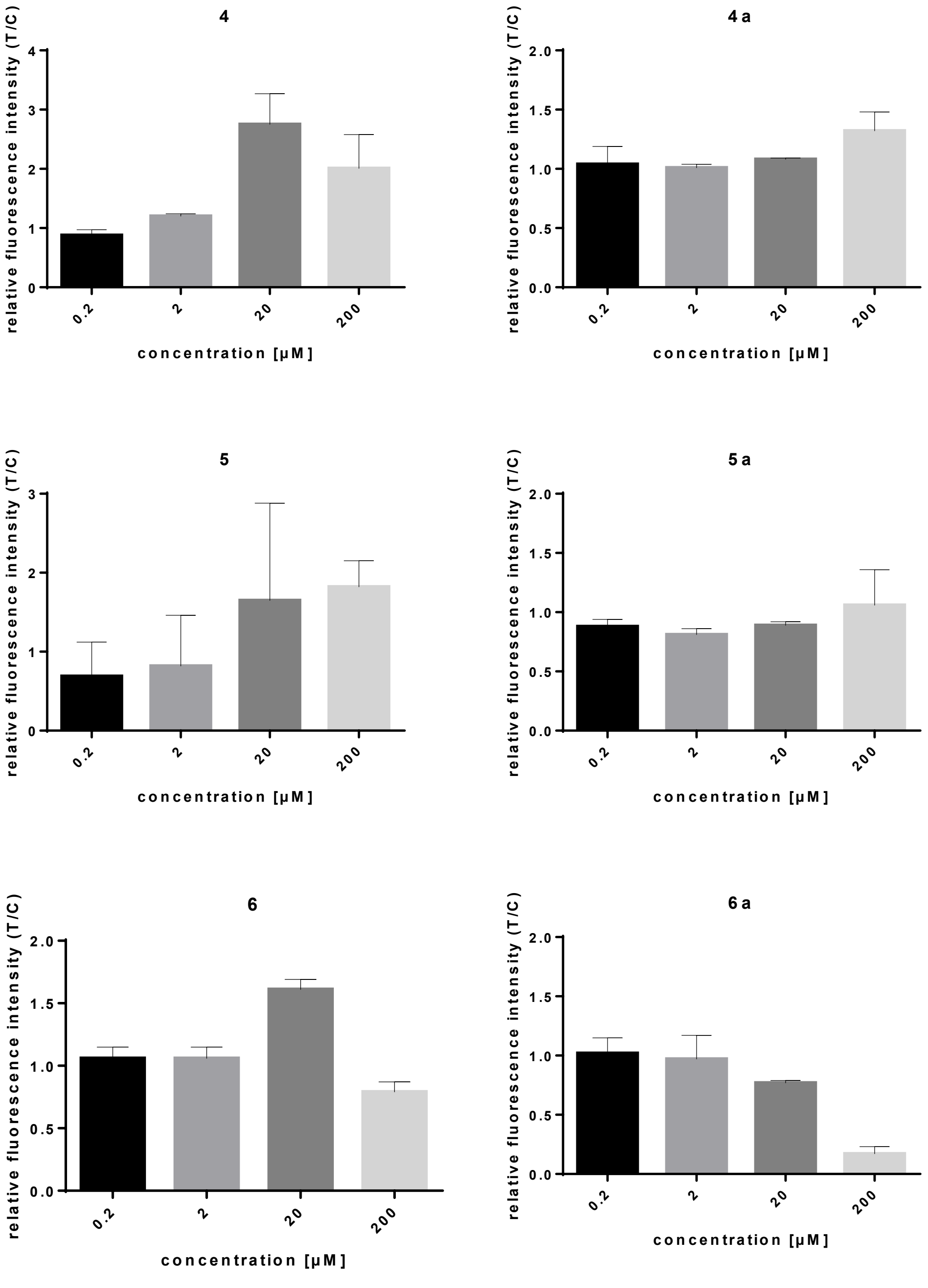

Figure S 74: ROS results for ligands (**4-6**) and complexes (**4a-6a**); exposure time 2h, CH1/PA-1 cancer cells.

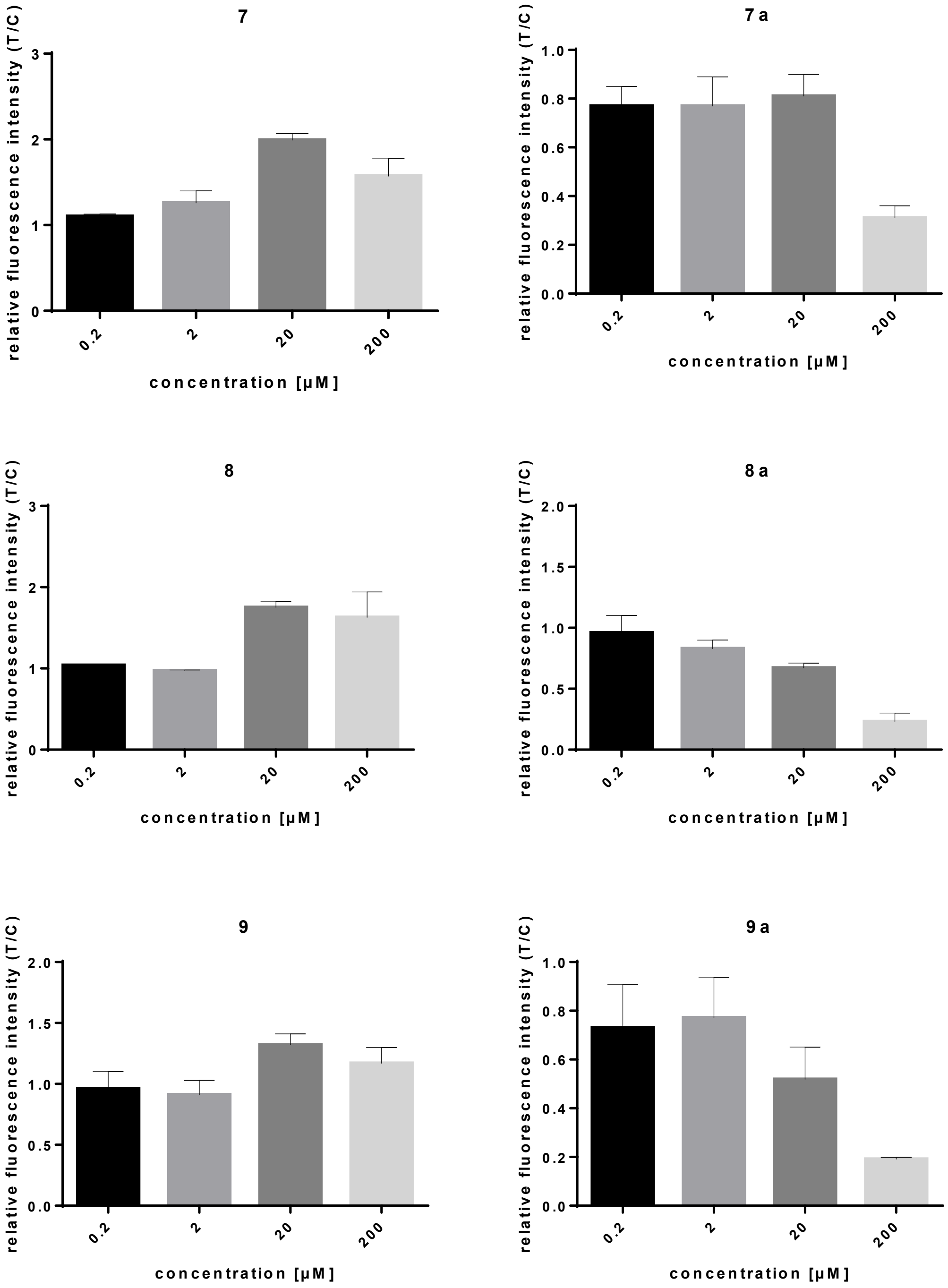

Figure S 75: ROS results for ligands (**7-9**) and complexes (**7a-9a**); exposure time 2h, CH1/PA-1 cancer cells.

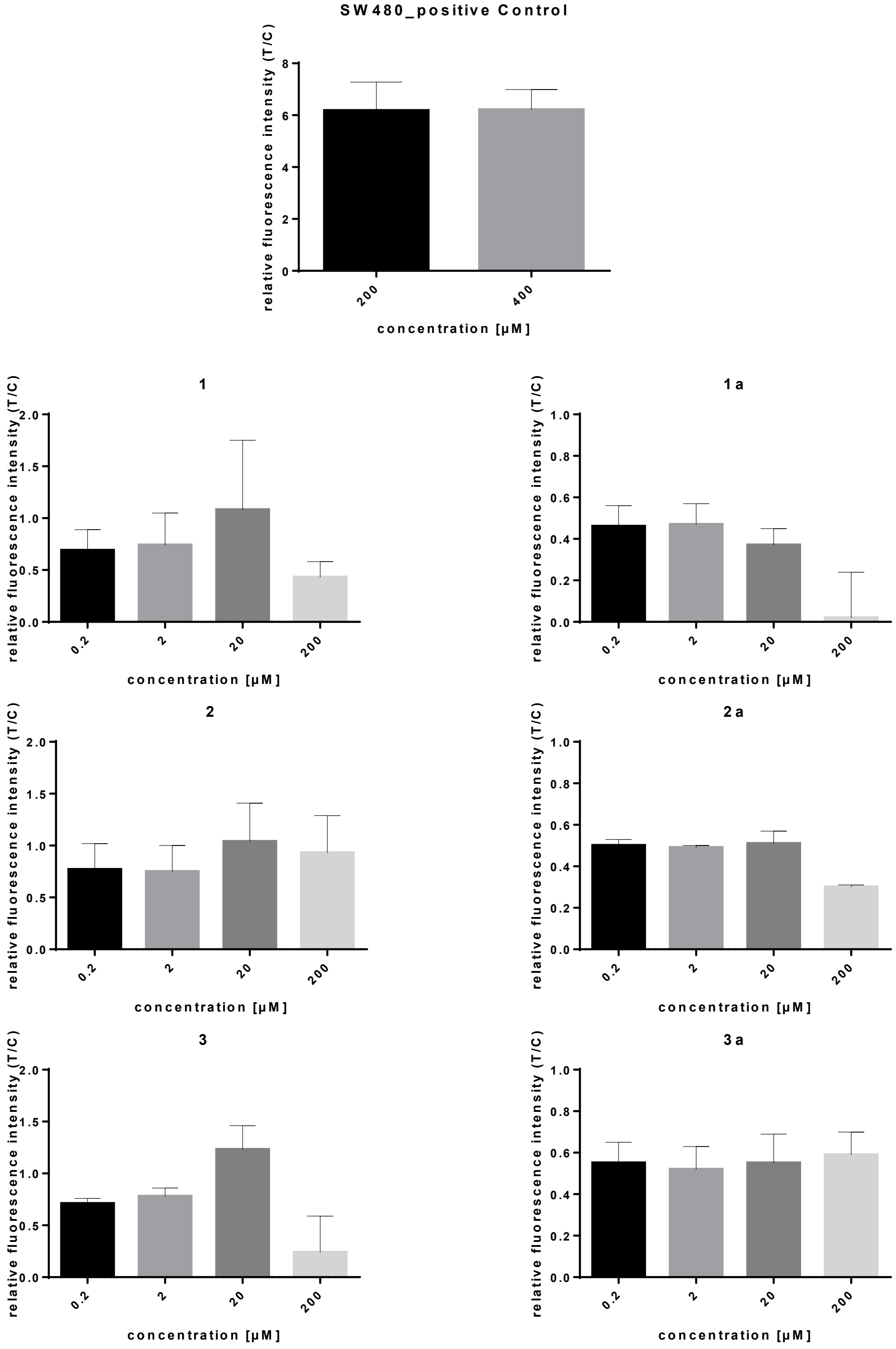

Figure S 76: ROS results for ligands (**1-3**) and complexes (**1a-3a**); exposure time 2h, SW480 cancer cells.

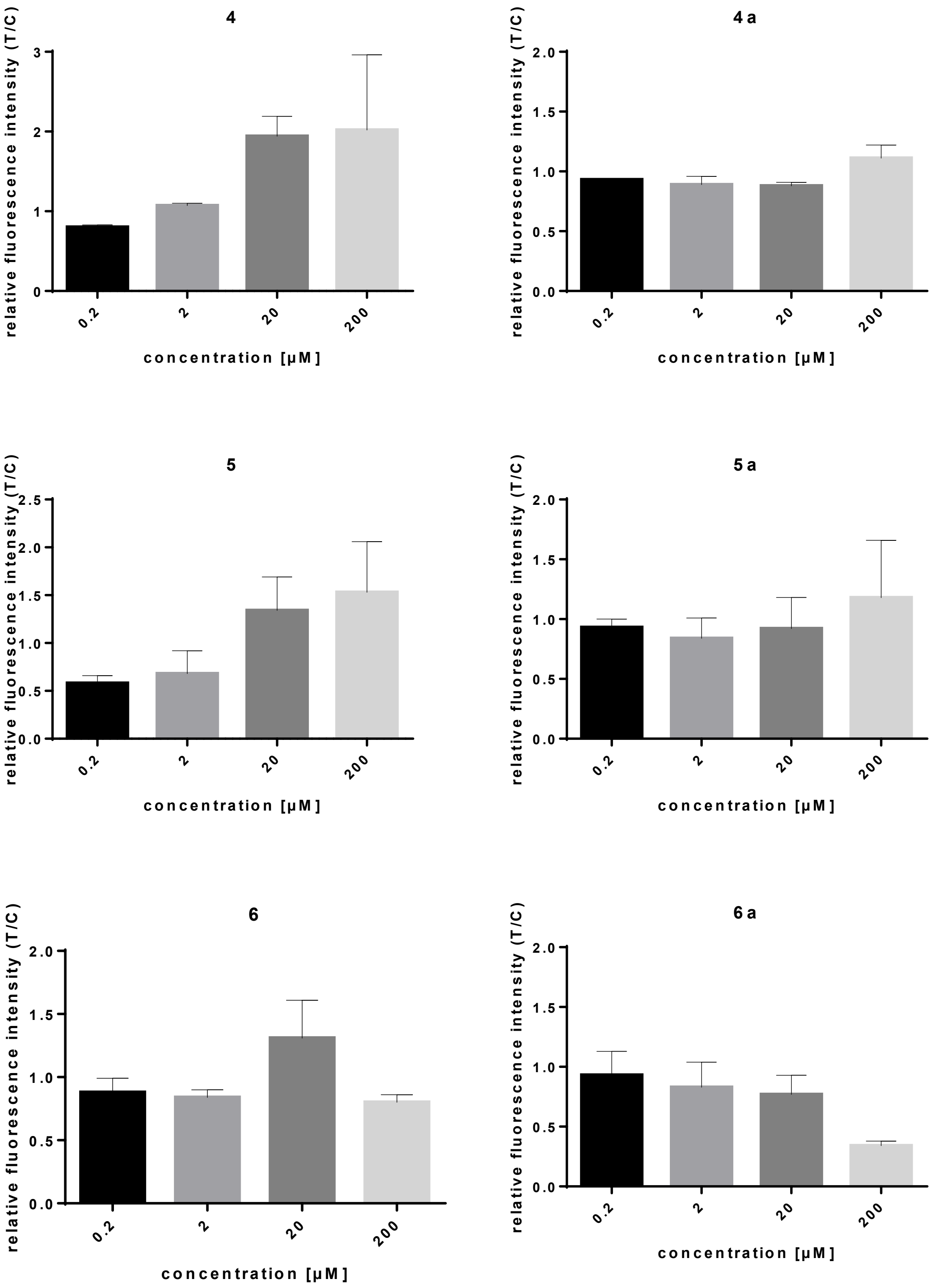

Figure S 77: ROS results for ligands (**4-6**) and complexes (**4a-6a**); exposure time 2h, SW480 cancer cells.

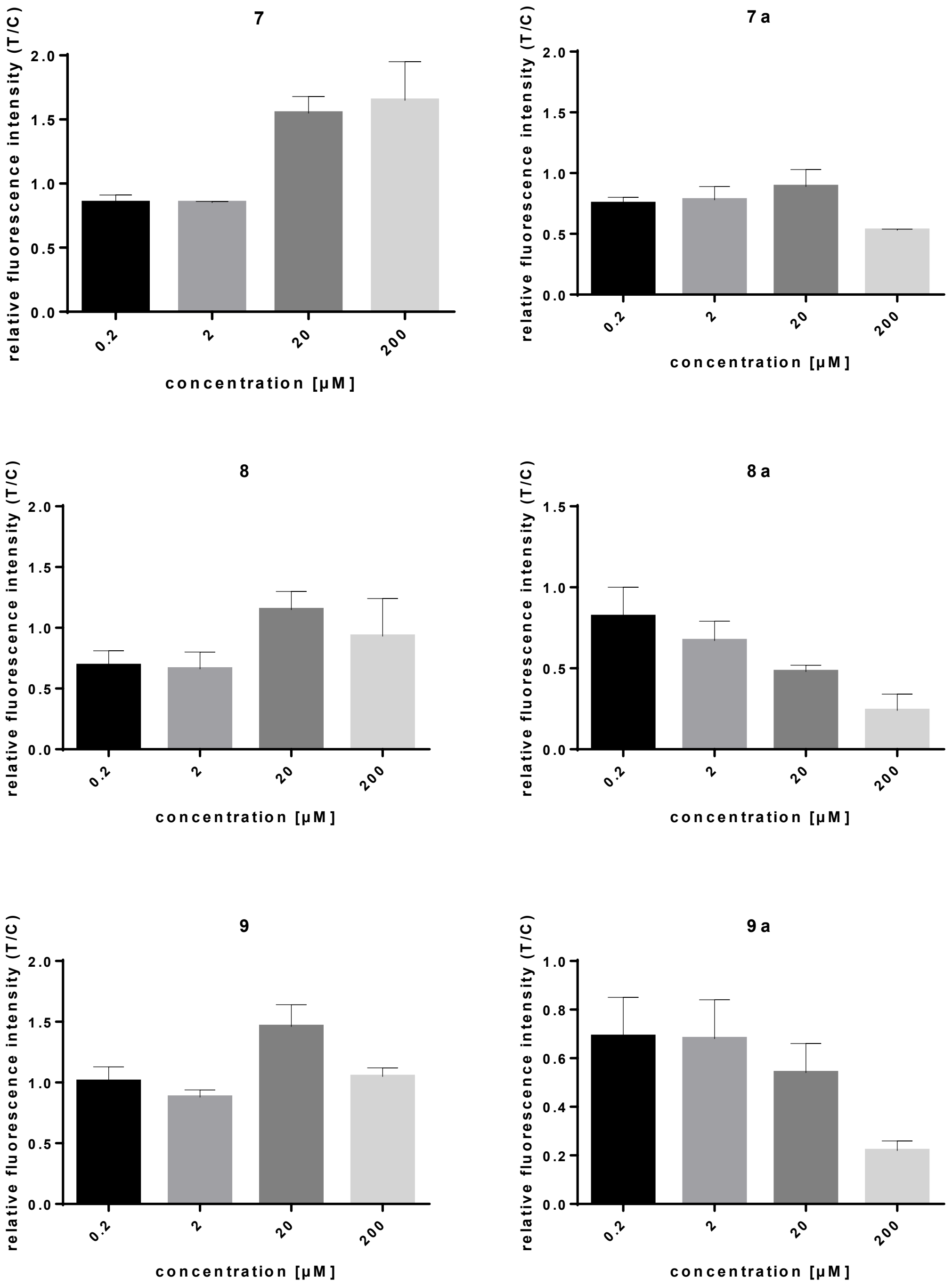

Figure S 78: ROS results for ligands (**7-9**) and complexes (**7a-9a**); exposure time 2h, SW480 cancer cells.

Plasmid assay

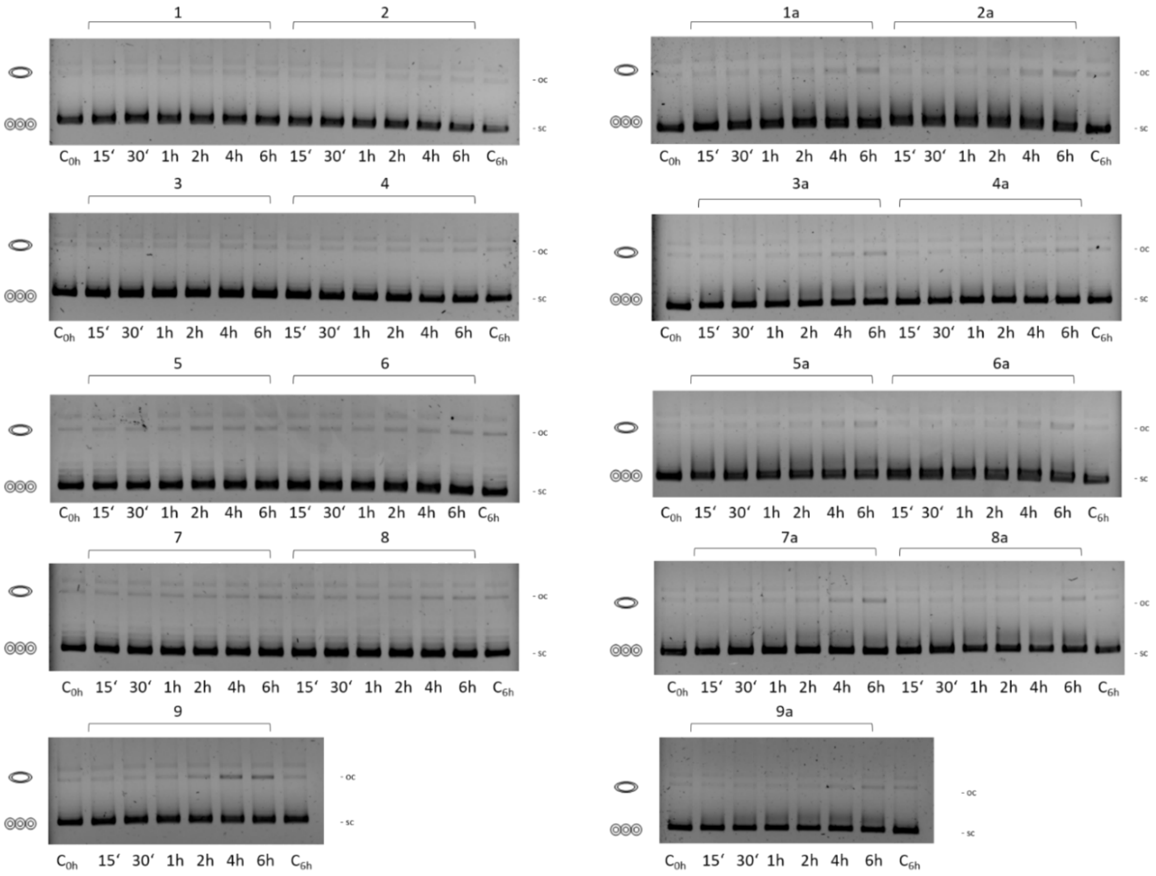

Figure S 79: Electropherograms of plasmid DNA interactions with 50 µM of compounds **1**–**9** and **1a**–**9a.** The pUC19 plasmid was incubated with the complexes for different time periods (from 15 min to 6 h) at 37 °C and the different plasmid forms were separated by agarose gel electrophoresis. 'C0h' corresponds to an untreated, non-preincubated control at the time point of gel loading, while 'C6h' corresponds to an untreated, 6 h incubated control; oc, open circular; sc, supercoiled.

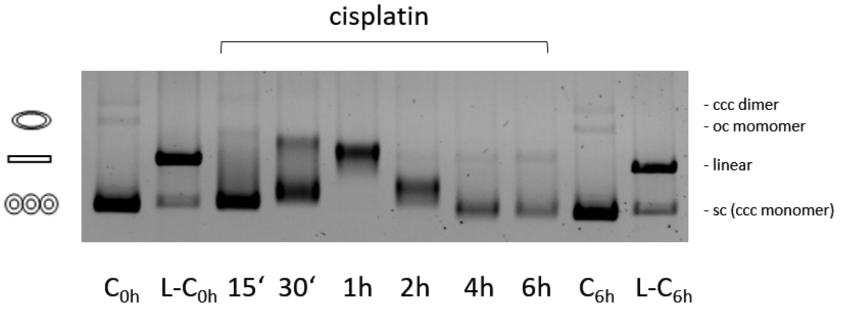

Figure S 80: Electropherogram of dsDNA plasmid pUC19 incubated with 50 µM of cisplatin for different incubation times (from 15 min to 6 h) at 37 °C. Four different types of the plasmid were analysed by gel electrophoresis: supercoiled (sc), linear, open circular (oc) and dimers of covalently closed circles (ccc). 'C_{0h}' (pUC19 plasmid) and 'L-C_{0h}' (pUC19 linear) correspond to the untreated, non-preincubated control, 'C_{6h}' (pUC19 plasmid) and 'L-C_{6h}' (pUC19 linear) correspond to an untreated, 6 h incubated control.

References

1. AXS, B. *Bruker SAINT* v.838B Copyright(C);: Bruker AXS, 2005-2019.
2. Sheldrick, G. M. Sadabs. *University of Göttingen, Germany* **1996**.
3. Dolomanov, O. V.; Bourhis, L. J.; Gildea, R. J.; Howard, J. A. K.; Puschmann, H. OLEX2: a complete structure solution, refinement and analysis program. *J. Appl. Crystallogr.* **2009,** 42 (2), 339-341 DOI: doi:10.1107/S0021889808042726.
4. Hubschle, C. B.; Sheldrick, G. M.; Dittrich, B. ShelXle: a Qt graphical user interface for SHELXL. *J. Appl. Crystallogr.* **2011,** 44 (6), 1281-1284 DOI: doi:10.1107/S0021889811043202.
5. Sheldrick, G. SHELXS v 2008. **2008**.
6. Sheldrick, G. Crystal structure refinement with SHELXL. *Acta Crystallogr., Sect. C: Struct. Chem.* **2015,** 71 (1), 3-8 DOI: doi:10.1107/S2053229614024218.
7. Spek, A. Structure validation in chemical crystallography. *Acta. Crystallogr., Sect. D: Struct. Biol.* **2009,** 65 (2), 148-155 DOI: doi:10.1107/S090744490804362X.
